# Bio-informatic analysis of CRISPR protospacer adjacent motifs (PAMs) in T4 genome

**DOI:** 10.1186/s12863-022-01056-8

**Published:** 2022-06-02

**Authors:** Omar Rawashdeh, Rabeah Y. Rawashdeh, Temesgen Kebede, David Kapp, Anca Ralescu

**Affiliations:** 1 Department of Electrical Engineering and Computer Sciences, University of Cincinnati, Cincinnati, OH 45221 USA; 2 Department of Biological Sciences, Yarmouk University, Shafiq Irshidat Street, Irbid, 21163 Jordan; 3 AFRL, WPAFB, Dayton, OH 45433 USA

**Keywords:** CRISPR-Cas9, Genomic distribution, PAMs, Promoters, Transcription, T4 phage

## Abstract

**Background:**

The existence of protospacer adjacent motifs (PAMs) sequences in bacteriophage genome is critical for the recognition and function of the clustered regularly interspaced short palindromic repeats-Cas (CRISPR-Cas) machinery system. We further elucidate the significance of PAMs and their function, particularly as a part of transcriptional regulatory regions in T4 bacteriophages.

**Methods:**

A scripting language was used to analyze a sequence of T4 phage genome, and a list of few selected PAMs. Mann-Whitney Wilcoxon (MWW) test was used to compare the sequence hits for the PAMs versus the hits of all the possible sequences of equal lengths.

**Results:**

The results of MWW test show that certain PAMs such as: ‘NGG’ and ‘TATA’ are preferably located at the core of phage promoters: around -10 position, whereas the position around -35 appears to have no detectable count variation of any of the tested PAMs. Among all tested PAMs, the following three sequences: 5’-GCTV-3’, 5’-TTGAAT-3’ and 5’-TTGGGT-3’ have higher prevalence in essential genes. By analyzing all the possible ways of reading PAM sequences as codons for the corresponding amino acids, it was found that deduced amino acids of some PAMs have a significant tendency to prefer the surface of proteins.

**Conclusion:**

These results provide novel insights into the location and the subsequent identification of the role of PAMs as transcriptional regulatory elements. Also, CRISPR targeting certain PAM sequences is somehow likely to be connected to the hydrophilicity (water solubility) of amino acids translated from PAM’s triplets. Therefore, these amino acids are found at the interacting unit at protein-protein interfaces.

**Supplementary Information:**

The online version contains supplementary material available at 10.1186/s12863-022-01056-8.

## Background

The clustered regularly interspaced short palindromic repeats-Cas (CRISPR-Cas) defensive system is an acquired mechanism in prokaryotes which is analogous to RNAi in eukaryotes [1]. The CRISPR machinery is a complex immune strategy that is continually evolving in the bacterial genome to accommodate the rapid changes in the nucleic acids of the infecting phage [2]. According to the polythetic classifications, there are six CRISPR-Cas types: I, II, III, IV, V, VI [2].

Bacteriophages (also called phages, are viruses that infect bacteria) have two life forms. The lytic viruses hijack the cellular machinery of bacteria for the synthesis of viral nucleic acids and proteins. After their assembly, the new viruses are released through cell lysis. In contrast, the lysogenic bacterial virus stays in dormancy in its host cell by integrating its DNA into the host’s genome, thereby contributing to horizontal gene transfer, and causing bacteria to gain new traits encoded by the integrated genes [3, 4].

**Fig. 1:**
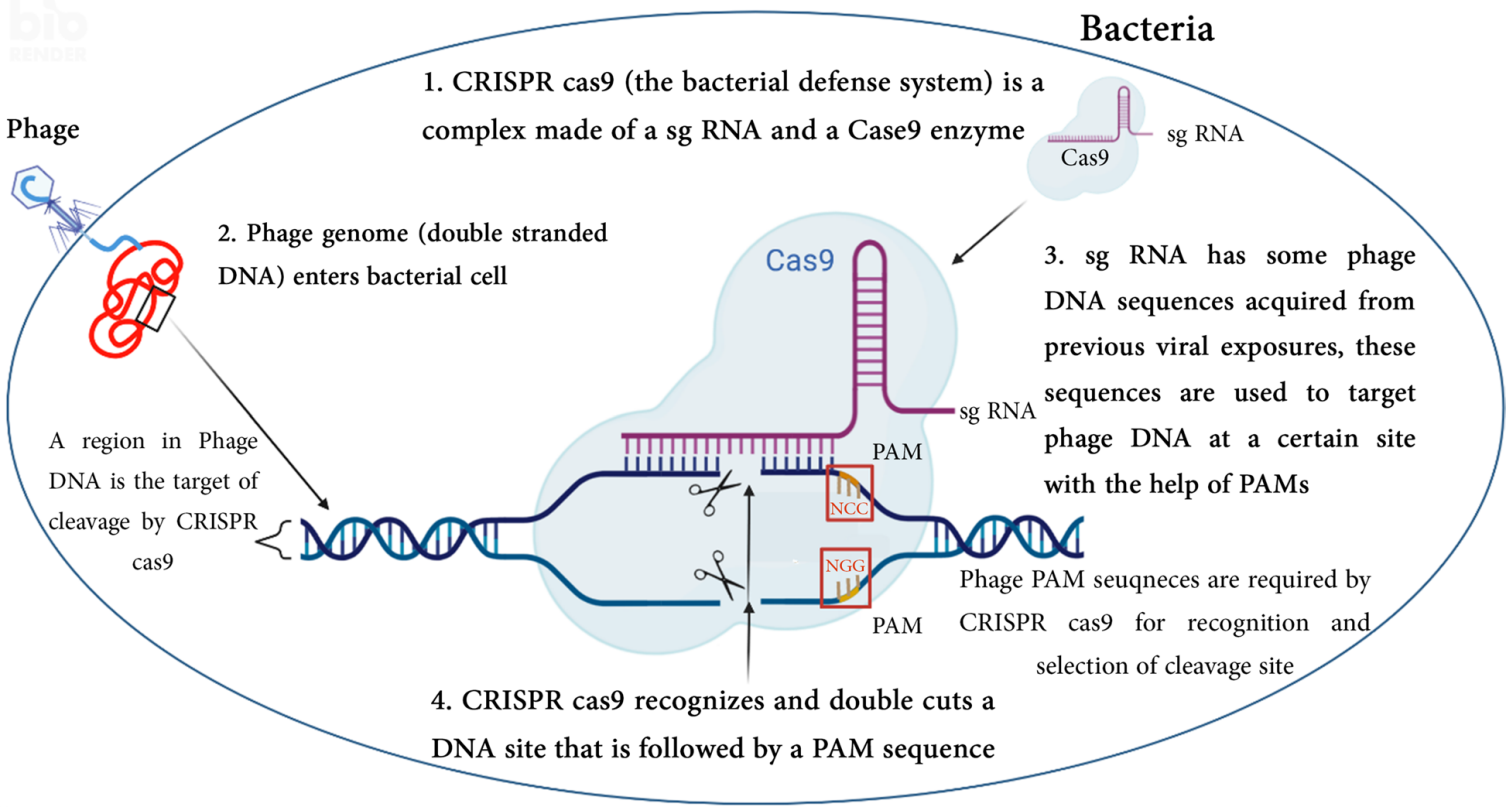
A schematic illustration of the components and mechanism of CRISPR-Cas9. 1. CRISPR-Cas9 is a fusion between Cas9 nuclease and single guiding RNA (sg RNA). Spacer sequences in sg RNA are phage DNA sequences that were integrated into bacterial DNA from previous exposures. 2. A latter phage attack causes the injection of its DNA genome inside the bacterial cell. 3. CRISPR-Cas9 complex recognizes the target cleaving site in phage genome by the help of the protospacer-adjacent motif (PAM) sequence, at this step CRISPR-Cas9 binds the phage DNA sequences that are complementary to sg RNA which are located the upstream genomic sequence of PAM. 4. Cas9 cuts the double strands upstream of the PAM to induce DNA double-strand breaks. Different PAM sequences are used by several types of CRISPR systems and the PAM sequence NGG was used for illustration. Created with BioRender.com

The integrated viral DNA sequences in bacterial genome are interspaced by repetitive sequences termed spacers. This pattern of clustering immune genes into one molecular unit, is known as CRISPR array [5]. A CRISPR Spacer is transcribed and processed into a small CRISPR RNA (crRNA), often called small guide RNA (sgRNA). Because of the sequence homology between a spacer and a part of viral genome, the transcribed sgRNA recognizes the complementary viral sequence. The subsequent stage is the cleavage of the recognized sequence by Cas nucleases (Fig. 1). These enzymes are encoded by Cas genes flanking CRISPR locus [6]. Upon viral infection a new spacer sequence is cleaved and integrated into the CRISPR array via horizontal gene transfer.

A significant site called PAM (protospacer-adjacent motif) is critical in CRISPR-Cas selective recognition for its target foreign protospacer sequence [7]. The well documented characteristic of all CRISPR-Cas types is their ability to find and cleave target invader sequences upon the recognition of the highly conserved short PAMs [7]. NGG PAM is a short protospacer adjacent motif, that is recognized mainly by Cas9 of *Streptococcus pyogenes*. NAG PAM is recognized to a lesser extent by Cas9. CTT is another simple PAM, which is recognized by Cas1 of *Escherichia coli*. TTTV is the recognition PAM for Cpf1 (Cas12a) of *Acidaminococcus* sp. Another role of PAMs is in the discrimination of self from non-self during the CRISPR immune response. Non-self-CRISPR immunity distinguishes the foreign DNA, the foreign protospacer, from the self-spacer DNA and destroy them [8, 9].

PAMs length varies from two to six nucleotides, but the highly conserved ones are made up of three or four nucleotides [10]. PAMs are analyzed more frequently in relation to their CRISPR type specific motifs [7]. Located immediately, or one base after, PAMs are adjacent to protospacer invader sequences, which are never adjoining to prophages within the host CRISPR loci.

Bioinformatics and computational biology are necessary for the analysis of PAMs performance in bacteriophages. The existing sequences databases are exclusively for the following CRISPR elements: sgRNA, to target human genes [11, 12], CRISPR loci, and associated Cas proteins [13–15]. None of the databases we encountered analyze PAMs sequences or elucidated their relative importance to bacteriophages. The most well-known PAMs sequences in viral nucleic acids which are highly targeted by CRISPR-Cas system are shown in Table 1.

**Table 1:** PAM motifs according to CRISPR-Cas type but regardless of the contained phages

CRISPR-Cas system types	From Organism	PAM Sequence (5' to 3')	Reference
II-A I-C IV	*Streptococcus pyogenes* *S. agalactiae* *Listeria monocytogenes* *Francisella novicida* (FnCas9)	NGG NAG (A/T)GG	[7, 16, 17]
		NGG	[18]
II-A	*Staphylococcus aureus*	NGRRT NGRRN NNGRRT TTGGGT	[19–21]
II- C	*Neisseria meningitidis*	NNNNGATT	[16]
II-A	*Streptococcus thermophilus*	NNAGAAW NGGNG	[16, 17, 22, 23]
V-A II-A	*Lachnospiraceae bacterium*	TTTV TCTA	[24]
V-A Cas12a (AsCas12a, AsCpf1)	*Acidaminococcus sp.*	GTTV GCTV TATA (eng. Cpf1) TTTV	[10, 25]
I-E	*Escherichia coli*	C(T/A)T	[17, 26, 27]
I-E	*Escherichia coli*	5’-ATG-3’ 5’-AAG-3’ Its complement 5′-CTT-3′	[28, 29]
I-E	*Escherichia coli*	AWG	[30]
I-A	*S. solfataricus*	TCN CCN	[31–33]

The link between bacteriophages and their host bacteria is not well determined, and it becomes particularly challenging with the coevolution of CRISPR immunity in bacteria and attacking phages. A study done by Garneau et al. 2010 showed that a particular phage is specifically infecting a particular bacterium such as the phage 2972 infection of *Streptococcus thermophilus* [22]. However, in many other cases the situation is unclear as some phages have a broad host range and they can infect any type of bacteria [4, 8, 31]. By adopting the later as a hypothesis for this study, we consider the T4 phage as a model phage that is recognizable by various CRISPR-Cas bacterial systems. Accordingly, T4 phage has multiple target bacteria and thus, T4 is the pool of different PAMs sequences.

Many to many is the relationship that exists in the following two pairs: bacterial strain with CRISPR-Cas type and CRISPR-Cas type with the recognized PAM. Indeed, CRISPR type II-A exists in both *S. pyogenes* and *S. thermophilus* (Table 1) [34].

Based on their role in phage growth and replication, some genes were identified as essential, and the others were non-essential genes. A certain regulatory DNA sequence that lies at a distant site from the gene is called the promoter [35]. A genome promoter is a specific DNA sequence where the transcription initiation complex binds and transcription begins to form the RNA molecule. A promoter contains two short elements - the core and the upstream elements. Different genes are expressed over the time of viral infection (phage life cycle) and therefore three types of promoters are used for gene expression - early, middle, and late - promoters for transcribing early, middle and late genes respectively [36].

There are two distinct subsites of phage core promoter that are around two positions: at -10 and at -35. Both regions are found upstream of the transcription start site, known as TSS, that is given the coordinate +1. The core promoter at -10 and at -35 have hexanucleotide sequences: 5’-TATAAT-3’ and 5’-GTTTAC-3’ respectively [37].

A previous study [35] did a comprehensive analysis of T4 genome. All T4 promoters have the consensus sequence of 5’-TATAAT-3’ at -10 (-7 to -12). Early promoters (Pe) have -31 to -36 conserved sequence. The middle promoters (Pm) have a highly conserved sequence of 5’-GCTT-3’ between -27 and -32 (called Mot box). The upstream element of both Pe and Pm is located at position -42 and it is rich with A bases.

PAM sequences would be expressed as amino acids only if they are occurring inside genes. Depending on the start base of a gene reading frame, a certain PAM DNA generates different triplet codons for translation into amino acids during protein synthesis. Quaternary complex proteins are large biological molecules made of more than one chain of polypeptides. The overall folding of a tertiary polypeptide is determined to some extent by the level of water interaction with certain atomic groups of a polypeptide called the side chains (R group). High water-soluble residues mean the linked amino acids are hydrophilic and these are most likely located on the protein surface, and they are more accessible to solvent, while low water-soluble residues are found buried in the core of a polypeptide. Hydrophilic residues have higher values of average surface accessibility (ASA) [38].

In this work, we did investigate some of the possible reasons for CRISPR selection of PAM sequences, specifically, the gene essentiality and function, surface exposure of amino acids, and whether the conservation of regulatory sequences in T4 Phages promoters raises their chance of being chosen as PAMs in CRISPR-Cas.

## Methods

### Data

The T4 genome data was downloaded from the NCBI Entrez Genome site (https://www.ncbi.nlm.nih.gov/nuccore/NC_000866.4/), NCBI Reference Sequence: NC_000866.4. The data, in FASTA formats, is of size 168,903 bp. The file is about 170 KB.

All data regarding genes start/end positions and their essentiality, and promoters’ locations and directions, were obtained from what was gathered by Miller’s study [35].

### Features

In addition to the typical promoter motifs: the -35 and the -10 elements, the collected promoter data comprises 22 different motifs, 12 of which are recognized by the high repetitive scores. Data features include the scores of the promoter motifs at -35 and -10, as well as information about the UP elements (upstream promoter). In addition to the previous information, genes coding regions were used for the analysis of PAMs.

### Analysis of promoters

Promoter sequences were divided into six parts, according to the location of the nucleotides in it, the groups are, 0-10, 10-20, 20-30, 30-40, 40-50, 50-60 upstream of transcription starting site. This division is to keep track of preserved sequences that matches the selected PAMs, to a degree.

We counted the copy number of PAM throughout the promoters while also keeping track of the location of the PAM within the promoters. Each position of PAM was reported in the three classes of promoters. Plotting was performed in a way that allows a comparison of the PAMs with each other, and the different groups for each PAM, to show reliability and conservation of the target.

In order to statically establish a ground, for our proposed idea of “CRISPR favoring conserved regulatory sequences in Promoters as PAMs”, all possible short nucleotides sequences were used to compare their sequences hits with that of the PAMs. The short sequences were limited in lengths from 2 nucleotides, as the shortest, to 6 nucleotides, as the longest. The base ‘N’ was removed (which stand for any nucleotide) on the edges of the PAMs. We called this list of sequences ‘the control group’. To decide on which test to use, we plotted the histogram of the sequence hits of the list and calculated the variances of the groups as well.

Since a PAM with the sequence ‘GGNG’ is expected to be found more often in promoters than a PAM that has no ‘N’ nucleotide, such as ‘GGTG’, we changed the PAMs sequences to only use the 4 nucleotides (A,T,C and G), to have two distributions for the same variable, which would allow us to run tests on the distributions from two groups (otherwise we will have two distributions with two different variables). So, for example the PAM ‘GGNG’, became the following four PAMs, ‘GGAG’, ‘GGTG’, ‘GGCG’ and ‘GGGG’.

We used the Mann-Whitney Wilcoxon (MWW) test, to compare the populations (PAMs vs Control). However, since one key assumption of the MWW test is the independence of the samples, we removed any sequence in the PAMs group from the control group.

### Analysis of gene sequences

Each nucleotide in a gene was selected as a starting nucleotide for any of the PAMs by reading the nucleotides downstream of it, such that if the PAM is five nucleotides long, we then compared the five nucleotides with the PAM, starting at the current nucleotide. In addition to that, and because DNA is a double helix molecule, we checked the existence of PAMs on the complementary strand (the other strand of DNA), by changing the PAM to its complement, then reversed it to account for the change of direction from the 5′ to 3′ to the 3′ to 5′ on the opposite strand. After that, we checked if the reversed complementary version of the PAM occurs at the current location. For example, ATG becomes TAC, which after reversal, turns into CAT, thus for the PAM ATG, we did increment the count by one if we find at ATG or CAT at the current position. Outliers in the data were considered significant targets, such that if the count for the PAM in the gene is larger than the mean and deviate greatly from it, we took it as an important targeted gene. For each PAM we calculated the fraction of essential genes from those that we deemed important for that PAM.

### PAM relation to surface accessibility

Each PAM was matched it to a list of amino acids, based on what nucleotides triplets we could get from the different reading frames. So, for a PAM with the nucleotides NATG, we got the following codons: AAT, TAT, CAT, GAT, ATG. After which we translated these codons to their amino acids’ counterpart. Using the mean and median average surface accessibility values from Lins’s study [39], we calculated the average value across these codons and the maximum value out of them to have an ASA value for each PAM.

## Results and Discussion

**Fig. 2:**
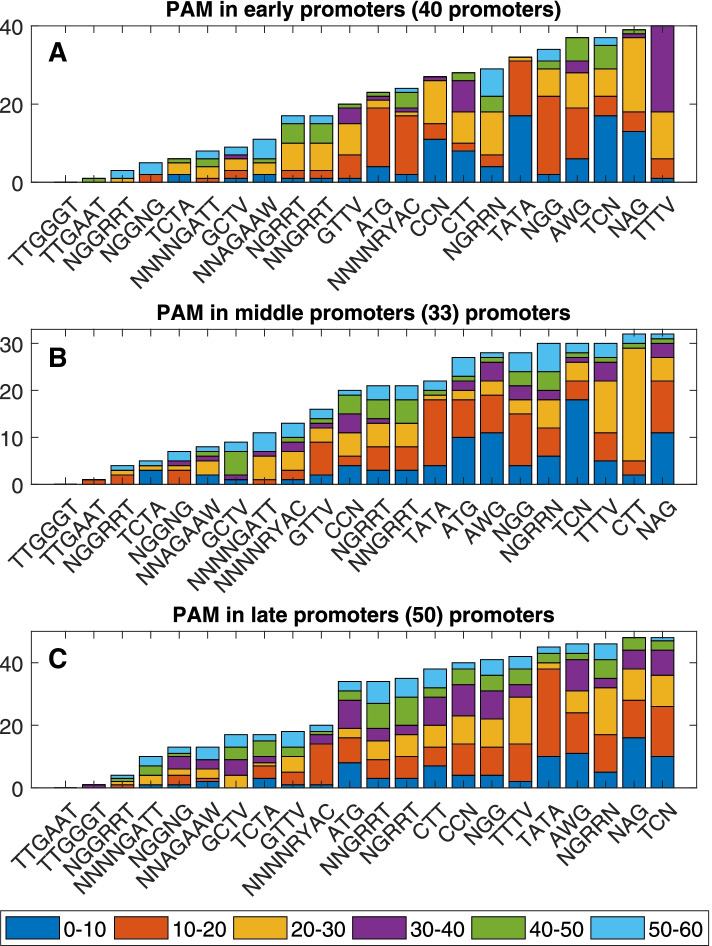
PAM’s motifs distribution in each of the three types of promoters: 40 early (**A**), 33 middle (**B**), and 50 late (**C**). Sixty bases were scanned for each promoter upstream of the transcription start site (which is given the coordinate 0). The 60 bases of a promoter were categorized into six segments represented by stacked bars of different colors. Y axis represents PAM’s count number

Phage protospacer adjacent motifs, PAMs, are not only required for bacterial CRISPR-Cas system to cut, but they can have a significant effect on the efficiency of gene expression by their presence within DNA sequence of phage promoters. MATLAB was used for analyzing T4 phage genome to determine the distribution pattern of the most known protospacer adjacent motif, PAMs (Table 1). The repetitive feature was determined for the different PAMs within promoter regions that span a length of 60 bases. The distribution pattern of PAMs was analyzed in accordance with their length and promoter elements (-10 position, -35 position). Figure 2 shows a preference for a specific PAMs in each type of promoter. The most favorable PAMs are TTTV and NAG for early and middle promoters respectively, while late promoters are rich with TCN and NAG.

For early promoters, at position 20-30, the highest repetitive PAM is NAG, while 10-20 position contains the maximum frequency of NGG PAMs. A specific feature of middle promoters is having CTT in high frequency at position 20-30. Calculations show that the highest count of TATA are in 0-10 for early promoters, and 10-20 for both middle and late promoters.

The examined PAM sequences are located mostly at -10 position whereas at -35 there was no or little detection. Promoters of all phage’s genes contain highly conservative sequences of 5’-TATAAT-3’ around the -10 region, resembling the consensus sequence TATTA in promoter. These results provide novel insights into the location and the subsequent identification of the role of PAMs as transcriptional regulatory elements.

The following PAMs were found to have high sequence hit in T4 phage promoters, but they had a very different location with each hit, ‘TCN’, ‘NGRRN’ and ‘AWG’.

The *P*-value we got from performing the MWW test was extremely small (rejected null hypothesis), showing that there is a significant difference between the two distributions, and that for any two observations selected each from one of the groups, the probability of one being greater than the other is not equal to the probability of it being smaller than the other. This means the following, CRISPR-Cas does not select its PAMs randomly, and that the fact that a sequence is a regulatory sequence which is part of a promoter, affects this decision.

For genes essentiality, surprisingly, most of the targeted genes by most of the PAMs were non-essential genes. We explained this by the fact that only 62 genes are essential in the T4 phage out of 292 total genes. We found that the genes targeted by GCTV, TTGAAT and TTGGGT PAMs have a higher chance of being essential than the rest of PAMs (Table 2). This can be used to explain theoretically CRISPR selection for some PAMs based on essentiality of the genes.

**Table 2:** Fraction of targeted essential genes per PAM (number of essential genes where the PAM count was detected as an outlier). Only significant genes were used in the calculation of the fractions

PAM	Fraction of targeted essential genes
ATG	0.125
AWG	0.2
CCN	0
CTT	0
GCTV	0.4
GTTV	0.142857143
NAG	0
NGG	0
NGGNG	0.1
NGGRRT	0
NGRRN	0
NGRRT	0
NNAGAAW	0
NNGRRT	0
NNNNGATT	0
NNNNRYAC	0
TATA	0
TCN	0
TCTA	0.2
TTGAAT	0.255813953
TTGGGT	0.28
TTTV	0

Final analysis was done to correlate the selectivity of PAM sequences to a class of translated amino acids with certain property. Amino acids are classified based on the R group into polar and non-polar side chains. Many studies rely on the sequence data base to build a relationship between the hydrophobicity of amino acids and their localization on surface protein. Generally, the non-polar side chains of residues (e.g., V, L, I, M, F, A, Y, W) are in the inner (core) structure while the polar residues (e.g., K, R, S) are found on the outer exposed surface allowing the proper thermodynamics of protein-water interaction [40].

This analysis was made based on the assumption that PAM sequences are coded in different triplets (Table 3) and each codon is translated to one or more amino acids. Any of the first two nucleotides (bases) in the four PAM nucleotides was set as a start base in the triplet codon, and this was made based on the occurrence of different open reading frames for transcription. The same rule was applied to five or more PAM nucleotides for the generation of all possible triplets from the same PAM sequence.

**Table 3:** Shows all PAMs as triplet DNA codons and the possible amino acids generated from each codon

PAM	Amino acids
ATG	M
AWG	K M
CCN	P
CTT	L
GCTV	L A
GTTV	V L F
NAG	K Q E
NGG	R G W
NGGNG	R E A G V W
NGGRRT	N R S E D G W
NGRRN	K N R S E D G W
NGRRT	N R S E D G W
NNAGAAW	K N T R I Q P L E A G V S
NNGRRT	K N T R S M Q P L E D A G V W
NGATT	R I D G
NRYAC	N T S I H R D A G V Y C
TATA	I Y
TCN	S
TCTA	L S
TTGAAT	N E L
TTGGGT	G W L
TTTV	L F

**Fig. 3:**
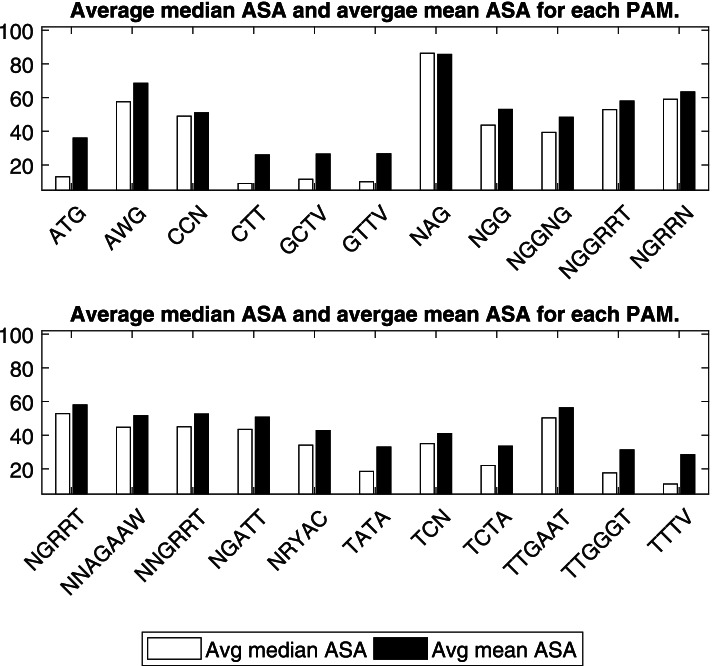
Mean ASA (average surface accessibility) values, averaged across the different possible codons per PAM

Based on the results that show the distribution of PAMs throughout amino acid coding sequences (Fig. 3), there is no strong correlation for the distribution of polar residues on protein surfaces. This is similar to the outcome of different mechanistic studies proved that there are many coordinates to be included in calculating the accessible surface area of protein residues [39, 41].

**Fig. 4:**
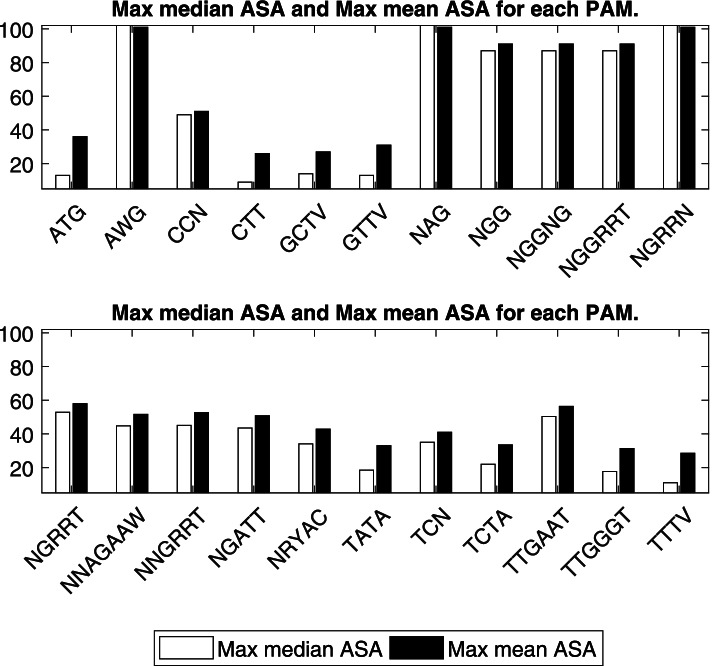
Maximum mean ASA (average surface accessibility) values, across the different possible codons per PAM

All the PAMs had ASA values for their codons (Fig. 4) that is not sufficiently high to justify their selection on the ground of surface exposure of the amino acids (based on the translated codons obtained from the PAMs). However, when we look at the maximum ASA per PAM that can be obtained, and not the average value, we might be able to justify CRISPR selection for some of these PAMs using surface exposure. For example, AWG (which matches the codons ATG, AAG), and NAG (AAG, TAG, CAG, GAG), despite being short and have a small codons list, they have a high ASA value each, thus one possible explanation for their selection is due to their polar side chains and therefore their location on protein surface.

Moreover, it is well proven that surface residues of a protein vary in their function, for instance the ones that mediate interaction with other proteins are called the interface surface residues [42]. These residues usually have high ASA values. Our theoretical hypothesis is closely linked to the comparative analytical study that was done by Caffrey [38] to find out the conservation scores of interface surface residues. It was found that interface residues are more conservative that the rest of the exposed residues.

Based on the importance of interface surface residues in protein activity, we can conclude that the driving force for PAM’s selection in CRISPR types is the interface surface residues, and their polar side chains, these factors make them preferable to function in protein-protein interaction.

## Conclusion

Aside from their significant role in bacterial adaptive immune system, this study related PAMs positions to their function in transcription regulation. This work provides the first examination, to our knowledge, of the coordinates and localization of PAMs motifs in phage genomes. Distribution pattern of different PAMs was estimated and identified in this study. It was found that highly conserved regulatory sequences, might have the strongest explanation for PAM selection in some CRISPR types.

The following CRISPR types were found to target early promoters: V-A with ‘TTTV’ PAM, I-C with ‘NAG’, I-A with ‘TCN’, I-E with ‘AWG’, II-A with ‘NGG’ and V-A with ‘TATA’. While the following types target middle promoters: I-C with ‘NAG’, I-E with ‘CTT’, V-A with ‘TTTV’, I-A with ‘TCN’, II-A with ‘NGRRN’ and II-A with ‘NGG’. Finally, the following types target the late promoters: I-A with ‘TCN’, I-C with ‘NAG’, II-A with ‘NGRRN’, I-E with ‘AWG’, V-A with ‘TATA’ and ‘TTTV’. On the other hand, coding regions do not have the same level of explanatory power for PAM selection.

## Supplementary Information

**Additional file 1.**

## Data Availability

The T4 genome sequence used for analysis is available in the NCBI Entrez Genome site (https://www.ncbi.nlm.nih.gov/nuccore/NC_000866.4/), The NCBI Reference Sequence: NC_000866.4. The Fasta file for T4 nucleotide sequence is uploaded with the supplementary files. The software code and the output datasets supporting the conclusions of this article are included within the article and its additional files.
