# Supplementary material for: Bio-informatic analysis of CRISPR protospacer adjacent motifs (PAMs) in T4 genome
Source: BMC Genom Data. 2022 Jun 2;23:40. doi: 10.1186/s12863-022-01056-8 (PMC9161530; doi:10.1186/s12863-022-01056-8)
Supplement: Supplementary file 1 — Additional file 1. [file 12863_2022_1056_MOESM1_ESM.zip › ChangePAMs2ATCG.pdf]

```
function [PAMs] = ChangePAMs2ATCG(PAM)
%CHANGEPAMS2ATCG Summary of this function goes here
%   Detailed explanation goes here
    PAMsResults = strings(1);
    matchList = {'U', {'T'};
                 'R', {'A', 'G'};
                 'Y', {'T', 'C'};
                 'K', {'G', 'T'};
                 'M', {'A', 'C'};
                 'S', {'C', 'G'};
                 'W', {'A', 'T'};
                 'B', {'T', 'C', 'G'};
                 'D', {'A', 'T', 'G'};
                 'H', {'A', 'T', 'C'};
                 'V', {'A', 'C', 'G'};
                 'N', {'A', 'T', 'C', 'G'};};

    findVector = [matchList{1:12, 1}];
    for i=1:strlength(PAM)
        letterIndex = strfind(findVector, PAM.extractBetween(i, i));
        if (isempty(letterIndex))
            for j=1:length(PAMsResults)
                PAMsResults(j) = insertAfter(PAMsResults(j), strlength(PAMsResults(j)), ↙
PAM.extractBetween(i, i));
            end
            continue;
        end
        numberOfCharacters = length(matchList{letterIndex, 2});

        originalLength = length(PAMsResults);
        PAMsResults = repmat(PAMsResults, 1, numberOfCharacters);
        for j=1:length(PAMsResults)
            PAMsResults(j) = insertAfter(PAMsResults(j), strlength(PAMsResults(j)), ↙
matchList{12, 2}{ceil(j/originalLength)});
        end
    end
    PAMs = PAMsResults;
end
```
