# Supplementary material for: Bio-informatic analysis of CRISPR protospacer adjacent motifs (PAMs) in T4 genome
Source: BMC Genom Data. 2022 Jun 2;23:40. doi: 10.1186/s12863-022-01056-8 (PMC9161530; doi:10.1186/s12863-022-01056-8)
Supplement: Supplementary file 1 — Additional file 1. [file 12863_2022_1056_MOESM1_ESM.zip › compareNucleotidesSeq.pdf]

```
function match = compareNucleotidesSeq(seq1,seq2)
    seqChar = convertStringsToChars(seq2);
    matchList = {'A', {'A', 'R', 'M', 'W', 'D', 'H', 'V', 'N'};
                 'T', {'T', 'Y', 'K', 'W', 'B', 'D', 'H', 'N'};
                 'C', {'C', 'Y', 'M', 'S', 'B', 'H', 'V', 'N'};
                 'G', {'G', 'R', 'K', 'S', 'B', 'D', 'V', 'N'};
                 'U', {'T', 'Y', 'K', 'W', 'B', 'D', 'H', 'N'};
                 'R', {'R', 'A', 'G', 'M', 'W', 'D', 'H', 'V', 'N', 'K', 'S', 'B'};
                 'Y', {'Y', 'T', 'C', 'K', 'W', 'B', 'D', 'H', 'N', 'M', 'S', 'V'};
                 'K', {'K', 'G', 'T', 'R', 'S', 'B', 'D', 'V', 'N', 'Y', 'W', 'H'};
                 'M', {'M', 'A', 'C', 'R', 'W', 'D', 'H', 'V', 'N', 'Y', 'S', 'B'};
                 'S', {'S', 'C', 'G', 'Y', 'M', 'B', 'H', 'V', 'N', 'R', 'K', 'D'};
                 'W', {'W', 'A', 'T', 'R', 'M', 'D', 'H', 'V', 'N', 'Y', 'K', 'B'};
                 'B', {'B', 'T', 'C', 'G', 'Y', 'K', 'W', 'D', 'H', 'N', 'M', 'S', 'V', ↙
'R'};
                 'D', {'D', 'A', 'T', 'G', 'R', 'M', 'W', 'H', 'V', 'N', 'Y', 'K', 'B', ↙
'S'};
                 'H', {'H', 'A', 'T', 'C', 'R', 'M', 'W', 'D', 'V', 'N', 'Y', 'K', 'B', ↙
'S'};
                 'V', {'V', 'A', 'C', 'G', 'R', 'M', 'W', 'D', 'H', 'N', 'Y', 'S', 'B', ↙
'K'};
                 'N', {'N', 'A', 'T', 'C', 'G', 'U', 'R', 'Y', 'K', 'M', 'S', 'W', 'B', ↙
'D', 'H', 'V'};};
      n = strlength(seq1);
      if (n ~= strlength(seqChar))
          match = false;
          return;
      end

      seq1 = convertStringsToChars(seq1);
      for i=1:n
          for j=1:16
             if (strcmp(matchList{j, 1}, seq1(i)))
                currCharMatched = false;
                for k=1:length(matchList{j, 2})
                    if (strcmp(matchList{j, 2}{k},seqChar(i)))
                        currCharMatched = true;
                        break;
                    end
                end
                if (~currCharMatched)
                   match = false;
                   return;
                end
                break; %no need to continue looping we found the row
             end
          end
      end

      match = true;
      return;
end
```
