# Supplementary material for: Bio-informatic analysis of CRISPR protospacer adjacent motifs (PAMs) in T4 genome
Source: BMC Genom Data. 2022 Jun 2;23:40. doi: 10.1186/s12863-022-01056-8 (PMC9161530; doi:10.1186/s12863-022-01056-8)
Supplement: Supplementary file 1 — Additional file 1. [file 12863_2022_1056_MOESM1_ESM.zip › FindPAMsInPhageDNA.pdf]

```
close all;

%data
%**********************************************************
phageSeq = erase(fileread('T4 sequence.fasta'), newline);
geneStartEnd = [12, 2189; 2200, 2403; 2458, 2802; 2853, 2990; 2971, 3351; 3654, 3767; ↙
3778, 5328; 5398, 5595; 5702, 5839; 5842, 6267; 6267, 6482; 6653, 7141; 7291, 7650;
        7661, 8161; 8225, 8908; 8908, 9150; 9143, 9388; 9410, 10729; 10726, 11037; ↙
11039, 11785; 11908, 12510; 12507, 13130; 13198, 13380; 13389, 13859; 13852, 14016; ↙
14013, 14216;
        14191, 14676; 14685, 15026; 15026, 15232; 15331, 15573; 15606, 16280; 16270, ↙
16785; 16846, 17625; 17935, 18963; 18966, 19130; 19132, 19758; 19758, 20051; 20112, ↙
20369;
        20371, 20553; 20612, 22039; 22049, 22393; 22386, 23561; 23570, 24235; 24400, ↙
25455; 25479, 26219; 26373, 26624; 26636, 27013; 27197, 29893; 29972, 30340; 30342, ↙
30905;
        30907, 31866; 31917, 32603; 32659, 33048; 33058, 33246; 33302, 34984; 34981, ↙
35187; 35168, 35431; 35428, 36447; 36444, 36584; 36624, 37826; 37885, 38679; 38731, ↙
38922;
        38907, 39110; 39079, 39396; 39398, 39616; 39600, 40157; 40193, 40456; 40459, ↙
40785; 40799, 41038; 41039, 41170; 41178, 41471; 41464, 41646; 41805, 42113; 42116, ↙
42328;
        42446, 42916; 42906, 43538; 43535, 44814; 44836, 45612; 45848, 46385; 46382, ↙
46699; 46382, 46855; 46897, 47382; 47366, 47521; 47506, 47826; 47823, 48131; 48128, ↙
48391;
        48393, 48635; 48622, 48936; 48933, 49859; 49914, 50915; 50973, 51995; 52066, ↙
52893; 52901, 53302; 53358, 53885; 53946, 54248; 54343, 55320; 55435, 56445; 56429, ↙
57208;
        57283, 57828; 57828, 57932; 58049, 58165; 58155, 58349; 58352, 58534; 58534, ↙
58722; 58819, 59205; 59202, 59495; 59508, 59720; 59763, 60344; 60346, 60534; 60531, ↙
60716;
        60713, 60925; 60922, 61389; 61386, 61733; 61726, 62271; 62279, 62740; 62800, ↙
63078; 63078, 63344; 63381, 63557; 63557, 63919; 63927, 64256; 64253, 64912; 64939, ↙
65355;
        65416, 65718; 65834, 66415; 66503, 66997; 67035, 67490; 67652, 67960; 67957, ↙
68319; 68301, 68693; 68662, 69270; 69312, 69905; 69968, 70303; 70360, 70623; 70908, ↙
71046;
        71053, 71171; 71173, 71247; 71253, 71918; 71960, 72033; 72035, 72110; 72118, ↙
72204; 72203, 72280; 72291, 72364; 72369, 72456; 72456, 72530; 72628, 72915; 72918, ↙
73328;
        73329, 73514; 73591, 73878; 73952, 74410; 74407, 74649; 74649, 75374; 75424, ↙
75954; 76061, 76885; 76885, 77337; 77385, 77975; 77959, 79686; 77981, 78118; 79085, ↙
79237;
        79721, 80215; 80196, 80618; 80621, 80791; 80779, 81072; 81081, 83063; 83060, ↙
86158; 86151, 87155; 87219, 88085; 88085, 89893; 89893, 90552; 90549, 92132; 92129, ↙
93592;
        93624, 94553; 94555, 95325; 95367, 96185; 96194, 96688; 96672, 98504; 96933, ↙
98504; 96987, 98504; 97254, 98504; 98536, 100515; 100632, 101123; 101207, 102781; ↙
102781, 103023;
        103023, 103448; 103448, 104086; 103514, 104086; 104117, 104926; 104945, 106510; ↙
106561, 107232; 107323, 108606; 108636, 109640; 109650, 109928; 109915, 110085; 110187, ↙
111317;
        111327, 112007; 112057, 112674; 112677, 114440; 114496, 114663; 114690, 114914; ↙
114914, 115327; 115404, 115802; 115802, 116089; 115802, 116428; 116479, 117228; 117228, ↙
118403;
        118348, 118881; 118878, 120650; 120659, 121753; 121753, 122715; 122742, 123032; ↙
```

```
123065, 123268; 123265, 123450; 123454, 125502; 125560, 125748; 125745, 127208; 127205, ↙
127474;
        127474, 128310; 128402, 128629; 128307, 128765; 128758, 128964; 128961, 129158; ↙
129158, 129445; 129487, 129852; 129921, 130253; 130364, 130540; 130785, 131033; 131181, ↙
131516;
        131573, 131881; 131882, 132118; 132118, 132699; 132696, 133034; 133031, 133261; ↙
133334, 133609; 133612, 133812; 133805, 134032; 134002, 134907; 134908, 135135; 135132, ↙
135431;
        135428, 135781; 135772, 136275; 136340, 137464; 137517, 137951; 137955, 138457; ↙
138593, 138886; 139056, 139719; 139716, 139967; 139991, 140416; 140416, 142680; 142671, ↙
142997;
        142951, 143214; 143235, 143546; 143694, 144431; 144564, 145112; 145109, 145690; ↙
145762, 146004; 146143, 146529; 146575, 146802; 146948, 147853; 147909, 148541;
        148543, 149196; 149193, 149531; 149509, 149778; 149787, 150704; 150809, 154678; ↙
154687, 155805; 155868, 156533; 156542, 159622; 159649, 160200; 160221, 160877; 160878, ↙
161150;
        161163, 161315; 161312, 161590; 161674, 161805; 161876, 162172; 162172, 162630; ↙
162627, 162833; 162967, 163602; 163730, 163879; 163876, 165204; 165209, 165349; 165342, ↙
165497;
        165505, 165585; 165585, 166040; 166101, 166316; 166325, 166435; 166432, 166554; ↙
166548, 166628; 166636, 166764; 166815, 166913; 166910, 166996; 167103, 167660; 167743, ↙
167937;
        167965, 168903];

genesLength = geneStartEnd(:, 2)-geneStartEnd(:,1);
genes = ["rIIA";"rIIA.1";"60";"60";"60.1";"mobA";"39";"39.1";"39.2";"goF = comC?";"cef ↙
= mb";"motB";"motB.1";"motB.2";"dexA";"dexA.1";"dexA.2";"dda";"dda.1";"srd";"modA";" ↙
modB";"modA.2";"modA.3";"modA.4";"srh";"mrh";"mrh.1";"mrh.2";"soc";"segF = 69";"56";" ↙
dam";"61 = 58";"61.1";"61.2";"sp = rV";"61.4";"dmd = 61.5";"41";"40";"uvsX";"segA";"?- ↙
gt";"42";"imm";"imm.1";"43";"regA";"62";"44";"45";"rpbA";"45.2";"46";"46.1";"46.2";" ↙
47";"47.1";"?-gt";"mobB";"?-gt.2";"?-gt.3";"?-gt.4";"?-gt.5";"55";"55.1";"55.2";" ↙
55.3";"55.4";"55.5";"55.6";"nrdH";"55.8";"nrdG";"mobC";"nrdD+";"I-TevII";"nrdD+";" ↙
49'";"49";"pin";"49.1";"49.2";"49.3";"nrdC";"nrdC.1";"nrdC.2";"nrdC.3";"nrdC.4";"nrdC. ↙
5";"nrdC.6";"nrdC.7";"nrdC.8";"nrdC.9";"nrdC.10";"nrdC.11";"mobD";"mobD.1";"mobD.2";" ↙
mobD.2a";"mobD.3";"mobD.4";"mobD.5";"rI.-1";"rI";"rI.1";"tk";"tk.1";"tk.2";"tk.3";"tk. ↙
4";"vs";"vs.1";"regB";"vs.3";"vs.4";"vs.5";"vs.6";"vs.7";"vs.8";"denV";"ipII";"ipIII";" ↙
e";"nudE = e.1";"e.2";"e.3";"e.4";"e.5";"e.6";"e.7";"e.8";"rnaC";"rnaD";"tRNAR";" ↙
segB";"tRNAI";"tRNAT";"tRNAS";"tRNAP";"tRNAG";"tRNAL";"tRNAE";"tRNA.2";"tRNA.3";"tRNA. ↙
4";"ipI";"57B";"57A";"1";"3";"2 = 64";"4 = 50 = 65";"53";"5";"repEB";"repEA";"5.1";" ↙
segC";"5.3";"5.4";"6";"7";"8";"9";"10";"11";"12";"wac";"13";"14";"15";"16";"17";" ↙
17'A";"17'B";"17""";"18";"19";"20";"67";"68";"21";"21'";"22";"23";"segD";"24";"rnlB = ↙
24.1";"24.2";"24.3";"hoc";"inh";"segE";"uvsW";"uvsY.-2";"uvsY.-1";"uvsY";"25";"26'";" ↙
26";"51";"27";"28";"29";"48";"54";"alt.-3";"alt.-2";"alt.-1";"alt";"alt.1";"30";" ↙
30.1";"30.2";"30.3'";"30.3";"30.4";"30.5";"30.6";"30.7";"30.8";"30.9";"rIII";"31";" ↙
31.1";"31.2";"cd";"cd.1";"cd.2";"cd.3";"cd.4";"cd.5";"pseT";"pseT.1";"pseT.2";"pseT. ↙
3";"alc";"rnlA = 63";"denA";"nrdB+";"I-TevIII";"nrdB+";"nrdB.1";"mobE";"nrdA";"nrdA. ↙
1";"nrdA.2";"td+";"I-TevI";"td+";"frd";"frd.1";"frd.2";"frd.3";"32";"segG = 32.1";" ↙
59";"33";"dsbA";"rnh";"34";"35";"36";"37";"38";"t";"asiA";"asiA.1";"arn";"arn.1";"arn. ↙
2";"arn.3";"arn.4";"motA";"motA.1";"52";"52.1";"ac";"stp";"ndd";"ndd.1";"ndd.2";"ndd. ↙
2a";"ndd.3";"ndd.4";"ndd.5";"ndd.6";"denB";"denB.1";"rIIB"];
direction = ["-";"-";"-";"-";"-";"-";"-";"-";"-";"-";"-";"-";"-";"-";"-";"-";"-";"-";"- ↙
";"-";"-";"-";"-";"-";"-";"-";"-";"-";"-";"-";"-";"-";"-";"-";"-";"-";"-";"-";"-";"-";" ↙
-";"-";"-";"-";"-";"-";"-";"-";"-";"-";"-";"-";"-";"-";"-";"-";"-";"-";"-";"-";"-";"- ↙
";"-";"-";"-";"-";"-";"-";"-";"-";"-";"-";"-";"-";"-";"-";"-";"-";"-";"-";"-";"-";"-";" ↙
-";"-";"-";"-";"-";"-";"-";"-";"-";"-";"-";"-";"-";"-";"-";"-";"-";"-";"-";"-";"-";"- ↙
";"-";"-";"-";"-";"-";"-";"-";"-";"-";"-";"-";"-";"-";"-";"-";"-";"-";"-";"-";"-";"-";" ↙
```

```
-";"-";"-";"-";"-";"-";"-";"-";"-";"-";"-";"-";"-";"-";"-";"-";"-";"-";"-";"-";"-";"- ↙
";"-";"-";"-";"-";"-";"-";"+";"+";"-";"-";"+";"+";"+";"+";"+";"+";"+";"+";"+";"+";"+";" ↙
+";"+";"+";"+";"+";"+";"+";"+";"+";"+";"+";"+";"+";"+";"+";"+";"+";"+";"-";"+";"-";"- ↙
";"-";"-";"-";"+";"+";"-";"-";"-";"-";"-";"-";"+";"+";"+";"+";"+";"+";"-";"-";"-";"-";" ↙
-";"-";"-";"-";"-";"-";"-";"-";"-";"-";"-";"-";"-";"-";"-";"-";"-";"-";"-";"-";"-";"- ↙
";"-";"-";"-";"-";"-";"-";"-";"-";"-";"-";"-";"-";"-";"-";"-";"-";"-";"-";"-";"-";" ↙
-";"-";"-";"-";"-";"-";"-";"+";"+";"+";"+";"+";"+";"-";"-";"-";"-";"-";"-";"-";"-";"- ↙
";"-";"-";"-";"-";"-";"-";"-";"-";"-";"-";"-";"-";"-";"-";"-"];
genesPromoters = ["Pm";"Pe (1.4 -3.9)";"Pe (3.6)";"Pe (3.6)";"Pe (3.6)";"Pe (3.6)";" ↙
Pm";"Pe (5.9-7.3)";"Pe (5.9-7.3)";"Pe (5.9-7.3)";"Pe (5.9-7.3)";"Pe (5.9-7.3)";"Pe↙
(8.1)";"Pe (8.1)";"Pe (11.5)";"Pe (11.5)";"Pe (11.5)";"Pe (11.5)";"Pe (11.5)";"Pe ↙
(11.5)";"Pe (12.8)";"Pe (12.8)";"Pe (15.0)";"Pe (15.0)";"Pe (15.0)";"Pe (15.0)";"Pe ↙
(15.0)";"Pe (15.0)";"Pe (15.0)";"Pl";"Pl";"Pm";"Pm";"Pm";"Pe (19.8)";"Pe (19.8)";"Pe ↙
(19.8)";"Pe (20.3)";"Pe (20.3)";"Pm";"Pm";"Pm";"Pm";"Pm";"Pm";"Pe (26.4)";"Pe (26.4)";" ↙
Pm";"Pm";"Pm";"Pm";"Pm";"Pm";"Pm";"Pm";"Pe (35.3)";"Pe (35.3)";"Pm";"Pm";"Pm";"Pm";" ↙
Pm";"Pm";"Pm";"Pm";"Pm";"Pe (40.4)";"Pe (40.4)";"Pe (40.4)";"Pe (40.4)";"Pe (41.0)";"Pe ↙
(41.0)";"Pm";"Pm";"Pm";"Pl";"Pl";"Pl";"Pm";"Pl";"Pl";"Pe (46.7)";"Pe (50.0)";"Pe ↙
(50.0)";"Pe (50.0)";"Pe (50.0)";"Pe (50.0)";"Pe (50.0)";"Pe (50.0)";"Pe (50.0)";"Pe ↙
(54.0)";"Pe (54.0)";"Pe (54.0)";"Pe (54.0)";"Pe (54.4)";"Pe (54.4)";"Pe";"Pe";"Pe";" ↙
Pe";"Pe (57.9)";"Pe (57.9)";"Pe (57.9)";"Pe (57.9)";"Pl";"Pl";"Pl";"Pe (62.2)";"Pe ↙
(62.2)";"Pe (62.2)";"Pe (62.2)";"Pe (62.2)";"Pe (62.2)";"Pe (62.2)";"Pe (62.2)";"Pe ↙
(64.6)";"Pe (64.6)";"Pe (64.6)";"Pe (64.6)";"Pe (64.6)";"Pe (64.6)";"Pe (64.6)";"Pe ↙
(65.0)";"Pe";"Pl";"Pe (69.9)";"Pe (69.9)";"Pe (69.9)";"Pe (69.9)";"Pe (69.9)";"Pe ↙
(69.9)";"Pe (69.4)";"Pe (69.8)";"Pm";"Pm";"Pm";"Pm";"Pm";"Pm";"Pm";"Pm";"Pm";"Pm";" ↙
Pm";"Pe (72.6)";"Pe (72.6)";"Pe (72.6)";"Pe (73.0)";"Pm";"Pm";"Pm";"Pl";"Pl";"Pl";" ↙
Pl";"Pl";"Pe";"Pe";"Pl";"Pl";"Pl";"Pl";"Pl";"Pl";"Pl";"Pl";"Pl";"Pl";"Pl";"Pl";"Pl";" ↙
Pl";"Pl";"Pl";"Pl";"Pl";"Pl";"Pl";"Pl";"Pl";"Pl";"Pl";"Pl";"Pl";"Pl";"Pl";"Pl";"Pl";" ↙
Pl";"Pl";"Pl";"Pl";"Pl";"Pl";"Pl";"Pl";"Pl";"Pm";"Pm";"Pl";"Pl";"Pl";"Pl";"Pl";"Pl";" ↙
Pl";"Pl";"Pl";"Pe";"Pl";"Pl";"Pl";"Pe (128.2)";"Pe (128.2)";"Pe (128.2)";"Pe (128.2)";" ↙
Pe (128.2)";"Pe (128.2)";"Pe (128.2)";"Pe (128.2)";"Pe (128.2)";"Pe (128.2)";"Pe ↙
(128.6)";"Pl";"Pl";"Pe (131.7)";"Pe (131.7)";"Pe (131.7)";"Pe (131.7)";"Pe (131.7)";"Pe ↙
(131.7)";"Pe (134.4)";"Pe (134.4)";"Pe (134.4)";"Pe (134.4)";"Pe (134.4)";"Pe ↙
(134.4)";"Pe (134.4)";"Pe (134.4)";"Pl";"Pl";"Pl";"Pl";"Pm";"Pm";"Pm";"Pm";"Pl";"Pl";" ↙
Pl";"Pl";"Pm";"Pe (144.6)";"Pe (144.6)";"Pe (144.6)";"Pe (144.6)";"Pl";"Pm";"Pm";"Pm";" ↙
Pm";"Pe (148.6)";"Pl";"Pl";"Pl";"Pl";"Pl";"Pl";"Pe (158.7)";"Pe (161.1)";"Pe (161.1)";" ↙
Pe (161.1)";"Pe (161.1)";"Pe (161.1)";"Pe (161.1)";"Pe (161.1)";"Pe (164.2)";"Pe ↙
(164.2)";"Pe (164.2)";"Pe (164.2)";"Pe (164.2)";"Pe (164.2)";"Pe (164.2)";"Pe ↙
(164.2)";"Pe (164.2)";"Pe (164.2)";"Pe (164.2)";"Pe (164.5)";"Pe (164.5)";"Pm";"Pm";" ↙
Pm"];
essential = [0; 0; 1; 1; 1; 0; 1; 0; 0; 0; 0; 0; 0; 0; 0; 0; 0; 0; 0; 0; 0; 0; 0; 0; 0; ↙
0; 0; 0; 0; 0; 0; 1; 0; 0; 0; 0; 0; 0;
            0; 1; 0; 0; 0; 0; 1; 0; 0; 1; 0; 1; 1; 1; 0; 0; 1; 0; 0; 1; 0; 0; 0; 0; 0; ↙
0; 0; 1; 0; 0; 0; 0; 0; 0; 0; 0; 0; 0; 0; 0; 0; 1; 1; 0; 0;
            0; 0; 0; 0; 0; 0; 0; 0; 0; 0; 0; 0; 0; 0; 0; 0; 0; 0; 0; 0; 0; 0; 0; 0; 0; ↙
0; 0; 0; 0; 0; 0; 0; 0; 0; 0; 0; 0; 0; 0; 0; 0; 1; 0; 0; 0;
            0; 0; 0; 0; 0; 0; 0; 0; 0; 0; 0; 0; 0; 0; 0; 0; 0; 0; 0; 0; 0; 1; 1; 1; 1; ↙
1; 1; 1; 0; 0; 0; 0; 0; 0; 1; 1; 1; 1; 1; 1; 1; 0; 1; 1; 1;
            1; 1; 0; 0; 0; 1; 1; 1; 1; 1; 1; 0; 1; 1; 0; 1; 0; 0; 0; 0; 0; 0; 0; 0; 0; ↙
0; 1; 0; 1; 1; 1; 1; 1; 1; 1; 0; 0; 0; 0; 0; 1; 0; 0; 0; 0;
            0; 0; 0; 0; 0; 0; 0; 1; 0; 0; 0; 0; 0; 0; 0; 0; 0; 0; 0; 0; 0; 0; 0; 0; 0; ↙
0; 0; 0; 0; 0; 0; 0; 0; 0; 0; 0; 0; 0; 0; 1; 0; 1; 1; 0; 0;
            1; 1; 1; 1; 1; 1; 1; 0; 0; 0; 0; 0; 0; 0; 0; 1; 0; 0; 0; 0; 0; 0; 0; 0; 0; ↙
0; 0; 0; 0];

promoters = ["Pm";"Pm";"Pm";"Pe (1.4 -3.9)";"Pe (3.6)";"Pm";"Pe (5.9 -7.3)";"Pe (8.1)";"↙
Pe (11.5)";"Pe (12.8)";"Pe (15.0)";"Pl";"Pl";"Pl";"Pm";"Pm";"Pe (19.8)";"Pe (20.3)";" ↙
```

```
Pm";"Pm";"Pm";"Pe (26.4)";"Pm";"Pm";"Pm";"Pm";"Pe (35.3)";"Pm";"Pm";"Pm";"Pe (40.4)";" ↙
Pe (41.0)";"Pm";"Pm";"Pl";"Pm";"Pl";"pin";"Pe (46.7)";"Pe (50.0)";"Pe (54.0)";"Pe ↙
(54.4)";"Pe";"Pe (57.9)";"Pl";"Pe (62.2)";"Pe (64.6)";"Pe (65.0)";"Pe";"Pl";"Pl";"Pl";" ↙
Pe (69.9)";"Pe (69.4)";"Pe (69.8)";"Pm";"Pl";"Pe (72.6)";"Pe (73.0)";"Pm";"Pl";"Pm";" ↙
Pl";"Pl";"Pl";"Pl";"Pe";"Pl";"Pl";"Pl";"Pl";"Pl";"Pl";"Pl";"Pl";"Pl";"Pl";"Pl";"Pl";" ↙
Pl";"Pl";"Pl";"Pl";"Pl";"Pl";"Pl";"Pl";"Pl";"Pm";"Pl";"Pl";"Pl";"Pl";"Pe";"Pl";"Pm";" ↙
Pm";"Pe (128.2)";"Pe (128.6)";"Pl";"Pm";"Pe (131.7)";"pseT";"pseT.1";"pseT.2";"pseT. ↙
3";"Pe (134.4)";"Pl";"Pl";"Pm";"Pm";"Pm";"Pl";"Pm";"Pe (144.6)";"Pl";"Pm";"Pm";"Pe ↙
(148.6)";"Pl";"Pm";"Pl";"Pl";"Pl";"Pe (158.7)";"Pe (161.1)";"Pe (164.2)";"Pe (164.5)"];
promotersDirection = ["-";"-";"-";"-";"-";"-";"-";"-";"-";"-";"-";"-";"-";"-";"-";"-";" ↙
-";"-";"-";"-";"-";"-";"-";"-";"-";"-";"-";"-";"-";"-";"-";"-";"-";"-";"-";"-";"-";"- ↙
";"-";"-";"-";"-";"-";"-";"-";"-";"-";"-";"-";"-";"-";"-";"-";"-";"-";"-";"-";"-";"-";" ↙
-";"-";"-";"-";"+";"+";"+";"-";"+";"+";"+";"+";"+";"+";"+";"+";"+";"+";"+";"+";"+";" ↙
+";"+";"+";"+";"-";"+";"+";"-";"-";"-";"-";"-";"+";"-";"-";"";"";"-";"-";"-";"";"-";"- ↙
";"-";"-";"-";"-";"+";"-";"-";"-";"-";"-";"-";"-";"-";"-";"-";"-";"+";"-";"+";"+";"+";" ↙
-";"-";"-";"-"];
promotersLoc = [123;377;2263;2422;3847;5349;7179;8182;11815;13150;15252;15597;16162; ↙
16359;16813;19122;20073;20576;23752;24460;26317;27044;29931;32626;33257;35014;35662; ↙
36576;38681;40180;41225;41670;42805;43023;45625;46441;46879;47382;47416;50937;53907; ↙
55348;57954;58744;59740;62761;65378;65763;66462;67005;67018;67234;69931;70323;70660; ↙
72593;72863;73536;73903;74877;74999;75393;77358;77362;77381;77491;79405;79799;85812; ↙
87200;87885;95337;96153;96913;98513;100564;100623;101184;102539;103146;104095;104787; ↙
104816;107301;112029;112034;112588;114681;115371;116412;116436;116444;116467;123057; ↙
125525;127234;128355;129883;130274;131167;131540;133295;134907;135135;135431;135781; ↙
136300;136889;138933;138939;139878;142725;144449;145142;146839;147998;148057;149873; ↙
150727;150780;153011;155850;156369;160209;161175;163637;166771;167050];

PAMs = unique(["NGG";"NAG";"NGRRT";"NGRRN";"NNGRRT";"NGGRRT";"TTGAAT";"TTGGGT";" ↙
NNNNGATT";"NNNNRYAC";"NNAGAAW";"NGGNG";"TTTV";"TCTA";"GTTV";"GCTV";"TATA";"TTTV";" ↙
CTT";"ATG";"AWG";"TCN";"CCN";]);
PAMsWithNoN = PAMs;
for i=1:length(PAMsWithNoN)
    PAMsWithNoN(i) = removeNsFromEnds(PAMsWithNoN(i));
end
PAMsWithNoN = unique(PAMsWithNoN);

PAMsATCG = "";
for i=1:length(PAMsWithNoN)
    PAMsATCG = [PAMsATCG ChangePAMs2ATCG(PAMsWithNoN(i))];
end
PAMsATCG(1) = []; %remove the empty string at the beginning
PAMsATCG = unique(PAMsATCG);

%genes
%**************************************************
[results] = getPAMinGenes(phageSeq, geneStartEnd, PAMs, direction, genesLength, genes, ↙
essential);

%write genes into files, according to type of promoter
%**************************************************
writeGenesAccordingToPromotersToFile(genes, genesPromoters);

%promoters
%**************************************************
% promoterLengthArray = [10 20 30 40 50 60];
promoterLengthArray = [60];
```

```
% [earlyPromotersPAMs, middlePromotersPAMs, latePromotersPAMs, ~, ~, ~] = ↙
getNumberOfPromotersContainPAMs(phageSeq, PAMs, promoters, promotersLoc, ↙
promotersDirection, [10 20 30 40 50 60], 45);
[earlyPromotersPAMs, middlePromotersPAMs, latePromotersPAMs, ~, ~, ~] = ↙
getNumberOfPromotersContainPAMs(phageSeq, PAMsATCG, promoters, promotersLoc, ↙
promotersDirection, promoterLengthArray);

% Controls for promoters
%**************************************************
allDuplets = ["AA";"AT";"AC";"AG";"TA";"TT";"TC";"TG";"CA";"CT";"CC";"CG";"GA";"GT";" ↙
GC";"GG"];
allTriplets = ["AAA";"AAT";"AAC";"AAG";"ATA";"ATT";"ATC";"ATG";"ACA";"ACT";"ACC";" ↙
ACG";"AGA";"AGT";"AGC";"AGG";"TAA";"TAT";"TAC";"TAG";"TTA";"TTT";"TTC";"TTG";"TCA";" ↙
TCT";"TCC";"TCG";"TGA";"TGT";"TGC";"TGG";"CAA";"CAT";"CAC";"CAG";"CTA";"CTT";"CTC";" ↙
CTG";"CCA";"CCT";"CCC";"CCG";"CGA";"CGT";"CGC";"CGG";"GAA";"GAT";"GAC";"GAG";"GTA";" ↙
GTT";"GTC";"GTG";"GCA";"GCT";"GCC";"GCG";"GGA";"GGT";"GGC";"GGG"];
all4oligo = ["AAAA";"AAAT";"AAAC";"AAAG";"AATA";"AATT";"AATC";"AATG";"AACA";"AACT";" ↙
AACC";"AACG";"AAGA";"AAGT";"AAGC";"AAGG";"ATAA";"ATAT";"ATAC";"ATAG";"ATTA";"ATTT";" ↙
ATTC";"ATTG";"ATCA";"ATCT";"ATCC";"ATCG";"ATGA";"ATGT";"ATGC";"ATGG";"ACAA";"ACAT";" ↙
ACAC";"ACAG";"ACTA";"ACTT";"ACTC";"ACTG";"ACCA";"ACCT";"ACCC";"ACCG";"ACGA";"ACGT";" ↙
ACGC";"ACGG";"AGAA";"AGAT";"AGAC";"AGAG";"AGTA";"AGTT";"AGTC";"AGTG";"AGCA";"AGCT";" ↙
AGCC";"AGCG";"AGGA";"AGGT";"AGGC";"AGGG";"TAAA";"TAAT";"TAAC";"TAAG";"TATA";"TATT";" ↙
TATC";"TATG";"TACA";"TACT";"TACC";"TACG";"TAGA";"TAGT";"TAGC";"TAGG";"TTAA";"TTAT";" ↙
TTAC";"TTAG";"TTTA";"TTTT";"TTTC";"TTTG";"TTCA";"TTCT";"TTCC";"TTCG";"TTGA";"TTGT";" ↙
TTGC";"TTGG";"TCAA";"TCAT";"TCAC";"TCAG";"TCTA";"TCTT";"TCTC";"TCTG";"TCCA";"TCCT";" ↙
TCCC";"TCCG";"TCGA";"TCGT";"TCGC";"TCGG";"TGAA";"TGAT";"TGAC";"TGAG";"TGTA";"TGTT";" ↙
TGTC";"TGTG";"TGCA";"TGCT";"TGCC";"TGCG";"TGGA";"TGGT";"TGGC";"TGGG";"CAAA";"CAAT";" ↙
CAAC";"CAAG";"CATA";"CATT";"CATC";"CATG";"CACA";"CACT";"CACC";"CACG";"CAGA";"CAGT";" ↙
CAGC";"CAGG";"CTAA";"CTAT";"CTAC";"CTAG";"CTTA";"CTTT";"CTTC";"CTTG";"CTCA";"CTCT";" ↙
CTCC";"CTCG";"CTGA";"CTGT";"CTGC";"CTGG";"CCAA";"CCAT";"CCAC";"CCAG";"CCTA";"CCTT";" ↙
CCTC";"CCTG";"CCCA";"CCCT";"CCCC";"CCCG";"CCGA";"CCGT";"CCGC";"CCGG";"CGAA";"CGAT";" ↙
CGAC";"CGAG";"CGTA";"CGTT";"CGTC";"CGTG";"CGCA";"CGCT";"CGCC";"CGCG";"CGGA";"CGGT";" ↙
CGGC";"CGGG";"GAAA";"GAAT";"GAAC";"GAAG";"GATA";"GATT";"GATC";"GATG";"GACA";"GACT";" ↙
GACC";"GACG";"GAGA";"GAGT";"GAGC";"GAGG";"GTAA";"GTAT";"GTAC";"GTAG";"GTTA";"GTTT";" ↙
GTTC";"GTTG";"GTCA";"GTCT";"GTCC";"GTCG";"GTGA";"GTGT";"GTGC";"GTGG";"GCAA";"GCAT";" ↙
GCAC";"GCAG";"GCTA";"GCTT";"GCTC";"GCTG";"GCCA";"GCCT";"GCCC";"GCCG";"GCGA";"GCGT";" ↙
GCGC";"GCGG";"GGAA";"GGAT";"GGAC";"GGAG";"GGTA";"GGTT";"GGTC";"GGTG";"GGCA";"GGCT";" ↙
GGCC";"GGCG";"GGGA";"GGGT";"GGGC";"GGGG"];
all5oligo = ["AAAAA";"AAAAT";"AAAAC";"AAAAG";"AAATA";"AAATT";"AAATC";"AAATG";"AAACA";" ↙
AAACT";"AAACC";"AAACG";"AAAGA";"AAAGT";"AAAGC";"AAAGG";"AATAA";"AATAT";"AATAC";" ↙
AATAG";"AATTA";"AATTT";"AATTC";"AATTG";"AATCA";"AATCT";"AATCC";"AATCG";"AATGA";" ↙
AATGT";"AATGC";"AATGG";"AACAA";"AACAT";"AACAC";"AACAG";"AACTA";"AACTT";"AACTC";" ↙
AACTG";"AACCA";"AACCT";"AACCC";"AACCG";"AACGA";"AACGT";"AACGC";"AACGG";"AAGAA";" ↙
AAGAT";"AAGAC";"AAGAG";"AAGTA";"AAGTT";"AAGTC";"AAGTG";"AAGCA";"AAGCT";"AAGCC";" ↙
AAGCG";"AAGGA";"AAGGT";"AAGGC";"AAGGG";"ATAAA";"ATAAT";"ATAAC";"ATAAG";"ATATA";" ↙
ATATT";"ATATC";"ATATG";"ATACA";"ATACT";"ATACC";"ATACG";"ATAGA";"ATAGT";"ATAGC";" ↙
ATAGG";"ATTAA";"ATTAT";"ATTAC";"ATTAG";"ATTTA";"ATTTT";"ATTTC";"ATTTG";"ATTCA";" ↙
ATTCT";"ATTCC";"ATTCG";"ATTGA";"ATTGT";"ATTGC";"ATTGG";"ATCAA";"ATCAT";"ATCAC";" ↙
ATCAG";"ATCTA";"ATCTT";"ATCTC";"ATCTG";"ATCCA";"ATCCT";"ATCCC";"ATCCG";"ATCGA";" ↙
ATCGT";"ATCGC";"ATCGG";"ATGAA";"ATGAT";"ATGAC";"ATGAG";"ATGTA";"ATGTT";"ATGTC";" ↙
ATGTG";"ATGCA";"ATGCT";"ATGCC";"ATGCG";"ATGGA";"ATGGT";"ATGGC";"ATGGG";"ACAAA";" ↙
ACAAT";"ACAAC";"ACAAG";"ACATA";"ACATT";"ACATC";"ACATG";"ACACA";"ACACT";"ACACC";" ↙
ACACG";"ACAGA";"ACAGT";"ACAGC";"ACAGG";"ACTAA";"ACTAT";"ACTAC";"ACTAG";"ACTTA";" ↙
ACTTT";"ACTTC";"ACTTG";"ACTCA";"ACTCT";"ACTCC";"ACTCG";"ACTGA";"ACTGT";"ACTGC";" ↙
ACTGG";"ACCAA";"ACCAT";"ACCAC";"ACCAG";"ACCTA";"ACCTT";"ACCTC";"ACCTG";"ACCCA";" ↙
```

ACCCT";"ACCCC";"ACCCG";"ACCGA";"ACCGT";"ACCGC";"ACCGG";"ACGAA";"ACGAT";"ACGAC";" ↙
ACGAG";"ACGTA";"ACGTT";"ACGTC";"ACGTG";"ACGCA";"ACGCT";"ACGCC";"ACGCG";"ACGGA";" ↙
ACGGT";"ACGGC";"ACGGG";"AGAAA";"AGAAT";"AGAAC";"AGAAG";"AGATA";"AGATT";"AGATC";" ↙
AGATG";"AGACA";"AGACT";"AGACC";"AGACG";"AGAGA";"AGAGT";"AGAGC";"AGAGG";"AGTAA";" ↙
AGTAT";"AGTAC";"AGTAG";"AGTTA";"AGTTT";"AGTTC";"AGTTG";"AGTCA";"AGTCT";"AGTCC";" ↙
AGTCG";"AGTGA";"AGTGT";"AGTGC";"AGTGG";"AGCAA";"AGCAT";"AGCAC";"AGCAG";"AGCTA";" ↙
AGCTT";"AGCTC";"AGCTG";"AGCCA";"AGCCT";"AGCCC";"AGCCG";"AGCGA";"AGCGT";"AGCGC";" ↙
AGCGG";"AGGAA";"AGGAT";"AGGAC";"AGGAG";"AGGTA";"AGGTT";"AGGTC";"AGGTG";"AGGCA";" ↙
AGGCT";"AGGCC";"AGGCG";"AGGGA";"AGGGT";"AGGGC";"AGGGG";"TAAAA";"TAAAT";"TAAAC";" ↙
TAAAG";"TAATA";"TAATT";"TAATC";"TAATG";"TAACA";"TAACT";"TAACC";"TAACG";"TAAGA";" ↙
TAAGT";"TAAGC";"TAAGG";"TATAA";"TATAT";"TATAC";"TATAG";"TATTA";"TATTT";"TATTC";" ↙
TATTG";"TATCA";"TATCT";"TATCC";"TATCG";"TATGA";"TATGT";"TATGC";"TATGG";"TACAA";" ↙
TACAT";"TACAC";"TACAG";"TACTA";"TACTT";"TACTC";"TACTG";"TACCA";"TACCT";"TACCC";" ↙
TACCG";"TACGA";"TACGT";"TACGC";"TACGG";"TAGAA";"TAGAT";"TAGAC";"TAGAG";"TAGTA";" ↙
TAGTT";"TAGTC";"TAGTG";"TAGCA";"TAGCT";"TAGCC";"TAGCG";"TAGGA";"TAGGT";"TAGGC";" ↙
TAGGG";"TTAAA";"TTAAT";"TTAAC";"TTAAG";"TTATA";"TTATT";"TTATC";"TTATG";"TTACA";" ↙
TTACT";"TTACC";"TTACG";"TTAGA";"TTAGT";"TTAGC";"TTAGG";"TTTAA";"TTTAT";"TTTAC";" ↙
TTTAG";"TTTTA";"TTTTT";"TTTTC";"TTTTG";"TTTCA";"TTTCT";"TTTCC";"TTTCG";"TTTGA";" ↙
TTTGT";"TTTGC";"TTTGG";"TTCAA";"TTCAT";"TTCAC";"TTCAG";"TTCTA";"TTCTT";"TTCTC";" ↙
TTCTG";"TTCCA";"TTCCT";"TTCCC";"TTCCG";"TTCGA";"TTCGT";"TTCGC";"TTCGG";"TTGAA";" ↙
TTGAT";"TTGAC";"TTGAG";"TTGTA";"TTGTT";"TTGTC";"TTGTG";"TTGCA";"TTGCT";"TTGCC";" ↙
TTGCG";"TTGGA";"TTGGT";"TTGGC";"TTGGG";"TCAAA";"TCAAT";"TCAAC";"TCAAG";"TCATA";" ↙
TCATT";"TCATC";"TCATG";"TCACA";"TCACT";"TCACC";"TCACG";"TCAGA";"TCAGT";"TCAGC";" ↙
TCAGG";"TCTAA";"TCTAT";"TCTAC";"TCTAG";"TCTTA";"TCTTT";"TCTTC";"TCTTG";"TCTCA";" ↙
TCTCT";"TCTCC";"TCTCG";"TCTGA";"TCTGT";"TCTGC";"TCTGG";"TCCAA";"TCCAT";"TCCAC";" ↙
TCCAG";"TCCTA";"TCCTT";"TCCTC";"TCCTG";"TCCCA";"TCCCT";"TCCCC";"TCCCG";"TCCGA";" ↙
TCCGT";"TCCGC";"TCCGG";"TCGAA";"TCGAT";"TCGAC";"TCGAG";"TCGTA";"TCGTT";"TCGTC";" ↙
TCGTG";"TCGCA";"TCGCT";"TCGCC";"TCGCG";"TCGGA";"TCGGT";"TCGGC";"TCGGG";"TGAAA";" ↙
TGAAT";"TGAAC";"TGAAG";"TGATA";"TGATT";"TGATC";"TGATG";"TGACA";"TGACT";"TGACC";" ↙
TGACG";"TGAGA";"TGAGT";"TGAGC";"TGAGG";"TGTAA";"TGTAT";"TGTAC";"TGTAG";"TGTTA";" ↙
TGTTT";"TGTTC";"TGTTG";"TGTCA";"TGTCT";"TGTCC";"TGTCG";"TGTGA";"TGTGT";"TGTGC";" ↙
TGTGG";"TGCAA";"TGCAT";"TGCAC";"TGCAG";"TGCTA";"TGCTT";"TGCTC";"TGCTG";"TGCCA";" ↙
TGCCT";"TGCCC";"TGCCG";"TGCGA";"TGCGT";"TGCGC";"TGCGG";"TGGAA";"TGGAT";"TGGAC";" ↙
TGGAG";"TGGTA";"TGGTT";"TGGTC";"TGGTG";"TGGCA";"TGGCT";"TGGCC";"TGGCG";"TGGGA";" ↙
TGGGT";"TGGGC";"TGGGG";"CAAAA";"CAAAT";"CAAAC";"CAAAG";"CAATA";"CAATT";"CAATC";" ↙
CAATG";"CAACA";"CAACT";"CAACC";"CAACG";"CAAGA";"CAAGT";"CAAGC";"CAAGG";"CATAA";" ↙
CATAT";"CATAC";"CATAG";"CATTA";"CATTT";"CATTC";"CATTG";"CATCA";"CATCT";"CATCC";" ↙
CATCG";"CATGA";"CATGT";"CATGC";"CATGG";"CACAA";"CACAT";"CACAC";"CACAG";"CACTA";" ↙
CACTT";"CACTC";"CACTG";"CACCA";"CACCT";"CACCC";"CACCG";"CACGA";"CACGT";"CACGC";" ↙
CACGG";"CAGAA";"CAGAT";"CAGAC";"CAGAG";"CAGTA";"CAGTT";"CAGTC";"CAGTG";"CAGCA";" ↙
CAGCT";"CAGCC";"CAGCG";"CAGGA";"CAGGT";"CAGGC";"CAGGG";"CTAAA";"CTAAT";"CTAAC";" ↙
CTAAG";"CTATA";"CTATT";"CTATC";"CTATG";"CTACA";"CTACT";"CTACC";"CTACG";"CTAGA";" ↙
CTAGT";"CTAGC";"CTAGG";"CTTAA";"CTTAT";"CTTAC";"CTTAG";"CTTTA";"CTTTT";"CTTTC";" ↙
CTTTG";"CTTCA";"CTTCT";"CTTCC";"CTTCG";"CTTGA";"CTTGT";"CTTGC";"CTTGG";"CTCAA";" ↙
CTCAT";"CTCAC";"CTCAG";"CTCTA";"CTCTT";"CTCTC";"CTCTG";"CTCCA";"CTCCT";"CTCCC";" ↙
CTCCG";"CTCGA";"CTCGT";"CTCGC";"CTCGG";"CTGAA";"CTGAT";"CTGAC";"CTGAG";"CTGTA";" ↙
CTGTT";"CTGTC";"CTGTG";"CTGCA";"CTGCT";"CTGCC";"CTGCG";"CTGGA";"CTGGT";"CTGGC";" ↙
CTGGG";"CCAAA";"CCAAT";"CCAAC";"CCAAG";"CCATA";"CCATT";"CCATC";"CCATG";"CCACA";" ↙
CCACT";"CCACC";"CCACG";"CCAGA";"CCAGT";"CCAGC";"CCAGG";"CCTAA";"CCTAT";"CCTAC";" ↙
CCTAG";"CCTTA";"CCTTT";"CCTTC";"CCTTG";"CCTCA";"CCTCT";"CCTCC";"CCTCG";"CCTGA";" ↙
CCTGT";"CCTGC";"CCTGG";"CCCAA";"CCCAT";"CCCAC";"CCCAG";"CCCTA";"CCCTT";"CCCTC";" ↙
CCCTG";"CCCCA";"CCCCT";"CCCCC";"CCCCG";"CCCGA";"CCCGT";"CCCGC";"CCCGG";"CCGAA";" ↙
CCGAT";"CCGAC";"CCGAG";"CCGTA";"CCGTT";"CCGTC";"CCGTG";"CCGCA";"CCGCT";"CCGCC";" ↙
CCGCG";"CCGGA";"CCGGT";"CCGGC";"CCGGG";"CGAAA";"CGAAT";"CGAAC";"CGAAG";"CGATA";" ↙
CGATT";"CGATC";"CGATG";"CGACA";"CGACT";"CGACC";"CGACG";"CGAGA";"CGAGT";"CGAGC";" ↙
CGAGG";"CGTAA";"CGTAT";"CGTAC";"CGTAG";"CGTTA";"CGTTT";"CGTTC";"CGTTG";"CGTCA";" ↙

```
CGTCT";"CGTCC";"CGTCG";"CGTGA";"CGTGT";"CGTGC";"CGTGG";"CGCAA";"CGCAT";"CGCAC";" ↙
CGCAG";"CGCTA";"CGCTT";"CGCTC";"CGCTG";"CGCCA";"CGCCT";"CGCCC";"CGCCG";"CGCGA";" ↙
CGCGT";"CGCGC";"CGCGG";"CGGAA";"CGGAT";"CGGAC";"CGGAG";"CGGTA";"CGGTT";"CGGTC";" ↙
CGGTG";"CGGCA";"CGGCT";"CGGCC";"CGGCG";"CGGGA";"CGGGT";"CGGGC";"CGGGG";"GAAAA";" ↙
GAAAT";"GAAAC";"GAAAG";"GAATA";"GAATT";"GAATC";"GAATG";"GAACA";"GAACT";"GAACC";" ↙
GAACG";"GAAGA";"GAAGT";"GAAGC";"GAAGG";"GATAA";"GATAT";"GATAC";"GATAG";"GATTA";" ↙
GATTT";"GATTC";"GATTG";"GATCA";"GATCT";"GATCC";"GATCG";"GATGA";"GATGT";"GATGC";" ↙
GATGG";"GACAA";"GACAT";"GACAC";"GACAG";"GACTA";"GACTT";"GACTC";"GACTG";"GACCA";" ↙
GACCT";"GACCC";"GACCG";"GACGA";"GACGT";"GACGC";"GACGG";"GAGAA";"GAGAT";"GAGAC";" ↙
GAGAG";"GAGTA";"GAGTT";"GAGTC";"GAGTG";"GAGCA";"GAGCT";"GAGCC";"GAGCG";"GAGGA";" ↙
GAGGT";"GAGGC";"GAGGG";"GTAAA";"GTAAT";"GTAAC";"GTAAG";"GTATA";"GTATT";"GTATC";" ↙
GTATG";"GTACA";"GTACT";"GTACC";"GTACG";"GTAGA";"GTAGT";"GTAGC";"GTAGG";"GTTAA";" ↙
GTTAT";"GTTAC";"GTTAG";"GTTTA";"GTTTT";"GTTTC";"GTTTG";"GTTCA";"GTTCT";"GTTCC";" ↙
GTTCG";"GTTGA";"GTTGT";"GTTGC";"GTTGG";"GTCAA";"GTCAT";"GTCAC";"GTCAG";"GTCTA";" ↙
GTCTT";"GTCTC";"GTCTG";"GTCCA";"GTCCT";"GTCCC";"GTCCG";"GTCGA";"GTCGT";"GTCGC";" ↙
GTCGG";"GTGAA";"GTGAT";"GTGAC";"GTGAG";"GTGTA";"GTGTT";"GTGTC";"GTGTG";"GTGCA";" ↙
GTGCT";"GTGCC";"GTGCG";"GTGGA";"GTGGT";"GTGGC";"GTGGG";"GCAAA";"GCAAT";"GCAAC";" ↙
GCAAG";"GCATA";"GCATT";"GCATC";"GCATG";"GCACA";"GCACT";"GCACC";"GCACG";"GCAGA";" ↙
GCAGT";"GCAGC";"GCAGG";"GCTAA";"GCTAT";"GCTAC";"GCTAG";"GCTTA";"GCTTT";"GCTTC";" ↙
GCTTG";"GCTCA";"GCTCT";"GCTCC";"GCTCG";"GCTGA";"GCTGT";"GCTGC";"GCTGG";"GCCAA";" ↙
GCCAT";"GCCAC";"GCCAG";"GCCTA";"GCCTT";"GCCTC";"GCCTG";"GCCCA";"GCCCT";"GCCCC";" ↙
GCCCG";"GCCGA";"GCCGT";"GCCGC";"GCCGG";"GCGAA";"GCGAT";"GCGAC";"GCGAG";"GCGTA";" ↙
GCGTT";"GCGTC";"GCGTG";"GCGCA";"GCGCT";"GCGCC";"GCGCG";"GCGGA";"GCGGT";"GCGGC";" ↙
GCGGG";"GGAAA";"GGAAT";"GGAAC";"GGAAG";"GGATA";"GGATT";"GGATC";"GGATG";"GGACA";" ↙
GGACT";"GGACC";"GGACG";"GGAGA";"GGAGT";"GGAGC";"GGAGG";"GGTAA";"GGTAT";"GGTAC";" ↙
GGTAG";"GGTTA";"GGTTT";"GGTTC";"GGTTG";"GGTCA";"GGTCT";"GGTCC";"GGTCG";"GGTGA";" ↙
GGTGT";"GGTGC";"GGTGG";"GGCAA";"GGCAT";"GGCAC";"GGCAG";"GGCTA";"GGCTT";"GGCTC";" ↙
GGCTG";"GGCCA";"GGCCT";"GGCCC";"GGCCG";"GGCGA";"GGCGT";"GGCGC";"GGCGG";"GGGAA";" ↙
GGGAT";"GGGAC";"GGGAG";"GGGTA";"GGGTT";"GGGTC";"GGGTG";"GGGCA";"GGGCT";"GGGCC";" ↙
GGGCG";"GGGGA";"GGGGT";"GGGGC";"GGGGG"];
all6oligo = ["AAAAAA";"AAAAAT";"AAAAAC";"AAAAAG";"AAAATA";"AAAATT";"AAAATC";"AAAATG";" ↙
AAAACA";"AAAACT";"AAAACC";"AAAACG";"AAAAGA";"AAAAGT";"AAAAGC";"AAAAGG";"AAATAA";" ↙
AAATAT";"AAATAC";"AAATAG";"AAATTA";"AAATTT";"AAATTC";"AAATTG";"AAATCA";"AAATCT";" ↙
AAATCC";"AAATCG";"AAATGA";"AAATGT";"AAATGC";"AAATGG";"AAACAA";"AAACAT";"AAACAC";" ↙
AAACAG";"AAACTA";"AAACTT";"AAACTC";"AAACTG";"AAACCA";"AAACCT";"AAACCC";"AAACCG";" ↙
AAACGA";"AAACGT";"AAACGC";"AAACGG";"AAAGAA";"AAAGAT";"AAAGAC";"AAAGAG";"AAAGTA";" ↙
AAAGTT";"AAAGTC";"AAAGTG";"AAAGCA";"AAAGCT";"AAAGCC";"AAAGCG";"AAAGGA";"AAAGGT";" ↙
AAAGGC";"AAAGGG";"AATAAA";"AATAAT";"AATAAC";"AATAAG";"AATATA";"AATATT";"AATATC";" ↙
AATATG";"AATACA";"AATACT";"AATACC";"AATACG";"AATAGA";"AATAGT";"AATAGC";"AATAGG";" ↙
AATTAA";"AATTAT";"AATTAC";"AATTAG";"AATTTA";"AATTTT";"AATTTC";"AATTTG";"AATTCA";" ↙
AATTCT";"AATTCC";"AATTCG";"AATTGA";"AATTGT";"AATTGC";"AATTGG";"AATCAA";"AATCAT";" ↙
AATCAC";"AATCAG";"AATCTA";"AATCTT";"AATCTC";"AATCTG";"AATCCA";"AATCCT";"AATCCC";" ↙
AATCCG";"AATCGA";"AATCGT";"AATCGC";"AATCGG";"AATGAA";"AATGAT";"AATGAC";"AATGAG";" ↙
AATGTA";"AATGTT";"AATGTC";"AATGTG";"AATGCA";"AATGCT";"AATGCC";"AATGCG";"AATGGA";" ↙
AATGGT";"AATGGC";"AATGGG";"AACAAA";"AACAAT";"AACAAC";"AACAAG";"AACATA";"AACATT";" ↙
AACATC";"AACATG";"AACACA";"AACACT";"AACACC";"AACACG";"AACAGA";"AACAGT";"AACAGC";" ↙
AACAGG";"AACTAA";"AACTAT";"AACTAC";"AACTAG";"AACTTA";"AACTTT";"AACTTC";"AACTTG";" ↙
AACTCA";"AACTCT";"AACTCC";"AACTCG";"AACTGA";"AACTGT";"AACTGC";"AACTGG";"AACCAA";" ↙
AACCAT";"AACCAC";"AACCAG";"AACCTA";"AACCTT";"AACCTC";"AACCTG";"AACCCA";"AACCCT";" ↙
AACCCC";"AACCCG";"AACCGA";"AACCGT";"AACCGC";"AACCGG";"AACGAA";"AACGAT";"AACGAC";" ↙
AACGAG";"AACGTA";"AACGTT";"AACGTC";"AACGTG";"AACGCA";"AACGCT";"AACGCC";"AACGCG";" ↙
AACGGA";"AACGGT";"AACGGC";"AACGGG";"AAGAAA";"AAGAAT";"AAGAAC";"AAGAAG";"AAGATA";" ↙
AAGATT";"AAGATC";"AAGATG";"AAGACA";"AAGACT";"AAGACC";"AAGACG";"AAGAGA";"AAGAGT";" ↙
AAGAGC";"AAGAGG";"AAGTAA";"AAGTAT";"AAGTAC";"AAGTAG";"AAGTTA";"AAGTTT";"AAGTTC";" ↙
AAGTTG";"AAGTCA";"AAGTCT";"AAGTCC";"AAGTCG";"AAGTGA";"AAGTGT";"AAGTGC";"AAGTGG";" ↙
AAGCAA";"AAGCAT";"AAGCAC";"AAGCAG";"AAGCTA";"AAGCTT";"AAGCTC";"AAGCTG";"AAGCCA";" ↙
```

AAGCCT";"AAGCCC";"AAGCCG";"AAGCGA";"AAGCGT";"AAGCGC";"AAGCGG";"AAGGAA";"AAGGAT";" ↙
AAGGAC";"AAGGAG";"AAGGTA";"AAGGTT";"AAGGTC";"AAGGTG";"AAGGCA";"AAGGCT";"AAGGCC";" ↙
AAGGCG";"AAGGGA";"AAGGGT";"AAGGGC";"AAGGGG";"ATAAAA";"ATAAAT";"ATAAAC";"ATAAAG";" ↙
ATAATA";"ATAATT";"ATAATC";"ATAATG";"ATAACA";"ATAACT";"ATAACC";"ATAACG";"ATAAGA";" ↙
ATAAGT";"ATAAGC";"ATAAGG";"ATATAA";"ATATAT";"ATATAC";"ATATAG";"ATATTA";"ATATTT";" ↙
ATATTC";"ATATTG";"ATATCA";"ATATCT";"ATATCC";"ATATCG";"ATATGA";"ATATGT";"ATATGC";" ↙
ATATGG";"ATACAA";"ATACAT";"ATACAC";"ATACAG";"ATACTA";"ATACTT";"ATACTC";"ATACTG";" ↙
ATACCA";"ATACCT";"ATACCC";"ATACCG";"ATACGA";"ATACGT";"ATACGC";"ATACGG";"ATAGAA";" ↙
ATAGAT";"ATAGAC";"ATAGAG";"ATAGTA";"ATAGTT";"ATAGTC";"ATAGTG";"ATAGCA";"ATAGCT";" ↙
ATAGCC";"ATAGCG";"ATAGGA";"ATAGGT";"ATAGGC";"ATAGGG";"ATTAAA";"ATTAAT";"ATTAAC";" ↙
ATTAAG";"ATTATA";"ATTATT";"ATTATC";"ATTATG";"ATTACA";"ATTACT";"ATTACC";"ATTACG";" ↙
ATTAGA";"ATTAGT";"ATTAGC";"ATTAGG";"ATTTAA";"ATTTAT";"ATTTAC";"ATTTAG";"ATTTTA";" ↙
ATTTTT";"ATTTTC";"ATTTTG";"ATTTCA";"ATTTCT";"ATTTCC";"ATTTCG";"ATTTGA";"ATTTGT";" ↙
ATTTGC";"ATTTGG";"ATTCAA";"ATTCAT";"ATTCAC";"ATTCAG";"ATTCTA";"ATTCTT";"ATTCTC";" ↙
ATTCTG";"ATTCCA";"ATTCCT";"ATTCCC";"ATTCCG";"ATTCGA";"ATTCGT";"ATTCGC";"ATTCGG";" ↙
ATTGAA";"ATTGAT";"ATTGAC";"ATTGAG";"ATTGTA";"ATTGTT";"ATTGTC";"ATTGTG";"ATTGCA";" ↙
ATTGCT";"ATTGCC";"ATTGCG";"ATTGGA";"ATTGGT";"ATTGGC";"ATTGGG";"ATCAAA";"ATCAAT";" ↙
ATCAAC";"ATCAAG";"ATCATA";"ATCATT";"ATCATC";"ATCATG";"ATCACA";"ATCACT";"ATCACC";" ↙
ATCACG";"ATCAGA";"ATCAGT";"ATCAGC";"ATCAGG";"ATCTAA";"ATCTAT";"ATCTAC";"ATCTAG";" ↙
ATCTTA";"ATCTTT";"ATCTTC";"ATCTTG";"ATCTCA";"ATCTCT";"ATCTCC";"ATCTCG";"ATCTGA";" ↙
ATCTGT";"ATCTGC";"ATCTGG";"ATCCAA";"ATCCAT";"ATCCAC";"ATCCAG";"ATCCTA";"ATCCTT";" ↙
ATCCTC";"ATCCTG";"ATCCCA";"ATCCCT";"ATCCCC";"ATCCCG";"ATCCGA";"ATCCGT";"ATCCGC";" ↙
ATCCGG";"ATCGAA";"ATCGAT";"ATCGAC";"ATCGAG";"ATCGTA";"ATCGTT";"ATCGTC";"ATCGTG";" ↙
ATCGCA";"ATCGCT";"ATCGCC";"ATCGCG";"ATCGGA";"ATCGGT";"ATCGGC";"ATCGGG";"ATGAAA";" ↙
ATGAAT";"ATGAAC";"ATGAAG";"ATGATA";"ATGATT";"ATGATC";"ATGATG";"ATGACA";"ATGACT";" ↙
ATGACC";"ATGACG";"ATGAGA";"ATGAGT";"ATGAGC";"ATGAGG";"ATGTAA";"ATGTAT";"ATGTAC";" ↙
ATGTAG";"ATGTTA";"ATGTTT";"ATGTTC";"ATGTTG";"ATGTCA";"ATGTCT";"ATGTCC";"ATGTCG";" ↙
ATGTGA";"ATGTGT";"ATGTGC";"ATGTGG";"ATGCAA";"ATGCAT";"ATGCAC";"ATGCAG";"ATGCTA";" ↙
ATGCTT";"ATGCTC";"ATGCTG";"ATGCCA";"ATGCCT";"ATGCCC";"ATGCCG";"ATGCGA";"ATGCGT";" ↙
ATGCGC";"ATGCGG";"ATGGAA";"ATGGAT";"ATGGAC";"ATGGAG";"ATGGTA";"ATGGTT";"ATGGTC";" ↙
ATGGTG";"ATGGCA";"ATGGCT";"ATGGCC";"ATGGCG";"ATGGGA";"ATGGGT";"ATGGGC";"ATGGGG";" ↙
ACAAAA";"ACAAAT";"ACAAAC";"ACAAAG";"ACAATA";"ACAATT";"ACAATC";"ACAATG";"ACAACA";" ↙
ACAACT";"ACAACC";"ACAACG";"ACAAGA";"ACAAGT";"ACAAGC";"ACAAGG";"ACATAA";"ACATAT";" ↙
ACATAC";"ACATAG";"ACATTA";"ACATTT";"ACATTC";"ACATTG";"ACATCA";"ACATCT";"ACATCC";" ↙
ACATCG";"ACATGA";"ACATGT";"ACATGC";"ACATGG";"ACACAA";"ACACAT";"ACACAC";"ACACAG";" ↙
ACACTA";"ACACTT";"ACACTC";"ACACTG";"ACACCA";"ACACCT";"ACACCC";"ACACCG";"ACACGA";" ↙
ACACGT";"ACACGC";"ACACGG";"ACAGAA";"ACAGAT";"ACAGAC";"ACAGAG";"ACAGTA";"ACAGTT";" ↙
ACAGTC";"ACAGTG";"ACAGCA";"ACAGCT";"ACAGCC";"ACAGCG";"ACAGGA";"ACAGGT";"ACAGGC";" ↙
ACAGGG";"ACTAAA";"ACTAAT";"ACTAAC";"ACTAAG";"ACTATA";"ACTATT";"ACTATC";"ACTATG";" ↙
ACTACA";"ACTACT";"ACTACC";"ACTACG";"ACTAGA";"ACTAGT";"ACTAGC";"ACTAGG";"ACTTAA";" ↙
ACTTAT";"ACTTAC";"ACTTAG";"ACTTTA";"ACTTTT";"ACTTTC";"ACTTTG";"ACTTCA";"ACTTCT";" ↙
ACTTCC";"ACTTCG";"ACTTGA";"ACTTGT";"ACTTGC";"ACTTGG";"ACTCAA";"ACTCAT";"ACTCAC";" ↙
ACTCAG";"ACTCTA";"ACTCTT";"ACTCTC";"ACTCTG";"ACTCCA";"ACTCCT";"ACTCCC";"ACTCCG";" ↙
ACTCGA";"ACTCGT";"ACTCGC";"ACTCGG";"ACTGAA";"ACTGAT";"ACTGAC";"ACTGAG";"ACTGTA";" ↙
ACTGTT";"ACTGTC";"ACTGTG";"ACTGCA";"ACTGCT";"ACTGCC";"ACTGCG";"ACTGGA";"ACTGGT";" ↙
ACTGGC";"ACTGGG";"ACCAAA";"ACCAAT";"ACCAAC";"ACCAAG";"ACCATA";"ACCATT";"ACCATC";" ↙
ACCATG";"ACCACA";"ACCACT";"ACCACC";"ACCACG";"ACCAGA";"ACCAGT";"ACCAGC";"ACCAGG";" ↙
ACCTAA";"ACCTAT";"ACCTAC";"ACCTAG";"ACCTTA";"ACCTTT";"ACCTTC";"ACCTTG";"ACCTCA";" ↙
ACCTCT";"ACCTCC";"ACCTCG";"ACCTGA";"ACCTGT";"ACCTGC";"ACCTGG";"ACCCAA";"ACCCAT";" ↙
ACCCAC";"ACCCAG";"ACCCTA";"ACCCTT";"ACCCTC";"ACCCTG";"ACCCCA";"ACCCCT";"ACCCCC";" ↙
ACCCCG";"ACCCGA";"ACCCGT";"ACCCGC";"ACCCGG";"ACCGAA";"ACCGAT";"ACCGAC";"ACCGAG";" ↙
ACCGTA";"ACCGTT";"ACCGTC";"ACCGTG";"ACCGCA";"ACCGCT";"ACCGCC";"ACCGCG";"ACCGGA";" ↙
ACCGGT";"ACCGGC";"ACCGGG";"ACGAAA";"ACGAAT";"ACGAAC";"ACGAAG";"ACGATA";"ACGATT";" ↙
ACGATC";"ACGATG";"ACGACA";"ACGACT";"ACGACC";"ACGACG";"ACGAGA";"ACGAGT";"ACGAGC";" ↙
ACGAGG";"ACGTAA";"ACGTAT";"ACGTAC";"ACGTAG";"ACGTTA";"ACGTTT";"ACGTTC";"ACGTTG";" ↙
ACGTCA";"ACGTCT";"ACGTCC";"ACGTCG";"ACGTGA";"ACGTGT";"ACGTGC";"ACGTGG";"ACGCAA";" ↙

```
ACGCAT";"ACGCAC";"ACGCAG";"ACGCTA";"ACGCTT";"ACGCTC";"ACGCTG";"ACGCCA";"ACGCCT";" ↙
ACGCCC";"ACGCCG";"ACGCGA";"ACGCGT";"ACGCGC";"ACGCGG";"ACGGAA";"ACGGAT";"ACGGAC";" ↙
ACGGAG";"ACGGTA";"ACGGTT";"ACGGTC";"ACGGTG";"ACGGCA";"ACGGCT";"ACGGCC";"ACGGCG";" ↙
ACGGGA";"ACGGGT";"ACGGGC";"ACGGGG";"AGAAAA";"AGAAAT";"AGAAAC";"AGAAAG";"AGAATA";" ↙
AGAATT";"AGAATC";"AGAATG";"AGAACA";"AGAACT";"AGAACC";"AGAACG";"AGAAGA";"AGAAGT";" ↙
AGAAGC";"AGAAGG";"AGATAA";"AGATAT";"AGATAC";"AGATAG";"AGATTA";"AGATTT";"AGATTC";" ↙
AGATTG";"AGATCA";"AGATCT";"AGATCC";"AGATCG";"AGATGA";"AGATGT";"AGATGC";"AGATGG";" ↙
AGACAA";"AGACAT";"AGACAC";"AGACAG";"AGACTA";"AGACTT";"AGACTC";"AGACTG";"AGACCA";" ↙
AGACCT";"AGACCC";"AGACCG";"AGACGA";"AGACGT";"AGACGC";"AGACGG";"AGAGAA";"AGAGAT";" ↙
AGAGAC";"AGAGAG";"AGAGTA";"AGAGTT";"AGAGTC";"AGAGTG";"AGAGCA";"AGAGCT";"AGAGCC";" ↙
AGAGCG";"AGAGGA";"AGAGGT";"AGAGGC";"AGAGGG";"AGTAAA";"AGTAAT";"AGTAAC";"AGTAAG";" ↙
AGTATA";"AGTATT";"AGTATC";"AGTATG";"AGTACA";"AGTACT";"AGTACC";"AGTACG";"AGTAGA";" ↙
AGTAGT";"AGTAGC";"AGTAGG";"AGTTAA";"AGTTAT";"AGTTAC";"AGTTAG";"AGTTTA";"AGTTTT";" ↙
AGTTTC";"AGTTTG";"AGTTCA";"AGTTCT";"AGTTCC";"AGTTCG";"AGTTGA";"AGTTGT";"AGTTGC";" ↙
AGTTGG";"AGTCAA";"AGTCAT";"AGTCAC";"AGTCAG";"AGTCTA";"AGTCTT";"AGTCTC";"AGTCTG";" ↙
AGTCCA";"AGTCCT";"AGTCCC";"AGTCCG";"AGTCGA";"AGTCGT";"AGTCGC";"AGTCGG";"AGTGAA";" ↙
AGTGAT";"AGTGAC";"AGTGAG";"AGTGTA";"AGTGTT";"AGTGTC";"AGTGTG";"AGTGCA";"AGTGCT";" ↙
AGTGCC";"AGTGCG";"AGTGGA";"AGTGGT";"AGTGGC";"AGTGGG";"AGCAAA";"AGCAAT";"AGCAAC";" ↙
AGCAAG";"AGCATA";"AGCATT";"AGCATC";"AGCATG";"AGCACA";"AGCACT";"AGCACC";"AGCACG";" ↙
AGCAGA";"AGCAGT";"AGCAGC";"AGCAGG";"AGCTAA";"AGCTAT";"AGCTAC";"AGCTAG";"AGCTTA";" ↙
AGCTTT";"AGCTTC";"AGCTTG";"AGCTCA";"AGCTCT";"AGCTCC";"AGCTCG";"AGCTGA";"AGCTGT";" ↙
AGCTGC";"AGCTGG";"AGCCAA";"AGCCAT";"AGCCAC";"AGCCAG";"AGCCTA";"AGCCTT";"AGCCTC";" ↙
AGCCTG";"AGCCCA";"AGCCCT";"AGCCCC";"AGCCCG";"AGCCGA";"AGCCGT";"AGCCGC";"AGCCGG";" ↙
AGCGAA";"AGCGAT";"AGCGAC";"AGCGAG";"AGCGTA";"AGCGTT";"AGCGTC";"AGCGTG";"AGCGCA";" ↙
AGCGCT";"AGCGCC";"AGCGCG";"AGCGGA";"AGCGGT";"AGCGGC";"AGCGGG";"AGGAAA";"AGGAAT";" ↙
AGGAAC";"AGGAAG";"AGGATA";"AGGATT";"AGGATC";"AGGATG";"AGGACA";"AGGACT";"AGGACC";" ↙
AGGACG";"AGGAGA";"AGGAGT";"AGGAGC";"AGGAGG";"AGGTAA";"AGGTAT";"AGGTAC";"AGGTAG";" ↙
AGGTTA";"AGGTTT";"AGGTTC";"AGGTTG";"AGGTCA";"AGGTCT";"AGGTCC";"AGGTCG";"AGGTGA";" ↙
AGGTGT";"AGGTGC";"AGGTGG";"AGGCAA";"AGGCAT";"AGGCAC";"AGGCAG";"AGGCTA";"AGGCTT";" ↙
AGGCTC";"AGGCTG";"AGGCCA";"AGGCCT";"AGGCCC";"AGGCCG";"AGGCGA";"AGGCGT";"AGGCGC";" ↙
AGGCGG";"AGGGAA";"AGGGAT";"AGGGAC";"AGGGAG";"AGGGTA";"AGGGTT";"AGGGTC";"AGGGTG";" ↙
AGGGCA";"AGGGCT";"AGGGCC";"AGGGCG";"AGGGGA";"AGGGGT";"AGGGGC";"AGGGGG";"TAAAAA";" ↙
TAAAAT";"TAAAAC";"TAAAAG";"TAAATA";"TAAATT";"TAAATC";"TAAATG";"TAAACA";"TAAACT";" ↙
TAAACC";"TAAACG";"TAAAGA";"TAAAGT";"TAAAGC";"TAAAGG";"TAATAA";"TAATAT";"TAATAC";" ↙
TAATAG";"TAATTA";"TAATTT";"TAATTC";"TAATTG";"TAATCA";"TAATCT";"TAATCC";"TAATCG";" ↙
TAATGA";"TAATGT";"TAATGC";"TAATGG";"TAACAA";"TAACAT";"TAACAC";"TAACAG";"TAACTA";" ↙
TAACTT";"TAACTC";"TAACTG";"TAACCA";"TAACCT";"TAACCC";"TAACCG";"TAACGA";"TAACGT";" ↙
TAACGC";"TAACGG";"TAAGAA";"TAAGAT";"TAAGAC";"TAAGAG";"TAAGTA";"TAAGTT";"TAAGTC";" ↙
TAAGTG";"TAAGCA";"TAAGCT";"TAAGCC";"TAAGCG";"TAAGGA";"TAAGGT";"TAAGGC";"TAAGGG";" ↙
TATAAA";"TATAAT";"TATAAC";"TATAAG";"TATATA";"TATATT";"TATATC";"TATATG";"TATACA";" ↙
TATACT";"TATACC";"TATACG";"TATAGA";"TATAGT";"TATAGC";"TATAGG";"TATTAA";"TATTAT";" ↙
TATTAC";"TATTAG";"TATTTA";"TATTTT";"TATTTC";"TATTTG";"TATTCA";"TATTCT";"TATTCC";" ↙
TATTCG";"TATTGA";"TATTGT";"TATTGC";"TATTGG";"TATCAA";"TATCAT";"TATCAC";"TATCAG";" ↙
TATCTA";"TATCTT";"TATCTC";"TATCTG";"TATCCA";"TATCCT";"TATCCC";"TATCCG";"TATCGA";" ↙
TATCGT";"TATCGC";"TATCGG";"TATGAA";"TATGAT";"TATGAC";"TATGAG";"TATGTA";"TATGTT";" ↙
TATGTC";"TATGTG";"TATGCA";"TATGCT";"TATGCC";"TATGCG";"TATGGA";"TATGGT";"TATGGC";" ↙
TATGGG";"TACAAA";"TACAAT";"TACAAC";"TACAAG";"TACATA";"TACATT";"TACATC";"TACATG";" ↙
TACACA";"TACACT";"TACACC";"TACACG";"TACAGA";"TACAGT";"TACAGC";"TACAGG";"TACTAA";" ↙
TACTAT";"TACTAC";"TACTAG";"TACTTA";"TACTTT";"TACTTC";"TACTTG";"TACTCA";"TACTCT";" ↙
TACTCC";"TACTCG";"TACTGA";"TACTGT";"TACTGC";"TACTGG";"TACCAA";"TACCAT";"TACCAC";" ↙
TACCAG";"TACCTA";"TACCTT";"TACCTC";"TACCTG";"TACCCA";"TACCCT";"TACCCC";"TACCCG";" ↙
TACCGA";"TACCGT";"TACCGC";"TACCGG";"TACGAA";"TACGAT";"TACGAC";"TACGAG";"TACGTA";" ↙
TACGTT";"TACGTC";"TACGTG";"TACGCA";"TACGCT";"TACGCC";"TACGCG";"TACGGA";"TACGGT";" ↙
TACGGC";"TACGGG";"TAGAAA";"TAGAAT";"TAGAAC";"TAGAAG";"TAGATA";"TAGATT";"TAGATC";" ↙
TAGATG";"TAGACA";"TAGACT";"TAGACC";"TAGACG";"TAGAGA";"TAGAGT";"TAGAGC";"TAGAGG";" ↙
TAGTAA";"TAGTAT";"TAGTAC";"TAGTAG";"TAGTTA";"TAGTTT";"TAGTTC";"TAGTTG";"TAGTCA";" ↙
```

TAGTCT";"TAGTCC";"TAGTCG";"TAGTGA";"TAGTGT";"TAGTGC";"TAGTGG";"TAGCAA";"TAGCAT";" ↙
TAGCAC";"TAGCAG";"TAGCTA";"TAGCTT";"TAGCTC";"TAGCTG";"TAGCCA";"TAGCCT";"TAGCCC";" ↙
TAGCCG";"TAGCGA";"TAGCGT";"TAGCGC";"TAGCGG";"TAGGAA";"TAGGAT";"TAGGAC";"TAGGAG";" ↙
TAGGTA";"TAGGTT";"TAGGTC";"TAGGTG";"TAGGCA";"TAGGCT";"TAGGCC";"TAGGCG";"TAGGGA";" ↙
TAGGGT";"TAGGGC";"TAGGGG";"TTAAAA";"TTAAAT";"TTAAAC";"TTAAAG";"TTAATA";"TTAATT";" ↙
TTAATC";"TTAATG";"TTAACA";"TTAACT";"TTAACC";"TTAACG";"TTAAGA";"TTAAGT";"TTAAGC";" ↙
TTAAGG";"TTATAA";"TTATAT";"TTATAC";"TTATAG";"TTATTA";"TTATTT";"TTATTC";"TTATTG";" ↙
TTATCA";"TTATCT";"TTATCC";"TTATCG";"TTATGA";"TTATGT";"TTATGC";"TTATGG";"TTACAA";" ↙
TTACAT";"TTACAC";"TTACAG";"TTACTA";"TTACTT";"TTACTC";"TTACTG";"TTACCA";"TTACCT";" ↙
TTACCC";"TTACCG";"TTACGA";"TTACGT";"TTACGC";"TTACGG";"TTAGAA";"TTAGAT";"TTAGAC";" ↙
TTAGAG";"TTAGTA";"TTAGTT";"TTAGTC";"TTAGTG";"TTAGCA";"TTAGCT";"TTAGCC";"TTAGCG";" ↙
TTAGGA";"TTAGGT";"TTAGGC";"TTAGGG";"TTTAAA";"TTTAAT";"TTTAAC";"TTTAAG";"TTTATA";" ↙
TTTATT";"TTTATC";"TTTATG";"TTTACA";"TTTACT";"TTTACC";"TTTACG";"TTTAGA";"TTTAGT";" ↙
TTTAGC";"TTTAGG";"TTTTAA";"TTTTAT";"TTTTAC";"TTTTAG";"TTTTTA";"TTTTTT";"TTTTTC";" ↙
TTTTTG";"TTTTCA";"TTTTCT";"TTTTCC";"TTTTCG";"TTTTGA";"TTTTGT";"TTTTGC";"TTTTGG";" ↙
TTTCAA";"TTTCAT";"TTTCAC";"TTTCAG";"TTTCTA";"TTTCTT";"TTTCTC";"TTTCTG";"TTTCCA";" ↙
TTTCCT";"TTTCCC";"TTTCCG";"TTTCGA";"TTTCGT";"TTTCGC";"TTTCGG";"TTTGAA";"TTTGAT";" ↙
TTTGAC";"TTTGAG";"TTTGTA";"TTTGTT";"TTTGTC";"TTTGTG";"TTTGCA";"TTTGCT";"TTTGCC";" ↙
TTTGCG";"TTTGGA";"TTTGGT";"TTTGGC";"TTTGGG";"TTCAAA";"TTCAAT";"TTCAAC";"TTCAAG";" ↙
TTCATA";"TTCATT";"TTCATC";"TTCATG";"TTCACA";"TTCACT";"TTCACC";"TTCACG";"TTCAGA";" ↙
TTCAGT";"TTCAGC";"TTCAGG";"TTCTAA";"TTCTAT";"TTCTAC";"TTCTAG";"TTCTTA";"TTCTTT";" ↙
TTCTTC";"TTCTTG";"TTCTCA";"TTCTCT";"TTCTCC";"TTCTCG";"TTCTGA";"TTCTGT";"TTCTGC";" ↙
TTCTGG";"TTCCAA";"TTCCAT";"TTCCAC";"TTCCAG";"TTCCTA";"TTCCTT";"TTCCTC";"TTCCTG";" ↙
TTCCCA";"TTCCCT";"TTCCCC";"TTCCCG";"TTCCGA";"TTCCGT";"TTCCGC";"TTCCGG";"TTCGAA";" ↙
TTCGAT";"TTCGAC";"TTCGAG";"TTCGTA";"TTCGTT";"TTCGTC";"TTCGTG";"TTCGCA";"TTCGCT";" ↙
TTCGCC";"TTCGCG";"TTCGGA";"TTCGGT";"TTCGGC";"TTCGGG";"TTGAAA";"TTGAAT";"TTGAAC";" ↙
TTGAAG";"TTGATA";"TTGATT";"TTGATC";"TTGATG";"TTGACA";"TTGACT";"TTGACC";"TTGACG";" ↙
TTGAGA";"TTGAGT";"TTGAGC";"TTGAGG";"TTGTAA";"TTGTAT";"TTGTAC";"TTGTAG";"TTGTTA";" ↙
TTGTTT";"TTGTTC";"TTGTTG";"TTGTCA";"TTGTCT";"TTGTCC";"TTGTCG";"TTGTGA";"TTGTGT";" ↙
TTGTGC";"TTGTGG";"TTGCAA";"TTGCAT";"TTGCAC";"TTGCAG";"TTGCTA";"TTGCTT";"TTGCTC";" ↙
TTGCTG";"TTGCCA";"TTGCCT";"TTGCCC";"TTGCCG";"TTGCGA";"TTGCGT";"TTGCGC";"TTGCGG";" ↙
TTGGAA";"TTGGAT";"TTGGAC";"TTGGAG";"TTGGTA";"TTGGTT";"TTGGTC";"TTGGTG";"TTGGCA";" ↙
TTGGCT";"TTGGCC";"TTGGCG";"TTGGGA";"TTGGGT";"TTGGGC";"TTGGGG";"TCAAAA";"TCAAAT";" ↙
TCAAAC";"TCAAAG";"TCAATA";"TCAATT";"TCAATC";"TCAATG";"TCAACA";"TCAACT";"TCAACC";" ↙
TCAACG";"TCAAGA";"TCAAGT";"TCAAGC";"TCAAGG";"TCATAA";"TCATAT";"TCATAC";"TCATAG";" ↙
TCATTA";"TCATTT";"TCATTC";"TCATTG";"TCATCA";"TCATCT";"TCATCC";"TCATCG";"TCATGA";" ↙
TCATGT";"TCATGC";"TCATGG";"TCACAA";"TCACAT";"TCACAC";"TCACAG";"TCACTA";"TCACTT";" ↙
TCACTC";"TCACTG";"TCACCA";"TCACCT";"TCACCC";"TCACCG";"TCACGA";"TCACGT";"TCACGC";" ↙
TCACGG";"TCAGAA";"TCAGAT";"TCAGAC";"TCAGAG";"TCAGTA";"TCAGTT";"TCAGTC";"TCAGTG";" ↙
TCAGCA";"TCAGCT";"TCAGCC";"TCAGCG";"TCAGGA";"TCAGGT";"TCAGGC";"TCAGGG";"TCTAAA";" ↙
TCTAAT";"TCTAAC";"TCTAAG";"TCTATA";"TCTATT";"TCTATC";"TCTATG";"TCTACA";"TCTACT";" ↙
TCTACC";"TCTACG";"TCTAGA";"TCTAGT";"TCTAGC";"TCTAGG";"TCTTAA";"TCTTAT";"TCTTAC";" ↙
TCTTAG";"TCTTTA";"TCTTTT";"TCTTTC";"TCTTTG";"TCTTCA";"TCTTCT";"TCTTCC";"TCTTCG";" ↙
TCTTGA";"TCTTGT";"TCTTGC";"TCTTGG";"TCTCAA";"TCTCAT";"TCTCAC";"TCTCAG";"TCTCTA";" ↙
TCTCTT";"TCTCTC";"TCTCTG";"TCTCCA";"TCTCCT";"TCTCCC";"TCTCCG";"TCTCGA";"TCTCGT";" ↙
TCTCGC";"TCTCGG";"TCTGAA";"TCTGAT";"TCTGAC";"TCTGAG";"TCTGTA";"TCTGTT";"TCTGTC";" ↙
TCTGTG";"TCTGCA";"TCTGCT";"TCTGCC";"TCTGCG";"TCTGGA";"TCTGGT";"TCTGGC";"TCTGGG";" ↙
TCCAAA";"TCCAAT";"TCCAAC";"TCCAAG";"TCCATA";"TCCATT";"TCCATC";"TCCATG";"TCCACA";" ↙
TCCACT";"TCCACC";"TCCACG";"TCCAGA";"TCCAGT";"TCCAGC";"TCCAGG";"TCCTAA";"TCCTAT";" ↙
TCCTAC";"TCCTAG";"TCCTTA";"TCCTTT";"TCCTTC";"TCCTTG";"TCCTCA";"TCCTCT";"TCCTCC";" ↙
TCCTCG";"TCCTGA";"TCCTGT";"TCCTGC";"TCCTGG";"TCCCAA";"TCCCAT";"TCCCAC";"TCCCAG";" ↙
TCCCTA";"TCCCTT";"TCCCTC";"TCCCTG";"TCCCCA";"TCCCCT";"TCCCCC";"TCCCCG";"TCCCGA";" ↙
TCCCGT";"TCCCGC";"TCCCGG";"TCCGAA";"TCCGAT";"TCCGAC";"TCCGAG";"TCCGTA";"TCCGTT";" ↙
TCCGTC";"TCCGTG";"TCCGCA";"TCCGCT";"TCCGCC";"TCCGCG";"TCCGGA";"TCCGGT";"TCCGGC";" ↙
TCCGGG";"TCGAAA";"TCGAAT";"TCGAAC";"TCGAAG";"TCGATA";"TCGATT";"TCGATC";"TCGATG";" ↙
TCGACA";"TCGACT";"TCGACC";"TCGACG";"TCGAGA";"TCGAGT";"TCGAGC";"TCGAGG";"TCGTAA";" ↙

```
TCGTAT";"TCGTAC";"TCGTAG";"TCGTTA";"TCGTTT";"TCGTTC";"TCGTTG";"TCGTCA";"TCGTCT";" ↙
TCGTCC";"TCGTCG";"TCGTGA";"TCGTGT";"TCGTGC";"TCGTGG";"TCGCAA";"TCGCAT";"TCGCAC";" ↙
TCGCAG";"TCGCTA";"TCGCTT";"TCGCTC";"TCGCTG";"TCGCCA";"TCGCCT";"TCGCCC";"TCGCCG";" ↙
TCGCGA";"TCGCGT";"TCGCGC";"TCGCGG";"TCGGAA";"TCGGAT";"TCGGAC";"TCGGAG";"TCGGTA";" ↙
TCGGTT";"TCGGTC";"TCGGTG";"TCGGCA";"TCGGCT";"TCGGCC";"TCGGCG";"TCGGGA";"TCGGGT";" ↙
TCGGGC";"TCGGGG";"TGAAAA";"TGAAAT";"TGAAAC";"TGAAAG";"TGAATA";"TGAATT";"TGAATC";" ↙
TGAATG";"TGAACA";"TGAACT";"TGAACC";"TGAACG";"TGAAGA";"TGAAGT";"TGAAGC";"TGAAGG";" ↙
TGATAA";"TGATAT";"TGATAC";"TGATAG";"TGATTA";"TGATTT";"TGATTC";"TGATTG";"TGATCA";" ↙
TGATCT";"TGATCC";"TGATCG";"TGATGA";"TGATGT";"TGATGC";"TGATGG";"TGACAA";"TGACAT";" ↙
TGACAC";"TGACAG";"TGACTA";"TGACTT";"TGACTC";"TGACTG";"TGACCA";"TGACCT";"TGACCC";" ↙
TGACCG";"TGACGA";"TGACGT";"TGACGC";"TGACGG";"TGAGAA";"TGAGAT";"TGAGAC";"TGAGAG";" ↙
TGAGTA";"TGAGTT";"TGAGTC";"TGAGTG";"TGAGCA";"TGAGCT";"TGAGCC";"TGAGCG";"TGAGGA";" ↙
TGAGGT";"TGAGGC";"TGAGGG";"TGTAAA";"TGTAAT";"TGTAAC";"TGTAAG";"TGTATA";"TGTATT";" ↙
TGTATC";"TGTATG";"TGTACA";"TGTACT";"TGTACC";"TGTACG";"TGTAGA";"TGTAGT";"TGTAGC";" ↙
TGTAGG";"TGTTAA";"TGTTAT";"TGTTAC";"TGTTAG";"TGTTTA";"TGTTTT";"TGTTTC";"TGTTTG";" ↙
TGTTCA";"TGTTCT";"TGTTCC";"TGTTCG";"TGTTGA";"TGTTGT";"TGTTGC";"TGTTGG";"TGTCAA";" ↙
TGTCAT";"TGTCAC";"TGTCAG";"TGTCTA";"TGTCTT";"TGTCTC";"TGTCTG";"TGTCCA";"TGTCCT";" ↙
TGTCCC";"TGTCCG";"TGTCGA";"TGTCGT";"TGTCGC";"TGTCGG";"TGTGAA";"TGTGAT";"TGTGAC";" ↙
TGTGAG";"TGTGTA";"TGTGTT";"TGTGTC";"TGTGTG";"TGTGCA";"TGTGCT";"TGTGCC";"TGTGCG";" ↙
TGTGGA";"TGTGGT";"TGTGGC";"TGTGGG";"TGCAAA";"TGCAAT";"TGCAAC";"TGCAAG";"TGCATA";" ↙
TGCATT";"TGCATC";"TGCATG";"TGCACA";"TGCACT";"TGCACC";"TGCACG";"TGCAGA";"TGCAGT";" ↙
TGCAGC";"TGCAGG";"TGCTAA";"TGCTAT";"TGCTAC";"TGCTAG";"TGCTTA";"TGCTTT";"TGCTTC";" ↙
TGCTTG";"TGCTCA";"TGCTCT";"TGCTCC";"TGCTCG";"TGCTGA";"TGCTGT";"TGCTGC";"TGCTGG";" ↙
TGCCAA";"TGCCAT";"TGCCAC";"TGCCAG";"TGCCTA";"TGCCTT";"TGCCTC";"TGCCTG";"TGCCCA";" ↙
TGCCCT";"TGCCCC";"TGCCCG";"TGCCGA";"TGCCGT";"TGCCGC";"TGCCGG";"TGCGAA";"TGCGAT";" ↙
TGCGAC";"TGCGAG";"TGCGTA";"TGCGTT";"TGCGTC";"TGCGTG";"TGCGCA";"TGCGCT";"TGCGCC";" ↙
TGCGCG";"TGCGGA";"TGCGGT";"TGCGGC";"TGCGGG";"TGGAAA";"TGGAAT";"TGGAAC";"TGGAAG";" ↙
TGGATA";"TGGATT";"TGGATC";"TGGATG";"TGGACA";"TGGACT";"TGGACC";"TGGACG";"TGGAGA";" ↙
TGGAGT";"TGGAGC";"TGGAGG";"TGGTAA";"TGGTAT";"TGGTAC";"TGGTAG";"TGGTTA";"TGGTTT";" ↙
TGGTTC";"TGGTTG";"TGGTCA";"TGGTCT";"TGGTCC";"TGGTCG";"TGGTGA";"TGGTGT";"TGGTGC";" ↙
TGGTGG";"TGGCAA";"TGGCAT";"TGGCAC";"TGGCAG";"TGGCTA";"TGGCTT";"TGGCTC";"TGGCTG";" ↙
TGGCCA";"TGGCCT";"TGGCCC";"TGGCCG";"TGGCGA";"TGGCGT";"TGGCGC";"TGGCGG";"TGGGAA";" ↙
TGGGAT";"TGGGAC";"TGGGAG";"TGGGTA";"TGGGTT";"TGGGTC";"TGGGTG";"TGGGCA";"TGGGCT";" ↙
TGGGCC";"TGGGCG";"TGGGGA";"TGGGGT";"TGGGGC";"TGGGGG";"CAAAAA";"CAAAAT";"CAAAAC";" ↙
CAAAAG";"CAAATA";"CAAATT";"CAAATC";"CAAATG";"CAAACA";"CAAACT";"CAAACC";"CAAACG";" ↙
CAAAGA";"CAAAGT";"CAAAGC";"CAAAGG";"CAATAA";"CAATAT";"CAATAC";"CAATAG";"CAATTA";" ↙
CAATTT";"CAATTC";"CAATTG";"CAATCA";"CAATCT";"CAATCC";"CAATCG";"CAATGA";"CAATGT";" ↙
CAATGC";"CAATGG";"CAACAA";"CAACAT";"CAACAC";"CAACAG";"CAACTA";"CAACTT";"CAACTC";" ↙
CAACTG";"CAACCA";"CAACCT";"CAACCC";"CAACCG";"CAACGA";"CAACGT";"CAACGC";"CAACGG";" ↙
CAAGAA";"CAAGAT";"CAAGAC";"CAAGAG";"CAAGTA";"CAAGTT";"CAAGTC";"CAAGTG";"CAAGCA";" ↙
CAAGCT";"CAAGCC";"CAAGCG";"CAAGGA";"CAAGGT";"CAAGGC";"CAAGGG";"CATAAA";"CATAAT";" ↙
CATAAC";"CATAAG";"CATATA";"CATATT";"CATATC";"CATATG";"CATACA";"CATACT";"CATACC";" ↙
CATACG";"CATAGA";"CATAGT";"CATAGC";"CATAGG";"CATTAA";"CATTAT";"CATTAC";"CATTAG";" ↙
CATTTA";"CATTTT";"CATTTC";"CATTTG";"CATTCA";"CATTCT";"CATTCC";"CATTCG";"CATTGA";" ↙
CATTGT";"CATTGC";"CATTGG";"CATCAA";"CATCAT";"CATCAC";"CATCAG";"CATCTA";"CATCTT";" ↙
CATCTC";"CATCTG";"CATCCA";"CATCCT";"CATCCC";"CATCCG";"CATCGA";"CATCGT";"CATCGC";" ↙
CATCGG";"CATGAA";"CATGAT";"CATGAC";"CATGAG";"CATGTA";"CATGTT";"CATGTC";"CATGTG";" ↙
CATGCA";"CATGCT";"CATGCC";"CATGCG";"CATGGA";"CATGGT";"CATGGC";"CATGGG";"CACAAA";" ↙
CACAAT";"CACAAC";"CACAAG";"CACATA";"CACATT";"CACATC";"CACATG";"CACACA";"CACACT";" ↙
CACACC";"CACACG";"CACAGA";"CACAGT";"CACAGC";"CACAGG";"CACTAA";"CACTAT";"CACTAC";" ↙
CACTAG";"CACTTA";"CACTTT";"CACTTC";"CACTTG";"CACTCA";"CACTCT";"CACTCC";"CACTCG";" ↙
CACTGA";"CACTGT";"CACTGC";"CACTGG";"CACCAA";"CACCAT";"CACCAC";"CACCAG";"CACCTA";" ↙
CACCTT";"CACCTC";"CACCTG";"CACCCA";"CACCCT";"CACCCC";"CACCCG";"CACCGA";"CACCGT";" ↙
CACCGC";"CACCGG";"CACGAA";"CACGAT";"CACGAC";"CACGAG";"CACGTA";"CACGTT";"CACGTC";" ↙
CACGTG";"CACGCA";"CACGCT";"CACGCC";"CACGCG";"CACGGA";"CACGGT";"CACGGC";"CACGGG";" ↙
CAGAAA";"CAGAAT";"CAGAAC";"CAGAAG";"CAGATA";"CAGATT";"CAGATC";"CAGATG";"CAGACA";" ↙
```

CAGACT";"CAGACC";"CAGACG";"CAGAGA";"CAGAGT";"CAGAGC";"CAGAGG";"CAGTAA";"CAGTAT";" ↙
CAGTAC";"CAGTAG";"CAGTTA";"CAGTTT";"CAGTTC";"CAGTTG";"CAGTCA";"CAGTCT";"CAGTCC";" ↙
CAGTCG";"CAGTGA";"CAGTGT";"CAGTGC";"CAGTGG";"CAGCAA";"CAGCAT";"CAGCAC";"CAGCAG";" ↙
CAGCTA";"CAGCTT";"CAGCTC";"CAGCTG";"CAGCCA";"CAGCCT";"CAGCCC";"CAGCCG";"CAGCGA";" ↙
CAGCGT";"CAGCGC";"CAGCGG";"CAGGAA";"CAGGAT";"CAGGAC";"CAGGAG";"CAGGTA";"CAGGTT";" ↙
CAGGTC";"CAGGTG";"CAGGCA";"CAGGCT";"CAGGCC";"CAGGCG";"CAGGGA";"CAGGGT";"CAGGGC";" ↙
CAGGGG";"CTAAAA";"CTAAAT";"CTAAAC";"CTAAAG";"CTAATA";"CTAATT";"CTAATC";"CTAATG";" ↙
CTAACA";"CTAACT";"CTAACC";"CTAACG";"CTAAGA";"CTAAGT";"CTAAGC";"CTAAGG";"CTATAA";" ↙
CTATAT";"CTATAC";"CTATAG";"CTATTA";"CTATTT";"CTATTC";"CTATTG";"CTATCA";"CTATCT";" ↙
CTATCC";"CTATCG";"CTATGA";"CTATGT";"CTATGC";"CTATGG";"CTACAA";"CTACAT";"CTACAC";" ↙
CTACAG";"CTACTA";"CTACTT";"CTACTC";"CTACTG";"CTACCA";"CTACCT";"CTACCC";"CTACCG";" ↙
CTACGA";"CTACGT";"CTACGC";"CTACGG";"CTAGAA";"CTAGAT";"CTAGAC";"CTAGAG";"CTAGTA";" ↙
CTAGTT";"CTAGTC";"CTAGTG";"CTAGCA";"CTAGCT";"CTAGCC";"CTAGCG";"CTAGGA";"CTAGGT";" ↙
CTAGGC";"CTAGGG";"CTTAAA";"CTTAAT";"CTTAAC";"CTTAAG";"CTTATA";"CTTATT";"CTTATC";" ↙
CTTATG";"CTTACA";"CTTACT";"CTTACC";"CTTACG";"CTTAGA";"CTTAGT";"CTTAGC";"CTTAGG";" ↙
CTTTAA";"CTTTAT";"CTTTAC";"CTTTAG";"CTTTTA";"CTTTTT";"CTTTTC";"CTTTTG";"CTTTCA";" ↙
CTTTCT";"CTTTCC";"CTTTCG";"CTTTGA";"CTTTGT";"CTTTGC";"CTTTGG";"CTTCAA";"CTTCAT";" ↙
CTTCAC";"CTTCAG";"CTTCTA";"CTTCTT";"CTTCTC";"CTTCTG";"CTTCCA";"CTTCCT";"CTTCCC";" ↙
CTTCCG";"CTTCGA";"CTTCGT";"CTTCGC";"CTTCGG";"CTTGAA";"CTTGAT";"CTTGAC";"CTTGAG";" ↙
CTTGTA";"CTTGTT";"CTTGTC";"CTTGTG";"CTTGCA";"CTTGCT";"CTTGCC";"CTTGCG";"CTTGGA";" ↙
CTTGGT";"CTTGGC";"CTTGGG";"CTCAAA";"CTCAAT";"CTCAAC";"CTCAAG";"CTCATA";"CTCATT";" ↙
CTCATC";"CTCATG";"CTCACA";"CTCACT";"CTCACC";"CTCACG";"CTCAGA";"CTCAGT";"CTCAGC";" ↙
CTCAGG";"CTCTAA";"CTCTAT";"CTCTAC";"CTCTAG";"CTCTTA";"CTCTTT";"CTCTTC";"CTCTTG";" ↙
CTCTCA";"CTCTCT";"CTCTCC";"CTCTCG";"CTCTGA";"CTCTGT";"CTCTGC";"CTCTGG";"CTCCAA";" ↙
CTCCAT";"CTCCAC";"CTCCAG";"CTCCTA";"CTCCTT";"CTCCTC";"CTCCTG";"CTCCCA";"CTCCCT";" ↙
CTCCCC";"CTCCCG";"CTCCGA";"CTCCGT";"CTCCGC";"CTCCGG";"CTCGAA";"CTCGAT";"CTCGAC";" ↙
CTCGAG";"CTCGTA";"CTCGTT";"CTCGTC";"CTCGTG";"CTCGCA";"CTCGCT";"CTCGCC";"CTCGCG";" ↙
CTCGGA";"CTCGGT";"CTCGGC";"CTCGGG";"CTGAAA";"CTGAAT";"CTGAAC";"CTGAAG";"CTGATA";" ↙
CTGATT";"CTGATC";"CTGATG";"CTGACA";"CTGACT";"CTGACC";"CTGACG";"CTGAGA";"CTGAGT";" ↙
CTGAGC";"CTGAGG";"CTGTAA";"CTGTAT";"CTGTAC";"CTGTAG";"CTGTTA";"CTGTTT";"CTGTTC";" ↙
CTGTTG";"CTGTCA";"CTGTCT";"CTGTCC";"CTGTCG";"CTGTGA";"CTGTGT";"CTGTGC";"CTGTGG";" ↙
CTGCAA";"CTGCAT";"CTGCAC";"CTGCAG";"CTGCTA";"CTGCTT";"CTGCTC";"CTGCTG";"CTGCCA";" ↙
CTGCCT";"CTGCCC";"CTGCCG";"CTGCGA";"CTGCGT";"CTGCGC";"CTGCGG";"CTGGAA";"CTGGAT";" ↙
CTGGAC";"CTGGAG";"CTGGTA";"CTGGTT";"CTGGTC";"CTGGTG";"CTGGCA";"CTGGCT";"CTGGCC";" ↙
CTGGCG";"CTGGGA";"CTGGGT";"CTGGGC";"CTGGGG";"CCAAAA";"CCAAAT";"CCAAAC";"CCAAAG";" ↙
CCAATA";"CCAATT";"CCAATC";"CCAATG";"CCAACA";"CCAACT";"CCAACC";"CCAACG";"CCAAGA";" ↙
CCAAGT";"CCAAGC";"CCAAGG";"CCATAA";"CCATAT";"CCATAC";"CCATAG";"CCATTA";"CCATTT";" ↙
CCATTC";"CCATTG";"CCATCA";"CCATCT";"CCATCC";"CCATCG";"CCATGA";"CCATGT";"CCATGC";" ↙
CCATGG";"CCACAA";"CCACAT";"CCACAC";"CCACAG";"CCACTA";"CCACTT";"CCACTC";"CCACTG";" ↙
CCACCA";"CCACCT";"CCACCC";"CCACCG";"CCACGA";"CCACGT";"CCACGC";"CCACGG";"CCAGAA";" ↙
CCAGAT";"CCAGAC";"CCAGAG";"CCAGTA";"CCAGTT";"CCAGTC";"CCAGTG";"CCAGCA";"CCAGCT";" ↙
CCAGCC";"CCAGCG";"CCAGGA";"CCAGGT";"CCAGGC";"CCAGGG";"CCTAAA";"CCTAAT";"CCTAAC";" ↙
CCTAAG";"CCTATA";"CCTATT";"CCTATC";"CCTATG";"CCTACA";"CCTACT";"CCTACC";"CCTACG";" ↙
CCTAGA";"CCTAGT";"CCTAGC";"CCTAGG";"CCTTAA";"CCTTAT";"CCTTAC";"CCTTAG";"CCTTTA";" ↙
CCTTTT";"CCTTTC";"CCTTTG";"CCTTCA";"CCTTCT";"CCTTCC";"CCTTCG";"CCTTGA";"CCTTGT";" ↙
CCTTGC";"CCTTGG";"CCTCAA";"CCTCAT";"CCTCAC";"CCTCAG";"CCTCTA";"CCTCTT";"CCTCTC";" ↙
CCTCTG";"CCTCCA";"CCTCCT";"CCTCCC";"CCTCCG";"CCTCGA";"CCTCGT";"CCTCGC";"CCTCGG";" ↙
CCTGAA";"CCTGAT";"CCTGAC";"CCTGAG";"CCTGTA";"CCTGTT";"CCTGTC";"CCTGTG";"CCTGCA";" ↙
CCTGCT";"CCTGCC";"CCTGCG";"CCTGGA";"CCTGGT";"CCTGGC";"CCTGGG";"CCCAAA";"CCCAAT";" ↙
CCCAAC";"CCCAAG";"CCCATA";"CCCATT";"CCCATC";"CCCATG";"CCCACA";"CCCACT";"CCCACC";" ↙
CCCACG";"CCCAGA";"CCCAGT";"CCCAGC";"CCCAGG";"CCCTAA";"CCCTAT";"CCCTAC";"CCCTAG";" ↙
CCCTTA";"CCCTTT";"CCCTTC";"CCCTTG";"CCCTCA";"CCCTCT";"CCCTCC";"CCCTCG";"CCCTGA";" ↙
CCCTGT";"CCCTGC";"CCCTGG";"CCCCAA";"CCCCAT";"CCCCAC";"CCCCAG";"CCCCTA";"CCCCTT";" ↙
CCCCTC";"CCCCTG";"CCCCCA";"CCCCCT";"CCCCCC";"CCCCCG";"CCCCGA";"CCCCGT";"CCCCGC";" ↙
CCCCGG";"CCCGAA";"CCCGAT";"CCCGAC";"CCCGAG";"CCCGTA";"CCCGTT";"CCCGTC";"CCCGTG";" ↙
CCCGCA";"CCCGCT";"CCCGCC";"CCCGCG";"CCCGGA";"CCCGGT";"CCCGGC";"CCCGGG";"CCGAAA";" ↙

CCGAAT";"CCGAAC";"CCGAAG";"CCGATA";"CCGATT";"CCGATC";"CCGATG";"CCGACA";"CCGACT";" ↙
CCGACC";"CCGACG";"CCGAGA";"CCGAGT";"CCGAGC";"CCGAGG";"CCGTAA";"CCGTAT";"CCGTAC";" ↙
CCGTAG";"CCGTTA";"CCGTTT";"CCGTTC";"CCGTTG";"CCGTCA";"CCGTCT";"CCGTCC";"CCGTCG";" ↙
CCGTGA";"CCGTGT";"CCGTGC";"CCGTGG";"CCGCAA";"CCGCAT";"CCGCAC";"CCGCAG";"CCGCTA";" ↙
CCGCTT";"CCGCTC";"CCGCTG";"CCGCCA";"CCGCCT";"CCGCCC";"CCGCCG";"CCGCGA";"CCGCGT";" ↙
CCGCGC";"CCGCGG";"CCGGAA";"CCGGAT";"CCGGAC";"CCGGAG";"CCGGTA";"CCGGTT";"CCGGTC";" ↙
CCGGTG";"CCGGCA";"CCGGCT";"CCGGCC";"CCGGCG";"CCGGGA";"CCGGGT";"CCGGGC";"CCGGGG";" ↙
CGAAAA";"CGAAAT";"CGAAAC";"CGAAAG";"CGAATA";"CGAATT";"CGAATC";"CGAATG";"CGAACA";" ↙
CGAACT";"CGAACC";"CGAACG";"CGAAGA";"CGAAGT";"CGAAGC";"CGAAGG";"CGATAA";"CGATAT";" ↙
CGATAC";"CGATAG";"CGATTA";"CGATTT";"CGATTC";"CGATTG";"CGATCA";"CGATCT";"CGATCC";" ↙
CGATCG";"CGATGA";"CGATGT";"CGATGC";"CGATGG";"CGACAA";"CGACAT";"CGACAC";"CGACAG";" ↙
CGACTA";"CGACTT";"CGACTC";"CGACTG";"CGACCA";"CGACCT";"CGACCC";"CGACCG";"CGACGA";" ↙
CGACGT";"CGACGC";"CGACGG";"CGAGAA";"CGAGAT";"CGAGAC";"CGAGAG";"CGAGTA";"CGAGTT";" ↙
CGAGTC";"CGAGTG";"CGAGCA";"CGAGCT";"CGAGCC";"CGAGCG";"CGAGGA";"CGAGGT";"CGAGGC";" ↙
CGAGGG";"CGTAAA";"CGTAAT";"CGTAAC";"CGTAAG";"CGTATA";"CGTATT";"CGTATC";"CGTATG";" ↙
CGTACA";"CGTACT";"CGTACC";"CGTACG";"CGTAGA";"CGTAGT";"CGTAGC";"CGTAGG";"CGTTAA";" ↙
CGTTAT";"CGTTAC";"CGTTAG";"CGTTTA";"CGTTTT";"CGTTTC";"CGTTTG";"CGTTCA";"CGTTCT";" ↙
CGTTCC";"CGTTCG";"CGTTGA";"CGTTGT";"CGTTGC";"CGTTGG";"CGTCAA";"CGTCAT";"CGTCAC";" ↙
CGTCAG";"CGTCTA";"CGTCTT";"CGTCTC";"CGTCTG";"CGTCCA";"CGTCCT";"CGTCCC";"CGTCCG";" ↙
CGTCGA";"CGTCGT";"CGTCGC";"CGTCGG";"CGTGAA";"CGTGAT";"CGTGAC";"CGTGAG";"CGTGTA";" ↙
CGTGTT";"CGTGTC";"CGTGTG";"CGTGCA";"CGTGCT";"CGTGCC";"CGTGCG";"CGTGGA";"CGTGGT";" ↙
CGTGGC";"CGTGGG";"CGCAAA";"CGCAAT";"CGCAAC";"CGCAAG";"CGCATA";"CGCATT";"CGCATC";" ↙
CGCATG";"CGCACA";"CGCACT";"CGCACC";"CGCACG";"CGCAGA";"CGCAGT";"CGCAGC";"CGCAGG";" ↙
CGCTAA";"CGCTAT";"CGCTAC";"CGCTAG";"CGCTTA";"CGCTTT";"CGCTTC";"CGCTTG";"CGCTCA";" ↙
CGCTCT";"CGCTCC";"CGCTCG";"CGCTGA";"CGCTGT";"CGCTGC";"CGCTGG";"CGCCAA";"CGCCAT";" ↙
CGCCAC";"CGCCAG";"CGCCTA";"CGCCTT";"CGCCTC";"CGCCTG";"CGCCCA";"CGCCCT";"CGCCCC";" ↙
CGCCCG";"CGCCGA";"CGCCGT";"CGCCGC";"CGCCGG";"CGCGAA";"CGCGAT";"CGCGAC";"CGCGAG";" ↙
CGCGTA";"CGCGTT";"CGCGTC";"CGCGTG";"CGCGCA";"CGCGCT";"CGCGCC";"CGCGCG";"CGCGGA";" ↙
CGCGGT";"CGCGGC";"CGCGGG";"CGGAAA";"CGGAAT";"CGGAAC";"CGGAAG";"CGGATA";"CGGATT";" ↙
CGGATC";"CGGATG";"CGGACA";"CGGACT";"CGGACC";"CGGACG";"CGGAGA";"CGGAGT";"CGGAGC";" ↙
CGGAGG";"CGGTAA";"CGGTAT";"CGGTAC";"CGGTAG";"CGGTTA";"CGGTTT";"CGGTTC";"CGGTTG";" ↙
CGGTCA";"CGGTCT";"CGGTCC";"CGGTCG";"CGGTGA";"CGGTGT";"CGGTGC";"CGGTGG";"CGGCAA";" ↙
CGGCAT";"CGGCAC";"CGGCAG";"CGGCTA";"CGGCTT";"CGGCTC";"CGGCTG";"CGGCCA";"CGGCCT";" ↙
CGGCCC";"CGGCCG";"CGGCGA";"CGGCGT";"CGGCGC";"CGGCGG";"CGGGAA";"CGGGAT";"CGGGAC";" ↙
CGGGAG";"CGGGTA";"CGGGTT";"CGGGTC";"CGGGTG";"CGGGCA";"CGGGCT";"CGGGCC";"CGGGCG";" ↙
CGGGGA";"CGGGGT";"CGGGGC";"CGGGGG";"GAAAAA";"GAAAAT";"GAAAAC";"GAAAAG";"GAAATA";" ↙
GAAATT";"GAAATC";"GAAATG";"GAAACA";"GAAACT";"GAAACC";"GAAACG";"GAAAGA";"GAAAGT";" ↙
GAAAGC";"GAAAGG";"GAATAA";"GAATAT";"GAATAC";"GAATAG";"GAATTA";"GAATTT";"GAATTC";" ↙
GAATTG";"GAATCA";"GAATCT";"GAATCC";"GAATCG";"GAATGA";"GAATGT";"GAATGC";"GAATGG";" ↙
GAACAA";"GAACAT";"GAACAC";"GAACAG";"GAACTA";"GAACTT";"GAACTC";"GAACTG";"GAACCA";" ↙
GAACCT";"GAACCC";"GAACCG";"GAACGA";"GAACGT";"GAACGC";"GAACGG";"GAAGAA";"GAAGAT";" ↙
GAAGAC";"GAAGAG";"GAAGTA";"GAAGTT";"GAAGTC";"GAAGTG";"GAAGCA";"GAAGCT";"GAAGCC";" ↙
GAAGCG";"GAAGGA";"GAAGGT";"GAAGGC";"GAAGGG";"GATAAA";"GATAAT";"GATAAC";"GATAAG";" ↙
GATATA";"GATATT";"GATATC";"GATATG";"GATACA";"GATACT";"GATACC";"GATACG";"GATAGA";" ↙
GATAGT";"GATAGC";"GATAGG";"GATTAA";"GATTAT";"GATTAC";"GATTAG";"GATTTA";"GATTTT";" ↙
GATTTC";"GATTTG";"GATTCA";"GATTCT";"GATTCC";"GATTCG";"GATTGA";"GATTGT";"GATTGC";" ↙
GATTGG";"GATCAA";"GATCAT";"GATCAC";"GATCAG";"GATCTA";"GATCTT";"GATCTC";"GATCTG";" ↙
GATCCA";"GATCCT";"GATCCC";"GATCCG";"GATCGA";"GATCGT";"GATCGC";"GATCGG";"GATGAA";" ↙
GATGAT";"GATGAC";"GATGAG";"GATGTA";"GATGTT";"GATGTC";"GATGTG";"GATGCA";"GATGCT";" ↙
GATGCC";"GATGCG";"GATGGA";"GATGGT";"GATGGC";"GATGGG";"GACAAA";"GACAAT";"GACAAC";" ↙
GACAAG";"GACATA";"GACATT";"GACATC";"GACATG";"GACACA";"GACACT";"GACACC";"GACACG";" ↙
GACAGA";"GACAGT";"GACAGC";"GACAGG";"GACTAA";"GACTAT";"GACTAC";"GACTAG";"GACTTA";" ↙
GACTTT";"GACTTC";"GACTTG";"GACTCA";"GACTCT";"GACTCC";"GACTCG";"GACTGA";"GACTGT";" ↙
GACTGC";"GACTGG";"GACCAA";"GACCAT";"GACCAC";"GACCAG";"GACCTA";"GACCTT";"GACCTC";" ↙
GACCTG";"GACCCA";"GACCCT";"GACCCC";"GACCCG";"GACCGA";"GACCGT";"GACCGC";"GACCGG";" ↙
GACGAA";"GACGAT";"GACGAC";"GACGAG";"GACGTA";"GACGTT";"GACGTC";"GACGTG";"GACGCA";" ↙

GACGCT";"GACGCC";"GACGCG";"GACGGA";"GACGGT";"GACGGC";"GACGGG";"GAGAAA";"GAGAAT";" ↙
GAGAAC";"GAGAAG";"GAGATA";"GAGATT";"GAGATC";"GAGATG";"GAGACA";"GAGACT";"GAGACC";" ↙
GAGACG";"GAGAGA";"GAGAGT";"GAGAGC";"GAGAGG";"GAGTAA";"GAGTAT";"GAGTAC";"GAGTAG";" ↙
GAGTTA";"GAGTTT";"GAGTTC";"GAGTTG";"GAGTCA";"GAGTCT";"GAGTCC";"GAGTCG";"GAGTGA";" ↙
GAGTGT";"GAGTGC";"GAGTGG";"GAGCAA";"GAGCAT";"GAGCAC";"GAGCAG";"GAGCTA";"GAGCTT";" ↙
GAGCTC";"GAGCTG";"GAGCCA";"GAGCCT";"GAGCCC";"GAGCCG";"GAGCGA";"GAGCGT";"GAGCGC";" ↙
GAGCGG";"GAGGAA";"GAGGAT";"GAGGAC";"GAGGAG";"GAGGTA";"GAGGTT";"GAGGTC";"GAGGTG";" ↙
GAGGCA";"GAGGCT";"GAGGCC";"GAGGCG";"GAGGGA";"GAGGGT";"GAGGGC";"GAGGGG";"GTAAAA";" ↙
GTAAAT";"GTAAAC";"GTAAAG";"GTAATA";"GTAATT";"GTAATC";"GTAATG";"GTAACA";"GTAACT";" ↙
GTAACC";"GTAACG";"GTAAGA";"GTAAGT";"GTAAGC";"GTAAGG";"GTATAA";"GTATAT";"GTATAC";" ↙
GTATAG";"GTATTA";"GTATTT";"GTATTC";"GTATTG";"GTATCA";"GTATCT";"GTATCC";"GTATCG";" ↙
GTATGA";"GTATGT";"GTATGC";"GTATGG";"GTACAA";"GTACAT";"GTACAC";"GTACAG";"GTACTA";" ↙
GTACTT";"GTACTC";"GTACTG";"GTACCA";"GTACCT";"GTACCC";"GTACCG";"GTACGA";"GTACGT";" ↙
GTACGC";"GTACGG";"GTAGAA";"GTAGAT";"GTAGAC";"GTAGAG";"GTAGTA";"GTAGTT";"GTAGTC";" ↙
GTAGTG";"GTAGCA";"GTAGCT";"GTAGCC";"GTAGCG";"GTAGGA";"GTAGGT";"GTAGGC";"GTAGGG";" ↙
GTTAAA";"GTTAAT";"GTTAAC";"GTTAAG";"GTTATA";"GTTATT";"GTTATC";"GTTATG";"GTTACA";" ↙
GTTACT";"GTTACC";"GTTACG";"GTTAGA";"GTTAGT";"GTTAGC";"GTTAGG";"GTTTAA";"GTTTAT";" ↙
GTTTAC";"GTTTAG";"GTTTTA";"GTTTTT";"GTTTTC";"GTTTTG";"GTTTCA";"GTTTCT";"GTTTCC";" ↙
GTTTCG";"GTTTGA";"GTTTGT";"GTTTGC";"GTTTGG";"GTTCAA";"GTTCAT";"GTTCAC";"GTTCAG";" ↙
GTTCTA";"GTTCTT";"GTTCTC";"GTTCTG";"GTTCCA";"GTTCCT";"GTTCCC";"GTTCCG";"GTTCGA";" ↙
GTTCGT";"GTTCGC";"GTTCGG";"GTTGAA";"GTTGAT";"GTTGAC";"GTTGAG";"GTTGTA";"GTTGTT";" ↙
GTTGTC";"GTTGTG";"GTTGCA";"GTTGCT";"GTTGCC";"GTTGCG";"GTTGGA";"GTTGGT";"GTTGGC";" ↙
GTTGGG";"GTCAAA";"GTCAAT";"GTCAAC";"GTCAAG";"GTCATA";"GTCATT";"GTCATC";"GTCATG";" ↙
GTCACA";"GTCACT";"GTCACC";"GTCACG";"GTCAGA";"GTCAGT";"GTCAGC";"GTCAGG";"GTCTAA";" ↙
GTCTAT";"GTCTAC";"GTCTAG";"GTCTTA";"GTCTTT";"GTCTTC";"GTCTTG";"GTCTCA";"GTCTCT";" ↙
GTCTCC";"GTCTCG";"GTCTGA";"GTCTGT";"GTCTGC";"GTCTGG";"GTCCAA";"GTCCAT";"GTCCAC";" ↙
GTCCAG";"GTCCTA";"GTCCTT";"GTCCTC";"GTCCTG";"GTCCCA";"GTCCCT";"GTCCCC";"GTCCCG";" ↙
GTCCGA";"GTCCGT";"GTCCGC";"GTCCGG";"GTCGAA";"GTCGAT";"GTCGAC";"GTCGAG";"GTCGTA";" ↙
GTCGTT";"GTCGTC";"GTCGTG";"GTCGCA";"GTCGCT";"GTCGCC";"GTCGCG";"GTCGGA";"GTCGGT";" ↙
GTCGGC";"GTCGGG";"GTGAAA";"GTGAAT";"GTGAAC";"GTGAAG";"GTGATA";"GTGATT";"GTGATC";" ↙
GTGATG";"GTGACA";"GTGACT";"GTGACC";"GTGACG";"GTGAGA";"GTGAGT";"GTGAGC";"GTGAGG";" ↙
GTGTAA";"GTGTAT";"GTGTAC";"GTGTAG";"GTGTTA";"GTGTTT";"GTGTTC";"GTGTTG";"GTGTCA";" ↙
GTGTCT";"GTGTCC";"GTGTCG";"GTGTGA";"GTGTGT";"GTGTGC";"GTGTGG";"GTGCAA";"GTGCAT";" ↙
GTGCAC";"GTGCAG";"GTGCTA";"GTGCTT";"GTGCTC";"GTGCTG";"GTGCCA";"GTGCCT";"GTGCCC";" ↙
GTGCCG";"GTGCGA";"GTGCGT";"GTGCGC";"GTGCGG";"GTGGAA";"GTGGAT";"GTGGAC";"GTGGAG";" ↙
GTGGTA";"GTGGTT";"GTGGTC";"GTGGTG";"GTGGCA";"GTGGCT";"GTGGCC";"GTGGCG";"GTGGGA";" ↙
GTGGGT";"GTGGGC";"GTGGGG";"GCAAAA";"GCAAAT";"GCAAAC";"GCAAAG";"GCAATA";"GCAATT";" ↙
GCAATC";"GCAATG";"GCAACA";"GCAACT";"GCAACC";"GCAACG";"GCAAGA";"GCAAGT";"GCAAGC";" ↙
GCAAGG";"GCATAA";"GCATAT";"GCATAC";"GCATAG";"GCATTA";"GCATTT";"GCATTC";"GCATTG";" ↙
GCATCA";"GCATCT";"GCATCC";"GCATCG";"GCATGA";"GCATGT";"GCATGC";"GCATGG";"GCACAA";" ↙
GCACAT";"GCACAC";"GCACAG";"GCACTA";"GCACTT";"GCACTC";"GCACTG";"GCACCA";"GCACCT";" ↙
GCACCC";"GCACCG";"GCACGA";"GCACGT";"GCACGC";"GCACGG";"GCAGAA";"GCAGAT";"GCAGAC";" ↙
GCAGAG";"GCAGTA";"GCAGTT";"GCAGTC";"GCAGTG";"GCAGCA";"GCAGCT";"GCAGCC";"GCAGCG";" ↙
GCAGGA";"GCAGGT";"GCAGGC";"GCAGGG";"GCTAAA";"GCTAAT";"GCTAAC";"GCTAAG";"GCTATA";" ↙
GCTATT";"GCTATC";"GCTATG";"GCTACA";"GCTACT";"GCTACC";"GCTACG";"GCTAGA";"GCTAGT";" ↙
GCTAGC";"GCTAGG";"GCTTAA";"GCTTAT";"GCTTAC";"GCTTAG";"GCTTTA";"GCTTTT";"GCTTTC";" ↙
GCTTTG";"GCTTCA";"GCTTCT";"GCTTCC";"GCTTCG";"GCTTGA";"GCTTGT";"GCTTGC";"GCTTGG";" ↙
GCTCAA";"GCTCAT";"GCTCAC";"GCTCAG";"GCTCTA";"GCTCTT";"GCTCTC";"GCTCTG";"GCTCCA";" ↙
GCTCCT";"GCTCCC";"GCTCCG";"GCTCGA";"GCTCGT";"GCTCGC";"GCTCGG";"GCTGAA";"GCTGAT";" ↙
GCTGAC";"GCTGAG";"GCTGTA";"GCTGTT";"GCTGTC";"GCTGTG";"GCTGCA";"GCTGCT";"GCTGCC";" ↙
GCTGCG";"GCTGGA";"GCTGGT";"GCTGGC";"GCTGGG";"GCCAAA";"GCCAAT";"GCCAAC";"GCCAAG";" ↙
GCCATA";"GCCATT";"GCCATC";"GCCATG";"GCCACA";"GCCACT";"GCCACC";"GCCACG";"GCCAGA";" ↙
GCCAGT";"GCCAGC";"GCCAGG";"GCCTAA";"GCCTAT";"GCCTAC";"GCCTAG";"GCCTTA";"GCCTTT";" ↙
GCCTTC";"GCCTTG";"GCCTCA";"GCCTCT";"GCCTCC";"GCCTCG";"GCCTGA";"GCCTGT";"GCCTGC";" ↙
GCCTGG";"GCCCAA";"GCCCAT";"GCCCAC";"GCCCAG";"GCCCTA";"GCCCTT";"GCCCTC";"GCCCTG";" ↙
GCCCCA";"GCCCCT";"GCCCCC";"GCCCCG";"GCCCGA";"GCCCGT";"GCCCGC";"GCCCGG";"GCCGAA";" ↙

```
GCCGAT";"GCCGAC";"GCCGAG";"GCCGTA";"GCCGTT";"GCCGTC";"GCCGTG";"GCCGCA";"GCCGCT";" ↙
GCCGCC";"GCCGCG";"GCCGGA";"GCCGGT";"GCCGGC";"GCCGGG";"GCGAAA";"GCGAAT";"GCGAAC";" ↙
GCGAAG";"GCGATA";"GCGATT";"GCGATC";"GCGATG";"GCGACA";"GCGACT";"GCGACC";"GCGACG";" ↙
GCGAGA";"GCGAGT";"GCGAGC";"GCGAGG";"GCGTAA";"GCGTAT";"GCGTAC";"GCGTAG";"GCGTTA";" ↙
GCGTTT";"GCGTTC";"GCGTTG";"GCGTCA";"GCGTCT";"GCGTCC";"GCGTCG";"GCGTGA";"GCGTGT";" ↙
GCGTGC";"GCGTGG";"GCGCAA";"GCGCAT";"GCGCAC";"GCGCAG";"GCGCTA";"GCGCTT";"GCGCTC";" ↙
GCGCTG";"GCGCCA";"GCGCCT";"GCGCCC";"GCGCCG";"GCGCGA";"GCGCGT";"GCGCGC";"GCGCGG";" ↙
GCGGAA";"GCGGAT";"GCGGAC";"GCGGAG";"GCGGTA";"GCGGTT";"GCGGTC";"GCGGTG";"GCGGCA";" ↙
GCGGCT";"GCGGCC";"GCGGCG";"GCGGGA";"GCGGGT";"GCGGGC";"GCGGGG";"GGAAAA";"GGAAAT";" ↙
GGAAAC";"GGAAAG";"GGAATA";"GGAATT";"GGAATC";"GGAATG";"GGAACA";"GGAACT";"GGAACC";" ↙
GGAACG";"GGAAGA";"GGAAGT";"GGAAGC";"GGAAGG";"GGATAA";"GGATAT";"GGATAC";"GGATAG";" ↙
GGATTA";"GGATTT";"GGATTC";"GGATTG";"GGATCA";"GGATCT";"GGATCC";"GGATCG";"GGATGA";" ↙
GGATGT";"GGATGC";"GGATGG";"GGACAA";"GGACAT";"GGACAC";"GGACAG";"GGACTA";"GGACTT";" ↙
GGACTC";"GGACTG";"GGACCA";"GGACCT";"GGACCC";"GGACCG";"GGACGA";"GGACGT";"GGACGC";" ↙
GGACGG";"GGAGAA";"GGAGAT";"GGAGAC";"GGAGAG";"GGAGTA";"GGAGTT";"GGAGTC";"GGAGTG";" ↙
GGAGCA";"GGAGCT";"GGAGCC";"GGAGCG";"GGAGGA";"GGAGGT";"GGAGGC";"GGAGGG";"GGTAAA";" ↙
GGTAAT";"GGTAAC";"GGTAAG";"GGTATA";"GGTATT";"GGTATC";"GGTATG";"GGTACA";"GGTACT";" ↙
GGTACC";"GGTACG";"GGTAGA";"GGTAGT";"GGTAGC";"GGTAGG";"GGTTAA";"GGTTAT";"GGTTAC";" ↙
GGTTAG";"GGTTTA";"GGTTTT";"GGTTTC";"GGTTTG";"GGTTCA";"GGTTCT";"GGTTCC";"GGTTCG";" ↙
GGTTGA";"GGTTGT";"GGTTGC";"GGTTGG";"GGTCAA";"GGTCAT";"GGTCAC";"GGTCAG";"GGTCTA";" ↙
GGTCTT";"GGTCTC";"GGTCTG";"GGTCCA";"GGTCCT";"GGTCCC";"GGTCCG";"GGTCGA";"GGTCGT";" ↙
GGTCGC";"GGTCGG";"GGTGAA";"GGTGAT";"GGTGAC";"GGTGAG";"GGTGTA";"GGTGTT";"GGTGTC";" ↙
GGTGTG";"GGTGCA";"GGTGCT";"GGTGCC";"GGTGCG";"GGTGGA";"GGTGGT";"GGTGGC";"GGTGGG";" ↙
GGCAAA";"GGCAAT";"GGCAAC";"GGCAAG";"GGCATA";"GGCATT";"GGCATC";"GGCATG";"GGCACA";" ↙
GGCACT";"GGCACC";"GGCACG";"GGCAGA";"GGCAGT";"GGCAGC";"GGCAGG";"GGCTAA";"GGCTAT";" ↙
GGCTAC";"GGCTAG";"GGCTTA";"GGCTTT";"GGCTTC";"GGCTTG";"GGCTCA";"GGCTCT";"GGCTCC";" ↙
GGCTCG";"GGCTGA";"GGCTGT";"GGCTGC";"GGCTGG";"GGCCAA";"GGCCAT";"GGCCAC";"GGCCAG";" ↙
GGCCTA";"GGCCTT";"GGCCTC";"GGCCTG";"GGCCCA";"GGCCCT";"GGCCCC";"GGCCCG";"GGCCGA";" ↙
GGCCGT";"GGCCGC";"GGCCGG";"GGCGAA";"GGCGAT";"GGCGAC";"GGCGAG";"GGCGTA";"GGCGTT";" ↙
GGCGTC";"GGCGTG";"GGCGCA";"GGCGCT";"GGCGCC";"GGCGCG";"GGCGGA";"GGCGGT";"GGCGGC";" ↙
GGCGGG";"GGGAAA";"GGGAAT";"GGGAAC";"GGGAAG";"GGGATA";"GGGATT";"GGGATC";"GGGATG";" ↙
GGGACA";"GGGACT";"GGGACC";"GGGACG";"GGGAGA";"GGGAGT";"GGGAGC";"GGGAGG";"GGGTAA";" ↙
GGGTAT";"GGGTAC";"GGGTAG";"GGGTTA";"GGGTTT";"GGGTTC";"GGGTTG";"GGGTCA";"GGGTCT";" ↙
GGGTCC";"GGGTCG";"GGGTGA";"GGGTGT";"GGGTGC";"GGGTGG";"GGGCAA";"GGGCAT";"GGGCAC";" ↙
GGGCAG";"GGGCTA";"GGGCTT";"GGGCTC";"GGGCTG";"GGGCCA";"GGGCCT";"GGGCCC";"GGGCCG";" ↙
GGGCGA";"GGGCGT";"GGGCGC";"GGGCGG";"GGGGAA";"GGGGAT";"GGGGAC";"GGGGAG";"GGGGTA";" ↙
GGGGTT";"GGGGTC";"GGGGTG";"GGGGCA";"GGGGCT";"GGGGCC";"GGGGCG";"GGGGGA";"GGGGGT";" ↙
GGGGGC";"GGGGGG"];

cont2Ind = ~ismember(allDuplets, PAMsATCG);
cont3Ind = ~ismember(allTriplets, PAMsATCG);
cont4Ind = ~ismember(all4oligo, PAMsATCG);
cont5Ind = ~ismember(all5oligo, PAMsATCG);
cont6Ind = ~ismember(all6oligo, PAMsATCG);

[c2e, c2m, c2l, ~, ~, ~] = getNumberOfPromotersContainPAMs(phageSeq, allDuplets, ↙
promoters, promotersLoc, promotersDirection, promoterLengthArray);
[c3e, c3m, c3l, ~, ~, ~] = getNumberOfPromotersContainPAMs(phageSeq, allTriplets, ↙
promoters, promotersLoc, promotersDirection, promoterLengthArray); %75[c3e, c3m, c3l, ↙
~, ~, ~] = getNumberOfPromotersContainPAMs(phageSeq, allTriplets, promoters, ↙
promotersLoc, promotersDirection, promoterLengthArray); %75
[c4e, c4m, c4l, ~, ~, ~] = getNumberOfPromotersContainPAMs(phageSeq, all4oligo, ↙
promoters, promotersLoc, promotersDirection, promoterLengthArray); %85
[c5e, c5m, c5l, ~, ~, ~] = getNumberOfPromotersContainPAMs(phageSeq, all5oligo, ↙
promoters, promotersLoc, promotersDirection, promoterLengthArray);
[c6e, c6m, c6l, ~, ~, ~] = getNumberOfPromotersContainPAMs(phageSeq, all6oligo, ↙
```

```
promoters, promotersLoc, promotersDirection, promoterLengthArray);

earlyPromotersPAMsS = sum(earlyPromotersPAMs, 2);
middlePromotersPAMsS = sum(middlePromotersPAMs, 2);
latePromotersPAMsS = sum(latePromotersPAMs, 2);
c2eS = sum(c2e, 2);
c2mS = sum(c2m, 2);
c2lS = sum(c2l, 2);
c3eS = sum(c3e, 2);
c3mS = sum(c3m, 2);
c3lS = sum(c3l, 2);
c4eS = sum(c4e, 2);
c4mS = sum(c4m, 2);
c4lS = sum(c4l, 2);
c5eS = sum(c5e, 2);
c5mS = sum(c5m, 2);
c5lS = sum(c5l, 2);
c6eS = sum(c6e, 2);
c6mS = sum(c6m, 2);
c6lS = sum(c6l, 2);

controlResults = [c2eS(cont2Ind); c2mS(cont2Ind); c2lS(cont2Ind); c3eS(cont3Ind); c3mS ↙
(cont3Ind); c3lS(cont3Ind); c4eS(cont4Ind); c4mS(cont4Ind); c4lS(cont4Ind); c5eS ↙
(cont5Ind); c5mS(cont5Ind); c5lS(cont5Ind); c6eS(cont6Ind); c6mS(cont6Ind); c6lS ↙
(cont6Ind)];
promotersResults = zeros(size(controlResults));
promotersResults(1:length(earlyPromotersPAMsS)+length(middlePromotersPAMsS)+length ↙
(latePromotersPAMsS)) = [earlyPromotersPAMsS; middlePromotersPAMsS; ↙
latePromotersPAMsS];
promotersResults(length(earlyPromotersPAMsS)+length(middlePromotersPAMsS)+length ↙
(latePromotersPAMsS)+1:length(controlResults)) = nan;
X = [[controlResults repelem(1, length(controlResults)).']; [[earlyPromotersPAMsS; ↙
middlePromotersPAMsS; latePromotersPAMsS] repelem(2, length(earlyPromotersPAMsS)+length ↙
(middlePromotersPAMsS)+length(latePromotersPAMsS)).']];
constantTerm = ones(size(X, 1), 1);
indexRand1 = randsample(1:length(controlResults), length(promotersResults));
indexRand2 = randsample(1:length(controlResults), length(promotersResults));
indexRand3 = randsample(1:length(controlResults), length(promotersResults));
indexRand4 = randsample(1:length(controlResults), length(promotersResults));

[rankP, rankH, rankStats] = ranksum(controlResults, promotersResults);
MWWStats = mwwtest([earlyPromotersPAMsS;middlePromotersPAMsS;latePromotersPAMsS].', ↙
controlResults.'); %this function is cited below, it is from file exchange for matlab, ↙
it is the same as ranksum but displays more information
% Giuseppe Cardillo (2022). mwwtest (https://github.com/dnafinder/mwwtest), GitHub. ↙
Retrieved April 20, 2022.
```
