# Supplementary material for: Bio-informatic analysis of CRISPR protospacer adjacent motifs (PAMs) in T4 genome
Source: BMC Genom Data. 2022 Jun 2;23:40. doi: 10.1186/s12863-022-01056-8 (PMC9161530; doi:10.1186/s12863-022-01056-8)
Supplement: Supplementary file 1 — Additional file 1. [file 12863_2022_1056_MOESM1_ESM.zip › getAllAAFromPAM.pdf]

```
function [listOfAA3letters, listOfAA1letter] = getAllAAFromPAM(PAM)
    nucleotidesMatchingArray = {'A', ["A", "R", "M", "W", "D", "H", "V", "N"];
                                'T', ["T", "Y", "K", "W", "B", "D", "H", "N"];
                                'C', ["C", "Y", "M", "S", "B", "H", "V", "N"];
                                'G', ["G", "R", "K", "S", "B", "D", "V", "N"]};

    codonToAminoacid = ["TTT", "Phe"; "TTC", "Phe"; "TTA", "Leu"; "TTG", "Leu";
                        "CTT", "Leu"; "CTC", "Leu"; "CTA", "Leu"; "CTG", "Leu";
                        "ATT", "Ile"; "ATC", "Ile"; "ATA", "Ile"; "ATG", "Met";
                        "GTT", "Val"; "GTC", "Val"; "GTA", "Val"; "GTG", "Val";
                        "TCT", "Ser"; "TCC", "Ser"; "TCA", "Ser"; "TCG", "Ser";
                        "CCT", "Pro"; "CCC", "Pro"; "CCA", "Pro"; "CCG", "Pro";
                        "ACT", "Thr"; "ACC", "Thr"; "ACA", "Thr"; "ACG", "Thr";
                        "GCT", "Ala"; "GCC", "Ala"; "GCA", "Ala"; "GCG", "Ala";
                        "TAT", "Tyr"; "TAC", "Tyr"; "TAA", "Stop"; "TAG", "Stop";
                        "CAT", "His"; "CAC", "His"; "CAA", "Gln"; "CAG", "Gln";
                        "AAT", "Asn"; "AAC", "Asn"; "AAA", "Lys"; "AAG", "Lys";
                        "GAT", "Asp"; "GAC", "Asp"; "GAA", "Glu"; "GAG", "Glu";
                        "TGT", "Cys"; "TGC", "Cys"; "TGA", "Stop"; "TGG", "Trp";
                        "CGT", "Arg"; "CGC", "Arg"; "CGA", "Arg"; "CGG", "Arg";
                        "AGT", "Ser"; "AGC", "Ser"; "AGA", "Arg"; "AGG", "Arg";
                        "GGT", "Gly"; "GGC", "Gly"; "GGA", "Gly"; "GGG", "Gly";];

    aaFullNames = ["A", "Ala", "alanine";
                   "R", "Arg", "arginine";
                   "N", "Asn", "asparagine";
                   "D", "Asp", "aspartic acid";
                   "C", "Cys", "cysteine";
                   "Q", "Gln", "glutamine";
                   "E", "Glu", "glutamic acid";
                   "G", "Gly", "glycine";
                   "H", "His", "histidine";
                   "I", "Ile", "isoleucine";
                   "L", "Leu", "leucine";
                   "K", "Lys", "lysine";
                   "M", "Met", "methionine";
                   "F", "Phe", "phenylalanine";
                   "P", "Pro", "proline";
                   "S", "Ser", "serine";
                   "T", "Thr", "threonine";
                   "W", "Trp", "tryptophan";
                   "Y", "Tyr", "tyrosine";
                   "V", "Val", "valine"];

%     polarityOfAminoAcid = [ "Ala", "non";
%                             "Arg", "polar"; %+ve
%                             "Asn", "polar";
%                             "Asp", "polar"; %-ve
%                             "Cys", "polar"; %special
%                             "Gln", "polar";
%                             "Glu", "polar"; %-ve
%                             "Gly", "non";   %special
%                             "His", "polar"; %+ve
%                             "Ile", "non";
%                             "Leu", "non";
%                             "Lys", "polar"; %+ve
```

```
%                              "Met", "non";
%                              "Phe", "non";
%                              "Pro", "non";   %special
%                              "Ser", "polar";
%                              "Thr", "polar";
%                              "Trp", "non";
%                              "Tyr", "polar"; %phobic
%                              "Val", "non"];

    if (strlength(PAM)<3)
       PAM = strcat('N', PAM, 'N');
    end
    codons = strings(1);
    for i=1:strlength(PAM)
        possibleNextNucleotides = strings(1);
        n = 0;
        for j=1:4
            if (sum(strcmp(nucleotidesMatchingArray{j, 2}, PAM{1}(i)))>0)
                n = n+1;
                possibleNextNucleotides(n) = nucleotidesMatchingArray{j,1};
            end
        end

        tempCodons = strings(n*length(codons), 1);
        for j=1:n
            tempCodons((j-1)*length(codons)+1:j*length(codons), 1) = strcat(codons, ↙
possibleNextNucleotides(j));
        end
        codons = tempCodons;
    end

    dividedCodons = strings;
    for i=1:length(codons)
        if (strlength(codons(i))>3)
            for j = 3:strlength(codons(i))
                dividedCodons(length(dividedCodons)+1) = codons{i}(j-2:j);
            end
        end
    end
    dividedCodons(1) = [];
    codons = unique(vertcat(dividedCodons.', codons));
    codons(strlength(codons)>3) = [];


    listOfAA3letters = strings;
    listOfAA1letter = strings;
    for i=1:length(codons)
        aa3Letters = codonToAminoacid(strcmp(codonToAminoacid(:, 1), codons(i)), 2);
        if (~strcmp(aa3Letters, "Stop"))
            if (sum(strcmp(aa3Letters, listOfAA3letters))<1)
                listOfAA3letters(length(listOfAA3letters)+1) = aa3Letters;
                listOfAA1letter(length(listOfAA1letter)+1) = aaFullNames(strcmp ↙
(aaFullNames(:,2), aa3Letters), 1);
            end
        end
    end
```

```
    listOfAA3letters(1) = [];
    listOfAA1letter(1) = [];
end
```
