# Supplementary material for: Bio-informatic analysis of CRISPR protospacer adjacent motifs (PAMs) in T4 genome
Source: BMC Genom Data. 2022 Jun 2;23:40. doi: 10.1186/s12863-022-01056-8 (PMC9161530; doi:10.1186/s12863-022-01056-8)
Supplement: Supplementary file 1 — Additional file 1. [file 12863_2022_1056_MOESM1_ESM.zip › getASA.pdf]

```
function [medianASA, meanASA, maxMedianASA, maxMeanASA] = getASA(aminoacids)
    aaSingleLetters = ["A", "R", "N", "D", "C", "Q", "E", "G", "H", "I", "L", "K", "M", ↙
"F", "P", "S", "T", "W", "Y", "V"];
    aaThreeLetters = ["ala", "arg", "asn", "asp", "cys", "gln", "glu", "gly", "his", ↙
"ile", "leu", "lys", "met", "phe", "pro", "ser", "thr", "trp", "tyr", "val"];
    medASA = [14 87 59 62 5 74 83 19 46 6 9 102 13 13 49 35 37 25 31 8];
    menASA = [27 91 62 63 15 75 81 26 56 23 26 101 36 31 51 41 44 42 43 23];

    if ( length(aminoacids(1)) == 1)
       [~, ~, ind] = intersect(aminoacids, aaSingleLetters);
    elseif ( length(aminoacids(1)) == 3)
       [~, ~, ind] = intersect(aminoacids, aaThreeLetters);
    end

    medianASA = sum(medASA(ind))/length(ind);
    meanASA = sum(menASA(ind))/length(ind);
    maxMedianASA = max(medASA(ind));
    maxMeanASA = max(menASA(ind));
end
```
