# Supplementary material for: Bio-informatic analysis of CRISPR protospacer adjacent motifs (PAMs) in T4 genome
Source: BMC Genom Data. 2022 Jun 2;23:40. doi: 10.1186/s12863-022-01056-8 (PMC9161530; doi:10.1186/s12863-022-01056-8)
Supplement: Supplementary file 1 — Additional file 1. [file 12863_2022_1056_MOESM1_ESM.zip › getGeneList.pdf]

```
gene = {"rIIA";"Pm";"rIIA.1";"Pe (1.4 -3.9)";"60";"60";"60.1";"mobA";"Pe (3.6)";"39";" ↙
Pm";"Term";"39.1";"39.2";"goF comCa";"cef mb";"motB";"Pe (5.9 -7.3)";"motB.1";"motB.2";" ↙
Pe (8.1)";"dexA";"dexA.1";"dexA.2";"dda";"dda.1";"srd";"Pe (11.5)";"modA";"modB";"Pe ↙
(12.8)";"modA.2";"modA.3";"modA.4";"srh";"mrh";"mrh.1";"mrh.2";"Pe (15.0)";"Term";" ↙
soc";"Pl";"Pl";"segF   69";"Pl";"56";"oriA";"Pm";"dam";"61   58";"Pm";"61.1";"61.2";"sp ↙
rV";"Pe (19.8)";"61.4";"dmd   61.5";"Pe (20.3)";"41";"Term";"40";"uvsX";"Pm";"segA";" ↙
Pm";"-gt";"42";"Pm";"imm";"imm.1";"Pe (26.4)";"Term";"43";"Pm";"Term";"regA";"62";" ↙
44";"Term";"45";"Pm";"rpbA";"45.2";"Pm";"46";"Pm";"46.1";"46.2";"Pe (35.3)";"47";"Pm";" ↙
47.1";"Term";"-gt";"mobB";"Pm";"Term";"-gt.2";"-gt.3";"-gt.4";"-gt.5";"55";"Pm";" ↙
55.1";"55.2";"Term";"55.3";"55.4";"Pe (40.4)";"55.5";"55.6";"Pe (41.0)";"Term";"nrdH";" ↙
55.8";"Pm";"nrdG";"Pm";"mobC";"nrdD";"I-TevII";"Pl";"nrdD";"Pm";"49";"49";"Pl";"Term";" ↙
pin";"Pe (46.7)";"49.1";"49.2";"49.3";"nrdC";"nrdC.1";"nrdC.2";"nrdC.3";"nrdC.4";"Pe ↙
(50.0)";"nrdC.5";"nrdC.6";"nrdC.7";"nrdC.8";"Pe (54.0)";"nrdC.9";"nrdC.10";"Pe ↙
(54.4)";"Term";"nrdC.11";"mobD";"mobD.1";"mobD.2";"Pe";"mobD.2a";"mobD.3";"mobD.4";" ↙
mobD.5";"Pe (57.9)";"Term";"rI.-1";"rI";"rI.1";"Pl";"tk";"tk.1";"tk.2";"tk.3";"Term";" ↙
tk.4";"vs";"vs.1";"regB";"Pe (62.2)";"vs.3";"vs.4";"vs.5";"vs.6";"vs.7";"vs.8";"denV";" ↙
Pe (64.6)";"ipII";"Pe (65.0)";"ipIII";"Pe";"e";"Pl";"Pl";"Pl";"nudE   e.1";"e.2";"e. ↙
3";"e.4";"e.5";"Term";"e.6";"Pe (69.9)";"e.7";"Pe (69.4)";"e.8";"Pe (69.8)";"Term";" ↙
rnaC";"rnaD";"tRNAR";"segB";"tRNAI";"tRNAT";"tRNAS";"tRNAP";"tRNAG";"tRNAL";"tRNAE";" ↙
Pm";"Pl";"tRNA.2";"tRNA.3";"tRNA.4";"Pe (72.6)";"ipI";"Pe (73.0)";"57B";"57A";"Pm";" ↙
Pl";"1";"Pm";"3";"2   64";"4   50   65";"Pl";"Pl";"Pl";"53";"Pl";"5";"repEB";"repEA";" ↙
Pe";"5.1";"Pl";"segC";"5.3";"5.4";"6";"7";"Pl";"8";"Term";"Pl";"9";"Pl";"10";"11";" ↙
12";"wac";"13";"14";"Pl";"15";"Pl";"16";"Term";"17";"Pl";"17 A";"17 B";"17";"Pl";"18";" ↙
Pl";"Pl";"19";"Term";"Pl";"20";"Pl";"67";"68";"Pl";"21";"21";"Pl";"22";"Pl";"Pl";"23";" ↙
Term";"segD";"Pl";"24";"Term";"Term";"rnlB   24.1";"24.2";"24.3";"Term";"hoc";"inh";" ↙
Pl";"Pl";"segE";"Pl";"uvsW";"Term";"uvsY.-2";"Pl";"uvsY.-1";"uvsY";"Pm";"25";"26";" ↙
Pl";"26";"Pl";"Pl";"Pl";"51";"27";"28";"29";"48";"54";"Term";"alt.-3";"Pe";"alt.-2";" ↙
alt.-1";"alt";"Pl";"Term";"alt.1";"30";"Pm";"30.1";"30.2";"Pm";"30.3";"30.3";"30.4";" ↙
30.5";"30.6";"30.7";"Pe (128.2)";"30.8";"Pe (128.6)";"Term";"Term";"30.9";"rIII";"Pl";" ↙
31";"Pm";"31.1";"31.2";"cd";"cd.1";"cd.2";"Pe (131.7)";"Term";"cd.3";"cd.4";"cd.5";" ↙
pseT";"pseT.1";"pseT.2";"pseT.3";"alc";"Pe (134.4)";"Pl";"rnlA   63";"denA";"Term";" ↙
nrdB";"I-TevIII";"Pl";"Pm";"nrdB";"Pm";"nrdB.1";"Term";"mobE";"nrdA";"Pm";"nrdA.1";" ↙
nrdA.2";"td";"I-TevI";"Pl";"td";"Pm";"frd";"frd.1";"Term";"frd.2";"frd.3";"Pe ↙
(144.6)";"Term";"32";"Pl";"Pm";"segG   32.1";"59";"33";"dsbA";"Pm";"rnh";"Pe (148.6)";" ↙
Pl";"34";"Pm";"35";"Term";"Pl";"36";"Pl";"37";"Term";"38";"Pl";"t";"Term";"asiA";"Pe ↙
(158.7)";"asiA.1";"arn";"arn.1";"arn.2";"arn.3";"arn.4";"motA";"Pe (161.1)";"Term";" ↙
motA.1";"52";"52.1";"Term";"ac";"stp";"ndd";"ndd.1";"ndd.2";"ndd.2a";"ndd.3";"ndd.4";" ↙
Pe (164.2)";"ndd.5";"ndd.6";"Pe (164.5)";"denB";"Term";"denB.1";"rIIB"};
direction = {"-";"-";"-";"-";"-";"-";"-";"-";"-";"-";"-";"-";"-";"-";"-";"-";"-";"-";"- ↙
";"-";"-";"-";"-";"-";"-";"-";"-";"-";"-";"-";"-";"-";"-";"-";"-";"-";"-";"-";"-";"-";" ↙
-";"-";"-";"-";"-";"-";"-";"-";"-";"-";"-";"-";"-";"-";"-";"-";"-";"-";"-";"-";"-";"- ↙
";"-";"-";"-";"-";"-";"-";"-";"-";"-";"-";"-";"-";"-";"-";"-";"-";"-";"-";"-";"-";"-";" ↙
-";"-";"-";"-";"-";"-";"-";"-";"-";"-";"-";"-";"-";"-";"-";"-";"-";"-";"-";"-";"-";"- ↙
";"-";"-";"-";"-";"-";"-";"-";"-";"-";"-";"-";"-";"-";"-";"-";"-";"-";"-";"-";"-";"-";" ↙
-";"-";"-";"-";"-";"-";"-";"-";"-";"-";"-";"-";"-";"-";"-";"-";"-";"-";"-";"-";"-";"- ↙
";"-";"-";"-";"-";"-";"-";"-";"-";"-";"-";"-";"-";"-";"-";"-";"-";"-";"-";"-";"-";"-";" ↙
-";"-";"-";"-";"-";"-";"-";"-";"-";"-";"-";"-";"-";"-";"-";"-";"-";"-";"-";"-";"-";"- ↙
";"-";"-";"-";"-";"-";"-";"-";"-";"-";"-";"-";"-";"-";"-";"-";"-";"-";"-";"-";"-";"-";" ↙
-";"-";"-";"-";"-";"-";"-";"-";"-";"-";"-";"-";"-";"-";"-";"-";"-";"-";"-";"+";"+";" ↙
+";"+";"+";"-";"-";"-";"+";"+";"+";"+";"+";"+";"+";"+";"+";"+";"+";"+";"+";"+";"+";" ↙
+";"+";"+";"+";"+";"+";"+";"+";"+";"+";"+";"+";"+";"+";"+";"+";"+";"+";"+";"+";"+";" ↙
+";"+";"+";"+";"+";"+";"+";"+";"+";"+";"+";"+";"+";"-";"+";"+";"+";"+";"-";"-";"-";"- ↙
";"-";"-";"-";"+";"+";"+";"+";"-";"-";"-";"-";"-";"-";"-";"-";"-";"-";"-";"-";"+";"+";" ↙
+";"+";"+";"+";"+";"+";"-";"-";"-";"-";"-";"-";"-";"-";"-";"";"-";"-";"";"-";"-";"-";"- ↙
";"-";"-";"-";"-";"-";"-";"-";"-";"-";"-";"-";"";"-";"-";"-";"-";"-";"-";"-";"-";"-";"- ↙
";"-";"-";"-";"-";"-";"-";"+";"-";"-";"-";"-";"-";"-";"-";"-";"-";"-";"-";"-";"-";"-";" ↙
```

```
-";"-";"-";"-";"-";"-";"-";"-";"-";"-";"-";"-";"-";"-";"-";"-";"-";"-";"-";"-";"-";"-";"- ↙
";"-";"-";"+";"+";"-";"+";"+";"+";"+";"+";"+";"+";"+";"+";"+";"+";"-";"-";"-";"-";"-";" ↙
-";"-";"-";"-";"-";"-";"-";"-";"-";"-";"-";"-";"-";"-";"-";"-";"-";"-";"-";"-";"-";"- ↙
";"-";"-";"-";"-"};
len = {"2178";"";"204";"";"345";"138";"381";"114";"";"1551";"";"";"198";"138";"426";" ↙
216";"489";"";"360";"501";"";"684";"243";"246";"1320";"312";"747";"";"603";"624";"";" ↙
183";"471";"165";"204";"486";"342";"207";"";"";"243";"";"";"675";"";"516";"";"";"780";" ↙
1029";"";"165";"627";"294";"";"258";"183";"";"1428";"";"345";"1176";"";"666";"";" ↙
1056";"741";"";"252";"378";"";"";"2697";"";"";"369";"564";"960";"";"687";"";"390";" ↙
189";"";"1683";"";"207";"264";"";"1020";"";"141";"";"1203";"795";"";"";"192";"204";" ↙
318";"217";"558";"";"264";"327";"";"240";"132";"";"294";"183";"";"";"309";"213";"";" ↙
471";"";"633";"1280";"777";"";"538";"";"318";"474";"";"";"486";"";"156";"321";"309";" ↙
264";"243";"315";"927";"1002";"";"1023";"828";"402";"528";"";"303";"978";"";"";"1011";" ↙
780";"546";"105";"";"117";"195";"183";"189";"";"";"387";"294";"213";"";"582";"189";" ↙
186";"213";"";"468";"348";"546";"462";"";"279";"267";"177";"363";"330";"660";"417";"";" ↙
303";"";"582";"";"495";"";"";"";"456";"309";"363";"393";"609";"";"594";"";"336";"";" ↙
264";"";"";"139";"119";"75";"666";"74";"76";"87";"78";"74";"88";"75";"";"";"288";" ↙
411";"186";"";"288";"";"459";"243";"";"";"726";"";"531";"825";"453";"";"";"";"591";"";" ↙
1728";"138";"153";"";"495";"";"423";"171";"294";"1983";"3099";"";"1005";"";"";" ↙
867";"";"1809";"660";"1584";"1464";"930";"771";"";"819";"";"495";"";"1833";"";"1572";" ↙
1518";"1251";"";"1980";"";"";"492";"";"";"1575";"";"243";"426";"";"639";"573";"";" ↙
810";"";"";"1566";"";"672";"";"1284";"";"";"1005";"279";"171";"";"1317";"681";"";"";" ↙
618";"";"1764";"";"168";"";"225";"414";"";"399";"288";"";"627";"";"";"";"750";"1176";" ↙
534";"1773";"1095";"963";"";"291";"";"204";"186";"2049";"";"";"189";"1464";"";"270";" ↙
837";"";"228";"459";"207";"198";"288";"366";"";"333";"";"";"";"177";"249";"";"336";"";" ↙
309";"237";"582";"339";"231";"";"";"276";"201";"228";"906";"228";"300";"354";" ↙
504";"";"";"1125";"435";"";"503";"294";"";"";"664";"";"252";"";"426";"2265";"";"327";" ↙
264";"312";"738";"";"549";"";"582";"243";"";"387";"228";"";"";"906";"";"";"633";"654";" ↙
339";"270";"";"918";"";"";"3870";"";"1119";"";"";"666";"";"3081";"";"552";"";"657";"";" ↙
273";"";"153";"279";"132";"297";"459";"207";"636";"";"";"150";"1329";"141";"";"156";" ↙
81";"456";"216";"111";"123";"81";"129";"";"99";"87";"";"558";"";"195";"939"};

n=1;
genes = cell(1, 3);
Pattern = ["Pe","Pm","Pl"];
for i=1:length(gene)
    if (len{i} ~= "")
        genes{n, 1} = gene{i};
        promoter = "";
        if (strcmp(direction{i}, "-"))
           for j=i+1:1:length(gene)
                if (startsWith(gene{j}, Pattern) && strcmp(direction{j}, direction{i}))
                    promoter = gene{j};
                    break;
                end
           end
        else
            for j=i-1:-1:1
                if (startsWith(gene{j}, Pattern) && strcmp(direction{j}, direction{i}))
                    promoter = gene{j};
                    break;
                end
            end
        end
        genes{n, 2} = promoter;
        genes{n, 3} = direction{i};
```

```
        n=n+1;
    end
end
```
