# Supplementary material for: Bio-informatic analysis of CRISPR protospacer adjacent motifs (PAMs) in T4 genome
Source: BMC Genom Data. 2022 Jun 2;23:40. doi: 10.1186/s12863-022-01056-8 (PMC9161530; doi:10.1186/s12863-022-01056-8)
Supplement: Supplementary file 1 — Additional file 1. [file 12863_2022_1056_MOESM1_ESM.zip › getGenesFunctions.pdf]

```
function functionsList = getGenesFunctions(genesList)
    functionsList = strings(length(genesList), 1);
    genesListForFunctions(:, 1) = ["rIIA";"60";"mobA";"39";"goF = comC? = go9H";"cef = ↙
mb = M1 = motC";"pseF = plaCTr5x?";"motB";"dexA";"dda = sud";"srd = dda.2";"modA";" ↙
modB";"srh = modA.5";"mrh";"soc";"segF = 69";"56";"oriA";"dam";"61 = 58";"sp = 61.3 = ↙
rIV";"dmd = 61.5";"41";"40";"uvsX = fdsA";"segA";"?-gt";"42";"imm";"43";"regA";"62";" ↙
44";"45";"rpbA";"46";"47";"?-gt";"mobB";"55";"nrdH = 55.7";"nrdG = 55.9";"mobC = ↙
55.10";"nrdD = sunY";"I-TevII";"49";"49'";"pin";"nrdC";"mobD";"rI = tk.-2";"tk";"vs";" ↙
regB";"denV";"ipII";"ipIII";"e";"nudE = e.1";"goF3";"rnaC = species 1";"rnaD = species ↙
2";"tRNA Arg";"segB";"tRNA Ile";"tRNA Thr";"tRNA Ser";"tRNA Pro";"tRNA Gly";"tRNA ↙
Leu";"tRNA Gln";"ip1";"57B";"57A";"1";"3";"2 = 64";"4 = 50 = 65";"53";"5";"oriE";" ↙
repEB";"repEA";"segC";"6";"7";"8";"9";"10";"11";"12";"wac";"13";"14";"15";"16";"16'";" ↙
17";"17'A";"17'B";"17""";"18";"19";"20";"pip = 67";"68";"21";"21'";"22";"23";"segD";"24 ↙
= os";"rnlB = 24.1";"hoc = eph";"inh = lip";"segE";"uvsW = dar";"uvsY = fdsB";"oriF = ↙
oriuvsY";"25";"26";"26'";"26""";"51";"27";"28";"29";"48";"54";"alt";"30 = lig";"rIII";" ↙
31";"cd";"pseT";"alc = unf";"rnlA = 63";"denA";"nrdB";"I-TevIII";"mobE";"nrdA";"td";"I- ↙
TevI";"frd";"32";"segG = 32.1";"59";"33";"dsbA";"rnh = das";"34";"oriG = ori34";"35";" ↙
36";"37";"38";"t = rV = stII";"asiA";"arn";"motA = sip";"52";"ac";"ama = rs";"stp";"ndd ↙
= D2b";"pla262";"denB";"rIIB"];
    genesListForFunctions(:, 2) = ["Membrane-associated protein; affect host membrane ↙
ATPase";"DNA topoisomerase subunit";"Pseudogene of Mob site-specific DNA ↙
endonuclease";"DNA topoisomerase subunit; DNA-dependent ATPase; membrane-associated ↙
protein";"Affects mRNA metabolism";"Processing of T4 tRNAs";"5? phosphatase";"";" ↙
Exonuclease A";"DNA helicase; DNA-dependent ATPase";"Postulated decoy of host ?70 or ? ↙
S";"Adenylribosylating enzyme";"Adenylribosylating enzyme";"Postulated decoy of host ? ↙
32";"Affects phosphorylation of host ?32";"Small outer capsid protein";"Intron-like ↙
endonuclease. A probable fusion protein, generated from 56 and 69 by hopping of ↙
ribosomes across a pseudoknot, is larger";"dCTPase; dUTPase; dCDPase; dUDPase";"DNA ↙
replication origin; cis-acting sequences in 56, 69, and soc; primer transcript same as ↙
transcript for these genes";"DNA adenine methylase";"Primase; requires interaction with ↙
gp41 helicase for priming at unique sequence";"Periplasmic protein";"Discriminator of ↙
mRNA degradation";"Replicative and recombination DNA helicase; GTPase; ATPase; dGTPase; ↙
dATPase";"Membrane-associated protein initiator of head vertex";"RecA-like ↙
recombination protein; DNA-ATPase";"Site-specific intron-like DNA endonuclease";"?- ↙
Glucosyltransferase";"dCMP hydroxymethylase";"Inner membrane protein";"DNA polymerase; ↙
3?-to-5? exonuclease";"Translational repressor of several early genes";"Clamp-loader ↙
subunit";"Clamp-loader subunit";"Processivity enhancing sliding clamp of DNA ↙
polymerase; and mobile enhancer of late promoters";"RNAP-binding protein";" ↙
Recombination protein and nuclease subunit";"Recombination protein and nuclease ↙
subunit";"?-Glucosyltransferase";"Putative site-specific intron-like DNA ↙
endonuclease";"? factor recognizing late T4 promoters";"Anaerobic nucleotide reductase ↙
subunit";"Anaerobic nucleotide reductase subunit";"Putative intron-like DNA ↙
endonuclease";"Anaerobic ribonucleotide reductase subunit; RNA contains a self-splicing ↙
intron";"Endonuclease for nrdD-intron homing";"Recombination endonuclease VII";" ↙
Internal translation initiation product";"Inhibitor of host Lon protease";"Thioredoxin, ↙
glutaredoxin";"Putative site-specific DNA endonucleasee";"Membrane protein";"Thymidine ↙
kinase";"Modifier of valyl-tRNA synthetase";"Site-specific RNase";"Endonuclease V; N- ↙
glycosidase";"Internal protein II";"Internal protein III";"Soluble lysozyme; ↙
endolysin";"Nudix hydrolase";"";"Stable RNA";"Stable RNA";"";"Probable site-specific ↙
intron-like DNA endonuclease";"";"";"";"";"";"";"";"Internal protein 1";"";"Chaperone ↙
of long and short tail fiber assembly";"dNMP kinase";"Head-proximal tip of tail tube";" ↙
Protein protecting DNA ends";"Head completion protein";"Base plate wedge component";" ↙
Base plate lysozyme; hub component";"DNA replication origin; cis-acting sequences in ↙
genes 4, 53, 5; primer transcript in opposite orientation of gene 5 transcripts";" ↙
Protein required for initiation from oriE";"Protein auxiliary for initiation from ↙
oriE";"Site-specific intron-like DNA endonuclease";"Base plate wedge component";"Base ↙
```

```
plate wedge component";"Base plate wedge component";"Base plate wedge component, tail ↙
fiber socket, trigger for tail sheath contraction";"Base plate wedge component, tail ↙
pin";"Base plate wedge component, tail pin, interface with short tail fibers, gp12";" ↙
Short tail fibers";"Whiskers, facilitate long tail fiber attachment";"Head ↙
completion";"Head completion";"Proximal tail sheath stabilizer, connector to gp3 and/or ↙
gp19";"Terminase subunit, binds dsDNA";"Truncated C-terminal end";"Terminase subunit ↙
with nuclease and ATPase activity; binds single-stranded DNA, gp16 and gp20";"Terminase ↙
subunits with nuclease and";"ATPase activity; internal transcription and translation in ↙
frame; does not bind ssDNA";"Terminase subunit with nuclease and ATPase activity ↙
(transcript processing and internal initiation of translation in frame); does not bind ↙
ssDNA; several additional proteins most likely initiated from internal ribosome binding ↙
sites of the 17 transcripts";"Tail sheath monomer";"Tail tube monomer";"Portal vertex ↙
protein of the head";"Prohead core protein; precursor to internal peptides";"Prohead ↙
core protein";"Prohead core protein and protease";"Prohead core protein and protease ↙
(internal initiation of translation)";"Prohead core protein; precursor to internal ↙
peptides";"Precursor of major head subunit";"Probable site-specific intron-like DNA ↙
endonuclease";"Precursor of head vertex subunit";"Second RNA ligase";"Large outer ↙
capsid protein";"Minor capsid protein; inhibitor of gp21 protease";"Probable site- ↙
specific intron-like DNA endonuclease";"RNA-DNA- and DNA-helicase; DNA-dependent ↙
ATPase";"ssDNA binding, recombination and repair protein; helper of UvsX, inhibitor of ↙
endoVII";"DNA replication origin; cis-acting sequences in genes uvsY, uvsY.-1 and uvsY. ↙
-2; primer transcript same as uvsY, uvsY.-1 and uvsY.-2 transcript";"Base plate wedge ↙
subunit";"Base plate hub subunit";"Internal in-frame translation initiation";"Internal ↙
out-of-frame translation initiation";"Base plate hub assembly catalyst?";"Base plate ↙
hub subunit";"Base plate distal hub subunit";"Base plate hub; determinant of tail ↙
length";"Base plate; tail tube associated";"Base plate-tail tube initiator";" ↙
Adenosylribosyltransferase (packaged and injected with DNA)";"DNA ligase";"Unknown";" ↙
Cochaperonin for GroEL";"dCMP deaminase";"Deoxyribonucleotide3' phosphatase, 5' ↙
polynucleotide kinase";"RNA polymerase- and DNA-binding protein; transcription ↙
terminator on dC-containing DNA";"RNA ligase; catalyst of tail fiber attachment";" ↙
Endonuclease II that restricts dCcontaining DNA";"Ribonucleotide reductase ? subunit ↙
(contains intron)";"Defective intron homing endonuclease";"Putative mobile ↙
endonuclease";"Ribonucleotide reductase ? subunit";"Thymidylate synthetase (contains ↙
intron)";"Intron-homing endonuclease";"Dihydrofolate reductase";"ssDNA-binding protein, ↙
scaffold of DNA replication, recombination and DNA precursor-synthesizing protein ↙
machines";"Site-specific DNA endonuclease; localized gene conversion, exclusion";" ↙
Loader of gene 41 DNA helicase, ssDNA-binding protein";"Protein connecting gp45 and ↙
gp55, to allow transcription by RNA polymerase from late promoters";"dsDNA binding ↙
protein";"RNase H; 5? to 3? DNase; yeast FEN homologue";"Proximal tail fiber subunit";" ↙
DNA replication origin; primer transcript in opposite orientation of 34 transcript";" ↙
Tail fiber hinge";"Small distal tail fiber subunit";"Large distal tail fiber subunit";" ↙
Assembly catalyst of distal tail fiber";"Holin, inner membrane pore protein, affects ↙
lysis timing and inhibition";"Protein that binds to host ?70, inhibits interaction with ↙
?35 regions of classical promoters, and facilitates interaction with T4 MotA protein";" ↙
Inhibitor of MrcBC restriction nuclease";"Activator of middle promoters; dsDNA binding ↙
protein specific for mot boxes";"DNA topoisomerase subunit; membrane-associated ↙
protein";"Membrane protein";"";"Peptide modulating host restriction system";"Protein ↙
that disrupts host nucleoid; binds to host HU";"Unknown";"Endonuclease IV, single- ↙
strand-specific endonuclease";"Membrane-associated protein; affects host membrane ↙
ATPase"];

    for i=1:length(genesList)
        found = false;
        for j=1:length(genesListForFunctions)
            terms = strtrim(strsplit(genesListForFunctions(j,1), '='));
            index = strcmp(terms, genesList(i));
```

```
            if (sum(index) ~= 0)
                found = true;
                functionsList(i) = genesListForFunctions(j, 2);
                break;
            end
        end
        if (~found)
            if (contains(genesList(i), "."))
                geneNameWithoutDots = extractBefore(genesList(i), ".");
                for j=1:length(genesListForFunctions)
                    terms = strtrim(strsplit(genesListForFunctions(j,1), '='));
                    index = strcmp(terms, geneNameWithoutDots);
                    if (sum(index) ~= 0)
                        found = true;
                        functionsList(i) = genesListForFunctions(j, 2);
                        break;
                    end
                end
            end
        end
        if (~found)
            functionsList(i) = "unkown gene";
        end
    end
end
```
