# Supplementary material for: Bio-informatic analysis of CRISPR protospacer adjacent motifs (PAMs) in T4 genome
Source: BMC Genom Data. 2022 Jun 2;23:40. doi: 10.1186/s12863-022-01056-8 (PMC9161530; doi:10.1186/s12863-022-01056-8)
Supplement: Supplementary file 1 — Additional file 1. [file 12863_2022_1056_MOESM1_ESM.zip › getNucleotidesCombinations.pdf]

```
function combinations = getNucleotidesCombinations(possibleNucleotides, ↙
numberOfRequestedNucleotides)
    combinations = possibleNucleotides;
    for i=2:numberOfRequestedNucleotides
        temp = strings(length(combinations), length(possibleNucleotides));
        for j=1:length(possibleNucleotides)
            temp(:, j) = strcat(combinations, possibleNucleotides(j));
        end
        combinations = reshape(temp, [length(combinations)*length(possibleNucleotides) ↙
1]);
    end
end
```
