# Supplementary material for: Bio-informatic analysis of CRISPR protospacer adjacent motifs (PAMs) in T4 genome
Source: BMC Genom Data. 2022 Jun 2;23:40. doi: 10.1186/s12863-022-01056-8 (PMC9161530; doi:10.1186/s12863-022-01056-8)
Supplement: Supplementary file 1 — Additional file 1. [file 12863_2022_1056_MOESM1_ESM.zip › getNumberOfPromotersContainPAMs.pdf]

```
function [earlyPromotersResults, middlePromotersResults, latePromotersResults, ↙
earlyPromotersSequences, middlePromotersSequences, latePromotersSequences] = ↙
getNumberOfPromotersContainPAMs(phageSeq, PAMs, promoters, promotersLoc, ↙
promotersDirection, promoterLengthArray, providedTickAngle)
    if (nargin < 7)
        plotFigures = false;
    else
        plotFigures = true;
        xtickAngleVar = providedTickAngle;
    end

    %early promoter ************************
    earlyPromotersIndecies = startsWith(promoters, "Pe");
    startPosEarlyPromoters = promotersLoc(earlyPromotersIndecies);
    directionEarlyPromoters = promotersDirection(earlyPromotersIndecies);

    previousCount = 0;
    earlyPromotersResults = zeros(length(PAMs), length(promoterLengthArray));
    for i =1:length(promoterLengthArray)
        [earlyPromotersResultsTemp, earlyPromotersSequences] = promotersPAMs ↙
(promoterLengthArray(i), phageSeq, startPosEarlyPromoters, directionEarlyPromoters, ↙
PAMs);
        earlyPromotersResults(:, i) = earlyPromotersResultsTemp.'-previousCount;
        previousCount = earlyPromotersResultsTemp.';
    end

    if (plotFigures)
        promotersFigure = figure('Position', [50 50 1200 800]);
        subplot(3, 1, 1, 'Parent', promotersFigure)
        [~, Indecies] = sort(sum(earlyPromotersResults, 2));
        bar(earlyPromotersResults(Indecies, :), 'stacked');
        title(strcat("PAM in early promoters (", num2str(length ↙
(startPosEarlyPromoters)), " promoters)"));
        set(gca,'XTick',1:length(PAMs),'XTickLabel',PAMs(Indecies));
        xtickangle(xtickAngleVar);
        ylim([0 length(startPosEarlyPromoters)]);
        legend("0-10", "10-20", "20-30", "30-40", "40-50", "50-60", ↙
'Location','northwest');
    end


    %middle promoter ************************
    middlePromotersIndecies = startsWith(promoters, "Pm");
    startPosMiddlePromoters = promotersLoc(middlePromotersIndecies);
    directionMiddlePromoters = promotersDirection(middlePromotersIndecies);

    previousCount = 0;
    middlePromotersResults = zeros(length(PAMs), length(promoterLengthArray));
    for i =1:length(promoterLengthArray)
        [middlePromotersResultsTemp, middlePromotersSequences] = promotersPAMs ↙
(promoterLengthArray(i), phageSeq, startPosMiddlePromoters, directionMiddlePromoters, ↙
PAMs);
        middlePromotersResults(:, i) = middlePromotersResultsTemp.'-previousCount;
        previousCount = middlePromotersResultsTemp.';
    end
```

```
    if (plotFigures)
        subplot(3, 1, 2, 'Parent', promotersFigure)
        [~, Indecies] = sort(sum(middlePromotersResults, 2));
        bar(middlePromotersResults(Indecies, :), 'stacked');
        title(strcat("PAM in middle promoters (", num2str(length ↙
(startPosMiddlePromoters)), ") promoters"));
        set(gca,'XTick',1:length(PAMs),'XTickLabel',PAMs(Indecies));
        xtickangle(xtickAngleVar);
        ylim([0 length(startPosMiddlePromoters)]);
        legend("0-10", "10-20", "20-30", "30-40", "40-50", "50-60", ↙
'Location','northwest');
    end


    %late promoters
    latePromotersIndecies = startsWith(promoters, "Pl");
    startPosLatePromoters = promotersLoc(latePromotersIndecies);
    directionLatePromoters = promotersDirection(latePromotersIndecies);

    previousCount = 0;
    latePromotersResults = zeros(length(PAMs), length(promoterLengthArray));
    for i =1:length(promoterLengthArray)
        [latePromotersResultsTemp, latePromotersSequences] = promotersPAMs ↙
(promoterLengthArray(i), phageSeq, startPosLatePromoters, directionLatePromoters, ↙
PAMs);
        latePromotersResults(:, i) = latePromotersResultsTemp.'-previousCount;
        previousCount = latePromotersResultsTemp.';
    end

    if (plotFigures)
        subplot(3, 1, 3, 'Parent', promotersFigure)
        [~, Indecies] = sort(sum(latePromotersResults, 2));
        bar(latePromotersResults(Indecies, :), 'stacked');
        title(strcat("PAM in late promoters (", num2str(length(startPosLatePromoters)), ↙
") promoters"));
        set(gca,'XTick',1:length(PAMs),'XTickLabel',PAMs(Indecies));
        xtickangle(xtickAngleVar);
        ylim([0 length(startPosLatePromoters)]);
        legend("0-10", "10-20", "20-30", "30-40", "40-50", "50-60", ↙
'Location','northwest');
    end
end
```
