# Supplementary material for: Bio-informatic analysis of CRISPR protospacer adjacent motifs (PAMs) in T4 genome
Source: BMC Genom Data. 2022 Jun 2;23:40. doi: 10.1186/s12863-022-01056-8 (PMC9161530; doi:10.1186/s12863-022-01056-8)
Supplement: Supplementary file 1 — Additional file 1. [file 12863_2022_1056_MOESM1_ESM.zip › getPAMinGenes.pdf]

```
function [results] = getPAMinGenes(phageSeq, geneStartEnd, PAMs, direction, ↙
genesLength, genes, essential)
%in genes **********************
    revPAMs = negativeStrandAndReverse(PAMs);

    results = zeros(length(geneStartEnd), length(PAMs));

    for pamInd = 1:length(PAMs)
        n = strlength(PAMs{pamInd});
        m = 0;
        listOfCurrentGenes = zeros(1, 1);
        lastAddedIndex = 0;
        for i=1:strlength(phageSeq)-n

            %adding
            for j = lastAddedIndex+1:length(geneStartEnd)
                if (i == geneStartEnd(j, 1))
                    m = m+1;
                    listOfCurrentGenes(m) = j; %add genes that we entered
                    lastAddedIndex = j;
                else
                    if (i >= geneStartEnd(j, 2))
                        break;
                    end
                end
            end

            %removing
            if (m ~= 0)
                genesIndeicesForElemination = i> geneStartEnd(listOfCurrentGenes, 2);
                m = m - sum(genesIndeicesForElemination);
                listOfCurrentGenes(genesIndeicesForElemination) = []; %remove genes ↙
that we have passed
            end

            if (m == 0)
                continue; %if in a non coding area move to next nucleotide
            else
                %adjust (could be un-nessecary)
            end

            positiveStrand = compareNucleotidesSeq(PAMs{pamInd}, (phageSeq(i:i+n-1)));
            negativeStrand = compareNucleotidesSeq(revPAMs{pamInd}, (phageSeq(i:i+n- ↙
1)));

            for j=1:length(listOfCurrentGenes)
                if ((direction(listOfCurrentGenes(j)) == '+') && positiveStrand)
                    results(listOfCurrentGenes(j), pamInd) = results(listOfCurrentGenes ↙
(j), pamInd) + 1;
                end
                if ((direction(listOfCurrentGenes(j)) == '-') && negativeStrand)
                    results(listOfCurrentGenes(j), pamInd) = results(listOfCurrentGenes ↙
(j), pamInd) + 1;
                end
            end
        end
```

```
    end

    normalizedResults = results./genesLength;

    numberOfFigures = fix(size(normalizedResults,2)/4)+1;
    for i=1:numberOfFigures
        figure();
        lastpltInd = 4;
        if (i==numberOfFigures)
            lastpltInd = mod(size(normalizedResults,2),4);
        end
        for j=1:lastpltInd
            subplot(4,1,j);
            bar(normalizedResults(:,(i-1)*4+j));
            ylim([0 0.3]);
            title(PAMs{(i-1)*lastpltInd + j});
            set(gca,'XTick',1:length(genes),'XTickLabel',genes);
        end
    end
    %end of in genes **********************

    means = mean(normalizedResults);
    largerThanMean = bsxfun(@gt, normalizedResults, means);
    outliers = isoutlier(normalizedResults, 1); %dimension = 1 means per column
    significant = largerThanMean & outliers;

    fileName = 'importantGenesPerPAM';

    ImportantGenes = cell(1, size(significant,2));
    for i=1:size(significant,2)
        impGenes = genes(significant(:,i));
        imEssentials = essential(significant(:,i));
        imEssentialsWords = strings(size(imEssentials,1), size(imEssentials,2));
        for j=1:length(imEssentials)
            if (imEssentials(j) == 1)
                imEssentialsWords(j) = "essential";
            else
                imEssentialsWords(j) = "not essential";
            end
        end
        impFunctions = getGenesFunctions(impGenes);
        ImportantGenes{i} = [impGenes, impFunctions, imEssentialsWords];

        if (~isempty(ImportantGenes{i}))
            xlswrite(fileName, ImportantGenes{i}, PAMs(i)); %a sheet for each PAM
        end
    end
end
```
