# Supplementary material for: Bio-informatic analysis of CRISPR protospacer adjacent motifs (PAMs) in T4 genome
Source: BMC Genom Data. 2022 Jun 2;23:40. doi: 10.1186/s12863-022-01056-8 (PMC9161530; doi:10.1186/s12863-022-01056-8)
Supplement: Supplementary file 1 — Additional file 1. [file 12863_2022_1056_MOESM1_ESM.zip › negativeStrandAndReverse.pdf]

```
function reversedSeq = negativeStrandAndReverse(seq)

    for i=1:length(seq)
        reversedSeq(i) = reverse(seq(i));
        reversedSeq(i) = strrep(reversedSeq(i), "T", "a");
        reversedSeq(i) = strrep(reversedSeq(i), "A", "t");
        reversedSeq(i) = strrep(reversedSeq(i), "C", "g");
        reversedSeq(i) = strrep(reversedSeq(i), "G", "c");
        reversedSeq(i) = strrep(reversedSeq(i), "U", "a");
        reversedSeq(i) = strrep(reversedSeq(i), "R", "y");
        reversedSeq(i) = strrep(reversedSeq(i), "Y", "r");
        reversedSeq(i) = strrep(reversedSeq(i), "K", "m");
        reversedSeq(i) = strrep(reversedSeq(i), "M", "k");
        reversedSeq(i) = strrep(reversedSeq(i), "B", "v");
        reversedSeq(i) = strrep(reversedSeq(i), "D", "h");
        reversedSeq(i) = strrep(reversedSeq(i), "H", "d");
        reversedSeq(i) = strrep(reversedSeq(i), "V", "b");
        reversedSeq(i) = convertStringsToChars(upper(reversedSeq(i)));
    end
end
```
