# Supplementary material for: Bio-informatic analysis of CRISPR protospacer adjacent motifs (PAMs) in T4 genome
Source: BMC Genom Data. 2022 Jun 2;23:40. doi: 10.1186/s12863-022-01056-8 (PMC9161530; doi:10.1186/s12863-022-01056-8)
Supplement: Supplementary file 1 — Additional file 1. [file 12863_2022_1056_MOESM1_ESM.zip › PAMS2AA.pdf]

```
close all;

PAMs = unique(["NGG";"NAG";"NGRRT";"NGRRN";"NNGRRT";"NGGRRT";"TTGAAT";"TTGGGT";" ↙
NNNNGATT";"NNNNRYAC";"NNAGAAW";"NGGNG";"TTTV";"TCTA";"GTTV";"GCTV";"TATA";"TTTV";" ↙
CTT";"ATG";"AWG";"TCN";"CCN";]);

for i=1:length(PAMs)
    PAMs(i) = erase(PAMs(i), "NNN");
end

aaSingleLetters = ["A", "R", "N", "D", "C", "Q", "E", "G", "H", "I", "L", "K", "M", ↙
"F", "P", "S", "T", "W", "Y", "V"];
aaThreeLetters = ["ala", "arg", "asn", "asp", "cys", "gln", "glu", "gly", "his", "ile", ↙
"leu", "lys", "met", "phe", "pro", "ser", "thr", "trp", "tyr", "val"];

aminos = strings(length(PAMs), 2);
aminos(:, 1) = PAMs;
aminosAll = strings;
medianASA = zeros(size(PAMs));
meanASA = zeros(size(PAMs));
maxMedianASA = zeros(size(PAMs));
maxMeanASA = zeros(size(PAMs));
for i=1:length(PAMs)
    [~, aa] = getAllAAFromPAM(PAMs(i));
    [medianASA(i), meanASA(i), maxMedianASA(i), maxMeanASA(i)] = getASA(aa);
    aminos(i, 2) = join(aa);
    aminosAll = horzcat(aminosAll, aa);
end
aminosAll(1) = [];

bins = unique(aminosAll,'stable');
counts=cellfun(@(x) sum(ismember(aminosAll,x)),bins,'un',0);

% fig1 = figure();
% [sortedCounts, Ind] = sort([counts{:}]);
% bar(sortedCounts);
% % bar([counts{:}]);
% set(gca, 'XTick', 1:1:20);
% set(gca, 'XTickLabel', bins(Ind));
% % set(gca, 'XTickLabel', bins);
% title("Number of hits that aminoacids codons can be targeted by the selected PAMs")

% fig2 = figure();
% uit0 = uitable(fig2);
% set(uit0, 'ColumnWidth', num2cell([100 300]));
% uit0.Data =  cellstr(aminos);
% uit0.Units = 'normalized';
% uit0.Position = [0 0 1 1];

midPoint = fix(length(PAMs)/2);
fig3 = figure();
subplot(3,1,1);
hb = bar([medianASA(1:midPoint), meanASA(1:midPoint)]), legend('Avg median ASA','Avg ↙
mean ASA');
hb(1).FaceColor = 'w';
hb(2).FaceColor = 'k';
```

```
set(gca, 'XTick', 1:1:length(PAMs(1:midPoint)));
set(gca, 'XTickLabel', PAMs(1:midPoint));
title("Average median ASA and avergae mean ASA for each PAM.")
xtickangle(45);
ylim([5 102]);

% fig4 = figure();
subplot(3,1,2);
hb = bar([medianASA(midPoint+1:length(PAMs)), meanASA(midPoint+1:length(PAMs))]), ↙
legend('Avg median ASA','Avg mean ASA');
hb(1).FaceColor = 'w';
hb(2).FaceColor = 'k';
set(gca, 'XTick', 1:1:length(PAMs)-midPoint);
set(gca, 'XTickLabel', PAMs(midPoint+1:length(PAMs)));
title("Average median ASA and avergae mean ASA for each PAM.")
xtickangle(45);
ylim([5 102]);

fig5 = figure();
subplot(3,1,1);
hb = bar([maxMedianASA(1:midPoint), maxMeanASA(1:midPoint)]), legend('Max median ↙
ASA','Max mean ASA');
hb(1).FaceColor = 'w';
hb(2).FaceColor = 'k';
set(gca, 'XTick', 1:1:length(PAMs(1:midPoint)));
set(gca, 'XTickLabel', PAMs(1:midPoint));
title("Max median ASA and Max mean ASA for each PAM.")
xtickangle(45);
ylim([5 102]);

% fig6 = figure();
subplot(3,1,2);
hb = bar([medianASA(midPoint+1:length(PAMs)), meanASA(midPoint+1:length(PAMs))]), ↙
legend('Max median ASA','Max mean ASA');
hb(1).FaceColor = 'w';
hb(2).FaceColor = 'k';
set(gca, 'XTick', 1:1:length(PAMs)-midPoint);
set(gca, 'XTickLabel', PAMs(midPoint+1:length(PAMs)));
title("Max median ASA and Max mean ASA for each PAM.")
xtickangle(45);
ylim([5 102]);
```
