# Supplementary material for: Bio-informatic analysis of CRISPR protospacer adjacent motifs (PAMs) in T4 genome
Source: BMC Genom Data. 2022 Jun 2;23:40. doi: 10.1186/s12863-022-01056-8 (PMC9161530; doi:10.1186/s12863-022-01056-8)
Supplement: Supplementary file 1 — Additional file 1. [file 12863_2022_1056_MOESM1_ESM.zip › promotersPAMs.pdf]

```
function [results, promotersSequences] = promotersPAMs(promoter_length, phage_DNA_seq, ↙
start_pos_promoters, direction_promoters, PAMs)
%PROMOTERSPAMS Summary of this function goes here
%   Detailed explanation goes here

promotersSequences = strings(length(start_pos_promoters), 1);
inPromotersResults = zeros(length(start_pos_promoters), length(PAMs));

for i=1:length(start_pos_promoters)
    if (direction_promoters(i) == "-")
        promotersSequences(i) = convertCharsToStrings(phage_DNA_seq(start_pos_promoters ↙
(i):start_pos_promoters(i)+promoter_length));
        for j=start_pos_promoters(i):start_pos_promoters(i)+promoter_length
            for k=1:length(PAMs)
                if (start_pos_promoters(i)+promoter_length-j >= strlength(PAMs(k))-1)
                    if (compareNucleotidesSeq(phage_DNA_seq(j:j+strlength(PAMs(k))-1), ↙
negativeStrandAndReverse(PAMs(k))))
                        inPromotersResults(i, k) = true;
                    end
                end
            end
        end
    else
        promotersSequences(i) = convertCharsToStrings(phage_DNA_seq(start_pos_promoters ↙
(i):start_pos_promoters(i)+promoter_length));
        for j=start_pos_promoters(i)-promoter_length:start_pos_promoters(i)
            for k=1:length(PAMs)
                if (start_pos_promoters(i)-j >= strlength(PAMs(k))-1)
                    if (compareNucleotidesSeq(phage_DNA_seq(j:j+strlength(PAMs(k))-1), ↙
PAMs(k)))
                        inPromotersResults(i, k) = true;
                    end
                end
            end
        end
    end
end

results = sum(inPromotersResults, 1);
% figure();
% bar(results);
% title("PAM in early promoters");
% set(gca,'XTick',1:length(PAMs),'XTickLabel',PAMs);
% xtickangle(45);
end
```
