# Supplementary material for: Bio-informatic analysis of CRISPR protospacer adjacent motifs (PAMs) in T4 genome
Source: BMC Genom Data. 2022 Jun 2;23:40. doi: 10.1186/s12863-022-01056-8 (PMC9161530; doi:10.1186/s12863-022-01056-8)
Supplement: Supplementary file 1 — Additional file 1. [file 12863_2022_1056_MOESM1_ESM.zip › removeNsFromEnds.pdf]

```
function [PAMAfterRemoval] = removeNsFromEnds(PAM)
%REMOVENSFROMENDS Summary of this function goes here
%   Detailed explanation goes here
    PAMAfterRemoval = PAM;
    while (startsWith(PAMAfterRemoval, "N"))
        PAMAfterRemoval = extractAfter(PAMAfterRemoval,1);
    end

    while (endsWith(PAMAfterRemoval, "N"))
        PAMAfterRemoval = extractBefore(PAMAfterRemoval, strlength(PAMAfterRemoval));
    end
end
```
